# Prevalence of low-intake dehydration in hospitalised older adults: systematic review and meta-analysis

**DOI:** 10.1136/bmjph-2025-002985

**Published:** 2026-05-12

**Authors:** Lee Hooper, Ellice Parkinson, Asmaa Saber Abdelhamid, Ke Deng, Onyekwutozia Edozie, Charlotte Fenner, Lydia Frost, Miruna Ganeshamoorthy, Saranya Mohandas, Joseph Robson, Aisha Sharif, Cameron Wolmarans, Diane Kay Bunn

**Affiliations:** 1 Norwich Medical School, University of East Anglia, Norwich, UK; 2 Health Innovation East, Cambridge, UK; 3 East of England Primary Care Deanery, Cambridge, UK; 4 Synthesis and Reviews Office, International Initiative for Impact Evaluation, New Delhi, India; 5 School of Health Sciences, University of East Anglia, Norwich, UK

**Keywords:** Primary Prevention, Systematic Review, Age Factors, Preventive Medicine, Aged, Meta-analysis, Prevalence

## Abstract

**Introduction:**

Low-intake dehydration is associated with higher mortality and poor health outcomes in older adults. Contributing factors (physiological, physical and cognitive decline, incontinence worries and reduced social drinking) may be exacerbated by illness, unfamiliar environment and routines in hospitalised older adults. We conducted a high-quality systematic review and meta-analysis to estimate dehydration prevalence amongst hospitalised adults (≥65 years) at admission.

**Methods:**

We included studies of hospitalised participants measuring serum osmolality or osmolarity. MEDLINE, Embase, Cochrane CENTRAL and CINAHL were searched (inception to July 2024). Inclusion, data extraction and risk of bias were assessed independently in duplicate. Data were described and synthesised in random effects meta-analysis (Meta-XL 5.3) and synthesis without meta-analysis (SWiM). Subgrouping assessed associations between dehydration prevalence and renal impairment, cognitive impairment, diabetic status, dependence, ethnicity, age, sex and economic status.

**Results:**

19 studies (of 13 097 assessed) reported the most reliable low-intake dehydration measures (directly measured osmolality >300 mOsm/kg, calculated osmolarity using Khajuria Krahn equation >300 mmol/L). 14 398 hospitalised participants were included of whom 5039 (35%) were dehydrated at or near admission. While meta-analysis suggested 23% (95% CI 17% to 30%, Grading of Recommendations, Assessment, Development and Evaluations (GRADE) moderate-quality evidence) were dehydrated, heterogeneity was high (I^2^ 98%) and different meta-analytic models suggested higher prevalence. Likely prevalence was a quarter to a third of hospitalised older adults. Subgrouping was generally not feasible, but SWiM suggested higher prevalence may be associated with impaired cognition, diabetes, renal impairment, and perhaps higher dependency. Limited evidence suggested dehydration prevalence reduced only slightly during hospitalisation.

**Conclusions:**

Between a quarter and a third of older adults have low-intake dehydration on hospital admission, varying with patient characteristics. Supporting older adults to drink well in hospital may be the appropriate response given high prevalence and severe health consequences. Trials are needed to assess effectiveness of in-hospital interventions to support drinking and improve health outcomes.

**PROSPERO registration number:**

CRD42021293763.

**WHAT IS ALREADY KNOWN**

- Low-intake dehydration (drinking too little) is associated with higher mortality and poor health outcomes in older people, but is poorly diagnosed.
- Dehydration and drinking, though crucial, are distinct from nutrition and eating, and remain under-researched.

**WHAT THIS STUDY ADDS**

- Low-intake dehydration is present in a quarter to a third of hospitalised older adults.
- Prevalence appears to reduce slightly during the hospital stay, but many who start dehydrated remain dehydrated.

**HOW THIS MAY AFFECT RESEARCH, PRACTICE OR POLICY**

- Given poor health outcomes associated with low-intake dehydration, and its high prevalence, we need to both:
  - Support all hospitalised older adults to drink well during their stay.
  - Work to identify low-intake dehydration in hospitalised older adults and ensure they become and remain hydrated.

## Introduction

 Hospitalised older adults may be at higher risk of low-intake dehydration than those living in the community due to illness and unfamiliar setting. A recent systematic review in community-dwelling older adults suggested a point prevalence of low-intake dehydration of 24% (95% CI 7% to 46%) across 61 studies. Those with more pre-existing illnesses had higher prevalence (37%, 95% CI 14% to 62%) than others (15%, 95% CI 0% to 43%), and prevalence was 19% (95% CI 0% to 48%) in the community but 34% (95% CI 9% to 61%) in long-term care residents.^1^

Low-intake dehydration is associated with poorer health outcomes. For example, El-Sharkawy and colleagues found that of 200 older adults with emergency admissions to a large UK hospital, the 37% dehydrated at admission were six times more likely to die in hospital than those not dehydrated (following careful adjustment for potential confounders, HR 6.0, 95% CI 1.6 to 22.3).^2^ Similar survival trends have been seen in other studies of older adults.^3^,^5^ Given this level of added risk for older adults with low-intake dehydration, if dehydration is also common in hospitalised older adults then resources to support drinking and hydration are likely to be cost-effective in terms of hospital costs, length of stay, mortality and morbidity.

Low-intake dehydration occurs when humans drink too little, resulting in a reduced volume of more concentrated body fluids, signalled by raised serum and plasma osmolality. Raised osmolality reflects increased concentrations of serum and plasma constituents, the gold standard physiological sign of low-intake dehydration.^6 7^ Fluid moves from within cells to the extracellular space, preserving circulation and blood pressure. This contrasts with extracellular dehydration caused by loss of both fluid and electrolytes through diarrhoea, vomiting or blood loss, which typically presents with hypotension rather than raised osmolality.^7^ Low-intake dehydration is clinically assessed in a variety of ways, but many signs and symptoms such as urine colour and volume, heart rate, dry mouth, feeling thirsty and bioelectrical impedance analysis are diagnostically inaccurate in older adults, being little more helpful than a random guess.^8^ Biochemical measures like plasma urea/creatinine ratio or urine specific gravity are also non-specific as raised levels may also be due to renal failure, heart failure, sarcopenia, glucocorticoids, bleeding or even high protein diets in older adults.^9^ Disparity between clinician diagnosis and the gold-standard biochemical assessment of serum osmolality (directly measured using freezing point depression) is common.^2 9 10^

We conducted a high-quality prevalence systematic review using the most robust measures of low-intake dehydration (serum or plasma osmolality or osmolarity) and best review methodology. We sought to answer the question: ‘What is the point prevalence of low-intake dehydration among adults aged ≥65 years in hospital settings?’ We analysed the range of prevalences in different groups (to help understand the variability in older adults with renal impairment, cognitive impairment, different levels of dependence, ethnicity and within countries of varied income levels) as well as pooled point-prevalence (to help understand the scale of the low-intake dehydration problem across hospital settings). We also explored how prevalence of dehydration altered during hospital stay.

## Methods

We based our systematic review methodology on JBI methods for systematic reviews of prevalence,^11 12^ which have been critiqued and broadened by the Prevalence Estimates Reviews – Systematic Review Methodology Group (PERSyst).^13 14^ Reporting was based on Preferred Reporting Items for Systematic Reviews and Meta-Analyses (PRISMA) guidance including a PRISMA Flowchart (there is no specific version of PRISMA for prevalence reviews).^15^ The objectives, inclusion criteria, searches and methods of analysis for this review were specified in advance and documented on PROSPERO^16^ (online supplemental file 12). This, and our sister review on prevalence in community dwelling older adults, started as one review and were split for practical reasons, so share search strategies and review processes.^1 17^ As this was a systematic review, ethical approval was not required. No members of the public or patients were directly involved in this systematic review. However, this topic was raised, and interest sparked and sustained by Patient and Public Involvement groups during our previous research in this area.^18 19^

### Searches

Relevant studies were identified using a structured search process^20^ developed using the PRESS Checklist.^21^ We searched MEDLINE (Ovid), Cochrane CENTRAL, Embase (Ovid) and CINAHL Complete from inception to 11 July 2024, plus Proquest Dissertations & Theses A&I to April 2023 and Nutrition & Food Sciences, to October 2021 (we were unable to update these searches due to changes in library access). Searches to April 2023 were shared with our sister systematic review.^1 17^ Reference lists of included studies and relevant systematic reviews were also searched.

The search process was as extensive as possible ensuring maximum reach and reducing the risk of publication bias. Electronic search strategies were complex, including text and indexing terms, truncation and Boolean operators. A full copy of the search strategies is in online supplemental file 1. Searches used the format:

[aged] and [prevalence or incidence] and [dehydration, osmolality or osmolarity or fluid] and [human]

### Inclusion criteria

Included studies reported data relating to the prevalence of low-intake dehydration in hospitalised older adults (≥65 years).

Participants: Adults with a mean age of ≥65 years, or where at least 80% of participants were aged ≥65. Participant groups chosen for factors that may influence dehydration prevalence (eg, groups chosen for having raised sodium, glucose or osmolality levels or being at the end of life) were excluded, as these populations would not be representative of broader groups of older adults. Participants whose fluid intake was being externally controlled, for example, by enteral or parenteral fluid provision, dialysis or pre- or peri-operative fluid regimens were also excluded unless baseline (pre-manipulation) data were presented.

Condition: Studies were included if prevalence of low-intake dehydration was assessed using any of the following methods:

1. Serum or plasma osmolality, directly measured using freezing point depression, defined as >300 mOsm/kg.^6^
2. Serum or plasma osmolarity calculated using the Khajuria Krahn equation, >300 mmol/L.^22 23^
3. Salivary osmolality, directly measured, >300 mOsm/kg.
4. Serum or plasma osmolarity calculated with any equation other than Khajuria Krahn, or where the equation was not defined, >300 mmol/L.^23^
5. Fluid intake, where current fluid intake was fully measured for at least 24 hours, and assessment methods and volumetric data reported, ≥2 L/day for men, ≥1.6 L/day for women.^6 24^

Methods 1–2 were considered at ‘low risk of bias’.

Context: Studies reporting hydration of older adults in hospital settings anywhere in the world were relevant. Hospitals included settings with a short term, residential and primarily medical purpose (excluding outpatients clinics, the community, primary care and long-term care). Hospital settings could include emergency departments (ED), general medical wards, rehabilitation units, geriatric or other specialist wards, intensive care units or any mixture.

Methodology: Both quantitative interventional and observational studies (encompassing cohort, cross-sectional, randomised and non-randomised controlled trials, and before-after studies) were included. These were not restricted by publication status, geography, language or date of publication. We excluded reviews, systematic reviews, qualitative research, case-control studies where cases were chosen by hydration status and studies with fewer than five participants. However, reference lists of relevant reviews, systematic reviews and included studies were retained and used to identify further studies.

### Assessment of inclusion

Titles and abstracts were imported into Covidence software (https://www.covidence.org/) and duplicates removed. Two reviewers independently screened all titles and abstracts using the inclusion criteria. Where a study appeared to meet all aspects of the inclusion criteria, or there was uncertainty about eligibility, the full text was retrieved. Inclusion of full text papers was assessed independently in duplicate, within Covidence. Discrepancies between reviewer judgements were discussed and where necessary arbitrated by another reviewer.

### Data extraction

Data were extracted within Covidence, independently in duplicate. Data extracted included bibliographic details, study characteristics, participant characteristics including those relevant to subgrouping, outcomes including dehydration prevalence and details of how hydration was assessed. When hydration was assessed multiple times, the earliest available data from the inpatient stay were used and presence of later data noted. We used studies with later (in-hospital) data to assess change in prevalence during hospital stay. Study protocols, further publications, registration, conference abstracts, supplementary data, errata and/or retraction statements were reviewed alongside the main published papers. We attempted to contact study authors where additional information was required to assess inclusion or for missing information. Discrepancies between data extraction were reviewed, discussed, agreed within Covidence and arbitrated by a third reviewer where needed.

### Risk of bias assessment

Risk of bias for each included study was assessed by two independent reviewers and differences discussed, then reviewed by a third reviewer for consistency (DKB). Risk of bias assessment was based on an adapted version of the JBI ‘Checklist for prevalence studies’.^1 12^

### Data management

We analysed data by method of assessment of low-intake dehydration using the highest quality data from each included study (according to the hierarchy above). Prevalence data (number of dehydrated participants given our specified cut-offs and total number of participants) were converted to proportions. For studies providing mean and variance of osmolality or osmolarity, the total number dehydrated (over the relevant cut-off) was calculated assuming data were normally distributed.

### Strategy for data synthesis

To address the main review question ‘What is the prevalence of low-intake dehydration among adults aged ≥65 years in hospital settings?’, we tabulated prevalence data study-by-study including number of participants, number dehydrated and quality assessment and then grouped by method of assessment, including mean and 95% CI of the group of prevalences, the median and IQR of the group of prevalences and the range of prevalences.

We planned to use meta-analysis to combine data from studies assessing directly measured serum or plasma osmolality and calculated serum or plasma osmolarity (using the Khajuria Krahn equation, which is validated in a wide range of older adults, including those with diabetes and renal problems^22^) as these two dehydration measures are highly comparable,^23 25^ and analysis of a larger group of studies allows use of a larger dataset, offering more power and greater ability to assess subgroup differences. Where studies provided both directly measured osmolality and calculated Khajuria Krahn osmolarity data, we used only directly measured serum or plasma osmolality data in meta-analysis. We assessed heterogeneity between the two sets of studies and planned to separate these two groups if they appeared highly heterogeneous, assessed by checking whether combining both data sets in random effects meta-analysis increased I^2^.

Reasoning behind our main meta-analytic methods and sensitivity analyses is in online supplemental file 2. For each group, we planned to run:

1. Meta-analyses in Meta-XL 5.3^26 27^ using double arcsine transformation, random effects meta-analysis (DerSimonian and Laird), heterogeneity described using I^2^ and Doi plots to describe the potential for publication or small study bias.
2. Sensitivity analyses for the directly measured and calculated Khajuria Krahn data (together or separately as above):
  - Logit transformation.
  - Quality effects meta-analysis.
  - Inverse variance heterogeneity meta-analysis.
  - Random effects (double arcsine transformation) omitting single outlying studies.
3. Subgroup analysis.
4. To answer the sub-questions we planned to, where the data were comparable, subgroup prevalence of low-intake dehydration using directly measured osmolality or Khajuria Krahn calculated osmolarity where there were at least 10 studies by:
  - Renal impairment (participants with or without renal impairment, defined as in the original papers).
  - Cognitive impairment (participants with or without cognitive impairment).
  - Diabetic status (participants with or without diabetes diagnosis).
  - Economic status of the countries where the research was carried out: (i) high income, (ii) upper-middle income, (iii) lower-middle income and (iv) low income.^28^
  - Levels of dependency in accessing drinks and drinking, grouped by (i) functionally independent, (ii) semi-independent, (iii) total dependence on others, (iv) mixed dependency and (v) unclear dependency level.
  - Ethnicity of participants.

Where data were not comparable for meta-analysis, we planned to discuss the evidence narratively (using tables where helpful) based on synthesis without meta-analysis (SWiM) guidelines.^29^ We decided post-hoc to also subgroup by age, gender, number of comorbidities and types of patients (geriatric unit, ED or acute care, post-stroke or planned admission) as observational studies have suggested that greater age, male gender, reason for admission and poorer baseline health may increase low-intake dehydration risk in older adults. We also decided post-hoc to follow hydration prevalence data through the hospital stay and combine results narratively. We attempted Grading of Recommendations, Assessment, Development and Evaluations (GRADE) assessment of the evidence on prevalence but did not produce a formal summary of findings table.

## Results

**Figure 1:**
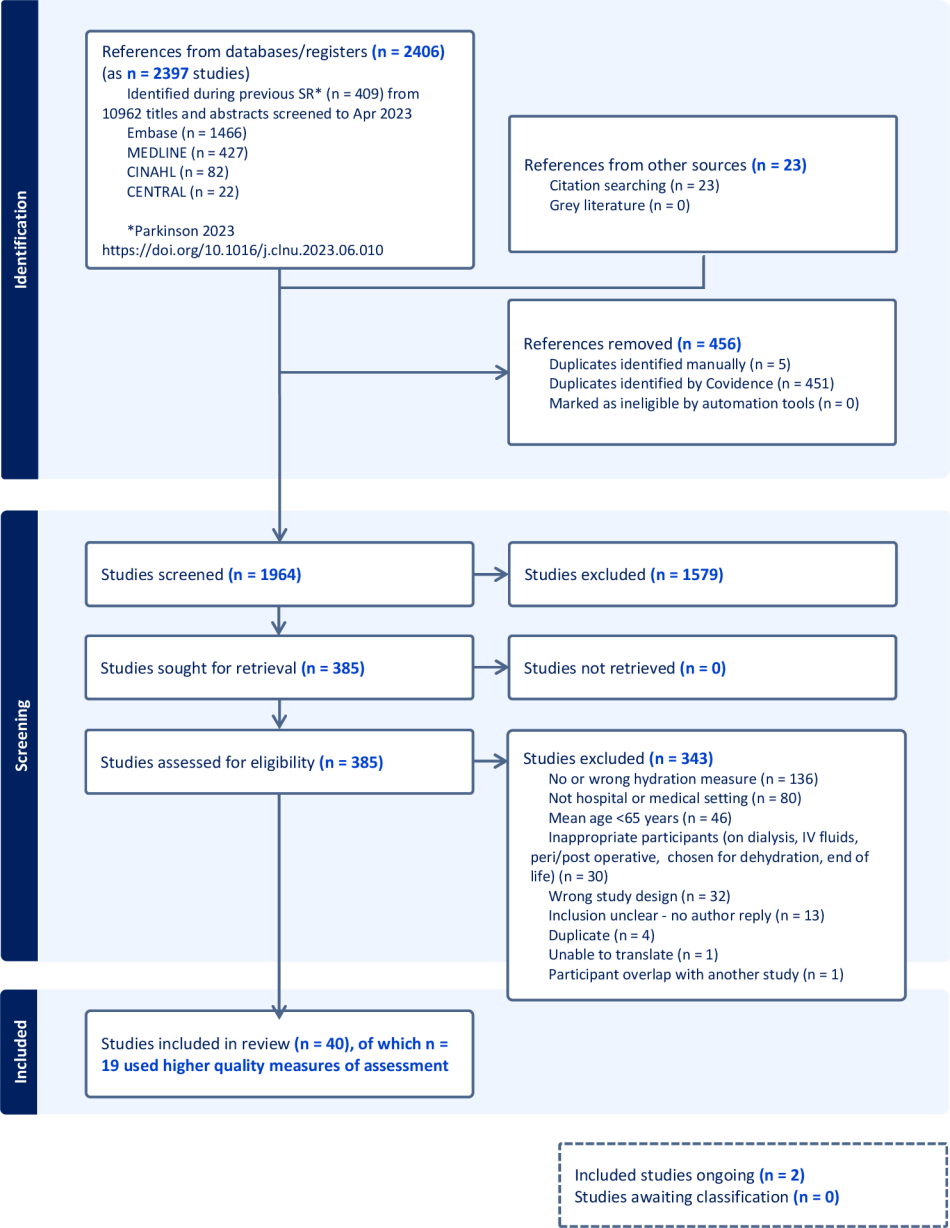
PRISMA flow chart. PRISMA, Preferred Reporting Items for Systematic Reviews and Meta-Analyses. IV intravenous, SR systematic review.

We imported 409 potentially relevant hospital studies first identified in our previous review for reassessment.^1^ Those searches, to April 2023, included 10 962 titles and abstracts identified from ProQuest dissertations and Nutrition and Food Sciences to March 2021, and CINAHL, Cochrane CENTRAL, Medline and Embase plus 37 from hand searching and 78 identified from reference lists of included studies.^1^ We ran updated searches in Medline (Ovid), Embase (Ovid), CENTRAL (Cochrane systematic reviews, protocols and trials) and CINAHL to 11 July 2024, for this review identifying a further 1997 titles and abstracts (2397 studies when new titles and abstracts were combined with the 409 previously identified publications and different publications from the same study were combined). A further 23 possible studies were identified from citation searching. Between the two review search processes we assessed 13 097 titles and abstracts for this review. We assessed 385 studies in full text, excluding 343 as ineligible (see figure 1), including 40 studies with hydration data and two ongoing studies.^30 31^ For the list of studies assessed in full text but excluded, see.

Characteristics of the 40 included studies with outcome data are tabulated and summarised in). Of the 40 included studies:

- 12 assessed directly measured osmolality in mOsm/kg (assessed by freezing point depression).^23 10 25 32^,^39^
- 15 assessed calculated osmolarity using the Khajuria Krahn equation,^525 35^,^37 40^ but four did not provide enough information to assess number dehydrated at the >300 mMol/L cut-off^41 45 46 48^ and four also provided directly measured osmolality which was used in preference to calculated data,^2535^,^37^ so seven were used in combined analyses.^540 42^,^44 47 49^
- 11 studies assessed calculated osmolarity using equations other than Khajuria Krahn,^450^,^59^ of which four did not provide data for meta-analysis.^452^,^54^ Five assessed osmolarity or osmolality but the exact method used was not clear and we received no reply from our author enquiries.^60^,^64^ Of these 16 studies (non-Khajuria Krahn equation or unclear), 10 could be combined in meta-analysis.^5051 55 56 58^,^62 64^
- Two studies assessed fluid intake fairly rigorously,^64 65^ but neither provided intake by sex (necessary to assess adequate intake).
- One study assessed salivary osmolality.^34^

The characteristics of the subset of 19 studies with the best quality data that contributed to the main meta-analysis (those that used either directly measured osmolality or calculated osmolarity using the Khajuria Krahn equation and provided hydration data above 300mOsm/kg or 300 mmol/L cut-offs) are described in table 1 (and online supplemental files 4–6). Mean ages ranged from 65 to 85 years (plus one with a median age of 86). The number of participants ranged from 27 to 6632, and percentages of women participants from 17% to 65%. Participants included patients diagnosed with stroke, decompensated liver cirrhosis, cognitive decline or requiring cardiac surgery. Others represented mixed groups of older adults in the ED, geriatric ward or medical wards. 16 were conducted in Europe, two in Asia, one in Australia. Study designs were mixed, including one randomised controlled trial, 10 cohort studies, one case control (where case selection was not based on hydration status), six cross-sectional studies and one diagnostic accuracy study.

**Table 1:** Brief table of characteristics of the 19 studies included in the meta-analysis

Study ID, continent of study	Study design	Study dates	Population description	N with data on hydration status	Age, mean (SD) in years*	Female (n, %)
Bech,^40^ Europe	Cohort	May to Nov 2022	Adults aged 65+ on geriatrics unit	114	Median 85.5, IQR 80 to 89	65, 57%
Bhalla,^3^ Europe	Cohort	Nov 1998 to Nov 1999	Patients with diagnosed stroke	167	73 (12)	87, 52%
Buaprasert,^32^ Asia	Cross-sectional	May to Jul 2017	Adults aged 65+ in ED	370	78 (8)	234, 63%
Claesson-Lingehall,^33^ Europe	RCT	Apr 2019 to Jun 2020	Patients 65+ scheduled for cardiac surgery	195	73 (4)	53, 27%
El-Sharkawy,^2^ Europe	Cohort	Aug 2012 to Apr 2014	Adults aged 65+ with emergency admission	187	82 (7)	87, 47%
El-Sharkawy,^5^ Europe	Cohort	May 2011 to Oct 2013	Patients aged 65+ with emergency admission to medical specialities	6632	NR	3469, 52%
Fortes,^34^ Europe	Cross-sectional	May to Nov 2011	Adults aged 60+ admitted to acute medical care or ED	130	78 (9)	71, 55%
Kafri,^35^ Europe	Cross-sectional	Apr 2011 to Oct 2011	Patients with stroke admitted within 48 hours of symptom onset	27	71 (11)	11, 41%
Lauriola,^42^ Europe	Case control	Jan 2015 to Mar 2017	Adults with or without cognitive decline, geriatric unit	1091 (571 cases, 520 controls)	79 (7)	549, 50%
Miller,^43^ Europe	Cohort	Mar to June 2017	Adult patients admitted with stroke	41 of 48	Mean 75, range 48–100	25, 50%
Munk,^25^ Europe	Diagnostic	Apr to May 2019	Patients aged 65+ years admitted to ED	90	Median 78, IQR 72 to 86	48, 53%
Nagae,^44^ Asia	Cohort	Oct 2019 to Mar 2022	Patients aged 65+ years admitted to geriatric ward	192	85 (6)	113, 59%
Pfortmueller,^36^ Europe	Cross-sectional	Jan 2002 to Dec 2012	Patients 65+ with decompensated liver cirrhosis in ED	54	69 (4)	13, 24%
Sanson,^47^ Europe	Cohort	Oct 2015 to Jul 2016	Patients 65+ admitted from ED to Internal Medicine	4613	NR	2675, 58%
Sjostrand,^37^ Europe	Cohort	Spring to summer 2010	Patients 75+ admitted to ED awaiting treatment	36	84 (6)	20, 56%
Van Wijk,^38^ Europe	Cross-sectional	Jan 2018 to Jul 2019	Patients with ischaemic stroke 50–75 years, some with oropharyngeal dysphagia	36	65 (7)	6, 17%
Vivanti,^10^ Anzac	Cohort	May to Dec 2002	Older adults voluntarily admitted to geriatric rehab	43	78 (8)	28, 65%
Walsh,^39^ Europe	Cross-sectional	Jul to Dec 2011	Older hospital patients on acute medical unit	99	77 (8)	55, 50%
Zanetti,^49^ Europe	Cohort	Jan to Dec 2019	Older adults acutely admitted to geriatric unit	529	85 (7)	326, 62%

* Data reported as mean (SD) unless otherwise stated.

ED, emergency department; NR, Not reported; RCT, randomised controlled trial.

Risk of bias of all 40 included studies is shown and summarised in online supplemental file 7. The 19 studies that contributed to the meta-analysis all assessed dehydration in a reliable way (by definition). Recruitment was clearly appropriate in providing a representative sample of hospitalised older adults in 10 studies (53%); participants and setting were well-described in 10 studies (53%); sampling frame focused on older adults in 16 included studies (84%): data analysis had sufficient coverage of the sample in 15 studies (79%); directly reported the number of dehydrated participants (using our cut-offs) and the sample size in 11 studies (58%); response rate was judged adequate in seven studies (37%); and no further issues were identified in 16 studies (84%).

**Figure 2:**
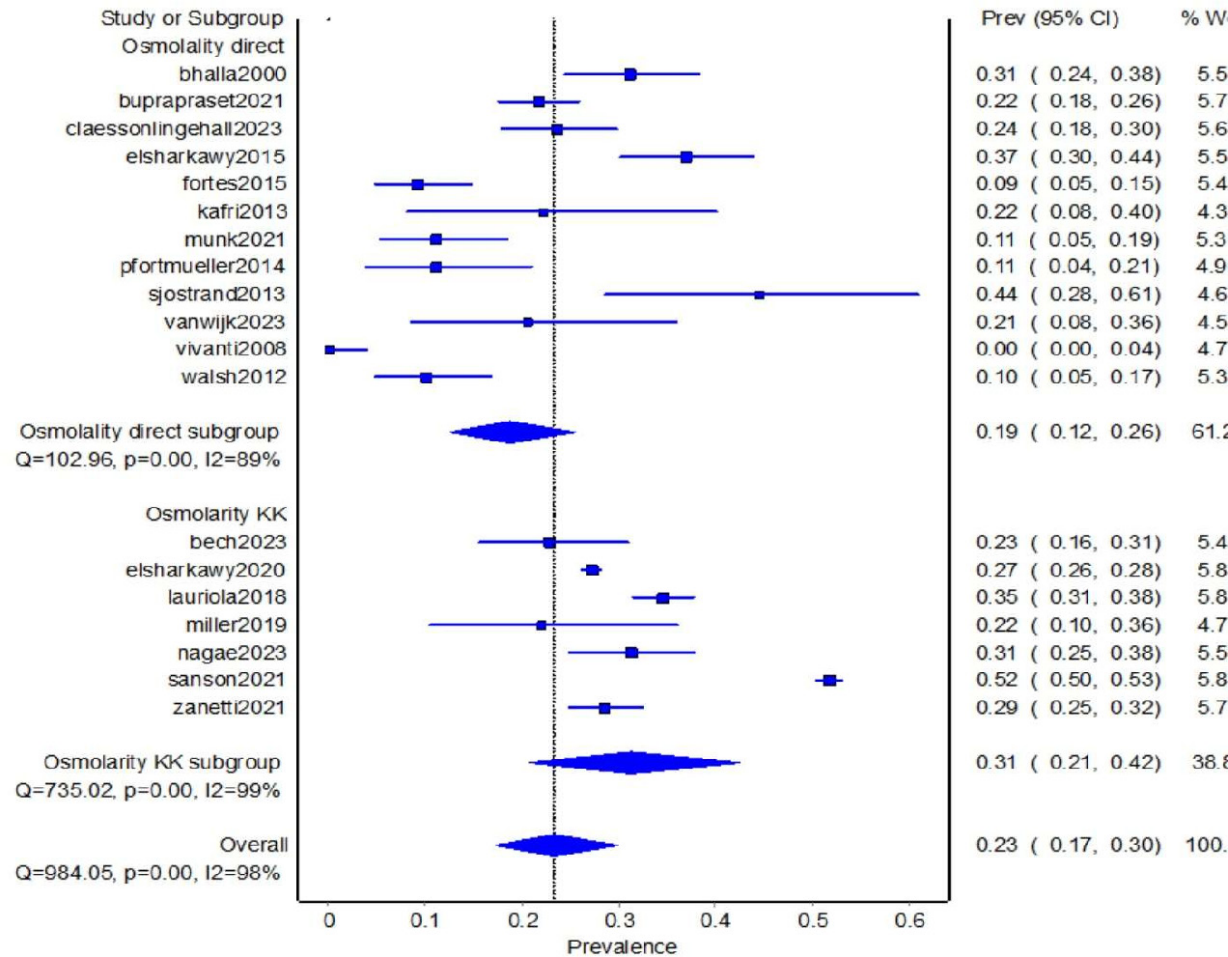
Random effects meta-analysis with double arcsine transformation of directly measured osmolality and calculated osmolarity (using the Khajuria Krahn (KK) equation), sub-grouped by directly measured or calculated data.

Random effects meta-analysis (double arcsine transformed) subgrouping by directly measured osmolality or calculated osmolarity (Khajuria Krahn equation) data suggested high I^2^ in all groups (high heterogeneity is typical for prevalence data). I^2^ was 89% in the directly measured osmolality subgroup, 99% in the calculated osmolarity subgroup, and 98% overall (see figure 2). The combined I^2^ was higher than that of the directly measured subgroup and slightly lower than that of the calculated subgroup. We interpreted this as suggesting it was appropriate to analyse these two sets of data together.

Combining directly measured osmolality and calculated osmolarity (Khajuria Krahn equation) 19 studies were included, representing 14 398 hospitalised older adults, of whom 5039 (35%) had low-intake dehydration on a single assessment (see table 2). The low-intake dehydration point-prevalence range was wide, from 1% to 52%, mean 24% (95% CI 0% to 49%) and median 23% (IQR 11% to 31%). Random effects meta-analysis (with double arcsine transformation) suggested prevalence of 23% (95% CI 17% to 30%, I^2^ 98%). The Doi plot showed major asymmetry and of 23 studies providing directly measured osmolality or appropriately calculated osmolarity data only 19 provided enough information to be included in the meta-analysis, so data are missing on an additional 713 older adults in these studies.

**Table 2:** Prevalence of low-intake dehydration in older hospitalised adults by dehydration assessment method, of studies with data on dehydration prevalence useable in meta-analysis

Characteristic	Combination of directly measured and calculated with KK osmolality/ osmolarity	Directly measured serum/plasma osmolality >300 mOsm/kg	Calculated serum or plasma osmolarity >300 mmol/L using KK	Calculated serum or plasma osmolarity using other equations (>300 mmol/L) or unclear
Number of studies	19	12	11	10
Total number of participants	14 398	1432	13 137	25 158
Total number dehydrated	5039 (35%)	314 (22%)	4781 (36%)	14 985 (60%)
Range dehydrated by study	1% to 52%	1% to 44%	12% to 58%	7% to 82%
Mean (95% CI) dehydrated	24% (0% to 49%)	20% (0% to 46%)	32% (4% to 60%)	46% (0% to 96%)
Median (IQR) dehydrated	23% (11% to 31%)	20.5% (10.5% to 27.5%)	29% (22% to 44%)	47.5% (22.5% to 67.5%)
RE MA, double arcsine	23% (17% to 30%), I^2^ 98%, major asymmetry	19% (12% to 26%), I^2^ 89%	31% (23% to 41%), I^2^ 99%, no asymmetry	49% (44% to 54%), I^2^ 95%, major asymmetry

Arcsin, data transformed using double arcsine; Asym, asymmetry according to the Doi plot^70^; IV, inverse variance (fixed effects); IVhet, Inverse variance heterogeneity^71^; KK, Khajuria Krahn equation^22^; Logit, data transformed using logit; MA, meta-analysis; QE, Quality effects^72^; RE, random effects (DerSimonian and Laird).

When assessing directly measured serum or plasma osmolality only, we included 12 studies representing 1432 participants of whom 314 (22%) were dehydrated, range 1 to 44% dehydrated by study, mean of studies 20% (95% CI 0% to 46%) dehydrated. Meta-analysis suggested that 19% were dehydrated (95% CI 12% to 26%, I^2^ 89%, random effects meta-analysis using double arcsine transformation). When only assessing the 11 studies that provided data on calculated serum or plasma osmolarity using the Khajuria Krahn equation, the numbers of participants were higher (as many were retrospective cohorts) with 13 137 participants, of whom 36% were dehydrated, with 12 to 58% of participants dehydrated by study. The pooled prevalence was 31% (95% CI 23% to 41%, I^2^ 99% without asymmetry in the Doi plot). The 10 studies of calculated osmolarity using other or unclear equations included over 25 000 participants of whom 60% were dehydrated (range 7–82% of participants by study, pooled data 49% (44% to 54%, random effects meta-analysis using double arcsine transformation), I^2^ 95%.

Sensitivity analyses of the 19 studies providing directly measured and calculated osmolarity (Khajuria Krahn equation only) suggested pooled prevalence generally higher than the 23% suggested in the main analysis, from 22% to 35% (online supplemental file 8). Major asymmetry and very high heterogeneity remained for all sensitivity analyses apart from that omitting the biggest outlier (Sanson 2021^47^ where the Doi plot suggested minor asymmetry and I^2^ dropped from 98% in the main analysis to 89%. Sanson 2021 had the highest prevalence of dehydration, at 52% (50 to 53%). Sanson 2021 conducted a retrospective assessment of calculated osmolarity using the Khajuria Krahn equation in 5113 older patients consecutively acutely admitted to an Italian Internal Medicine department. It was unclear whether the components of osmolality were collected at the same time, and at which point during the inpatient stay this may have been.^47^

We aimed to use subgrouping to understand which groups of older adults were more likely to be dehydrated. Information on the characteristics of included studies related to subgrouping by potential causal factors are in online supplemental file 4. The associations (noted below) were generally univariate analyses, and confounders not adjusted for. We defined a positive association to be when being healthier was associated with lower levels of low-intake dehydration (or worse health/well-being associated with higher risk of dehydration). As meta-analytic subgrouping was often not possible, we carried out SWiM analysis using vote-counting of studies assessing the relationship between hydration and the measure of interest. As studies were often small, we assessed the direction of associations even in the absence of statistically significant effects. This method was also used for other subgrouping components. The results are summarised in table 3 (details in online supplemental file 9 and subgrouping in online supplemental files 10 and 11).

**Table 3:** SWiM analysis of factors associated with higher risk of dehydration in older hospitalised adults.

Criterion	Studies with data	Positive association (statistically significant)	Positive association (NOT statistically significant)	No or unclear association	Negative association (NOT statistically significant)	Negative association (statistically significant)	Suggestion
Dependency	8	3	2	1	2	0	Modest suggestion that greater dependency is associated with poorer hydration
Cognition	5	3^5 42 44^	2^2 32^	0	0	0	Suggestion that poorer cognition is associated with poorer hydration
Diabetic status	7	3^3 10 40^	2^32 44^	2^25 35^	0	0	Suggestion that being diabetic is associated with poorer hydration
Renal impairment	7	6^2 10 32 40 44 47^	0	1^25^	0	0	Suggestion that renal impairment is associated with poorer hydration
Economic status	19						Not assessable
Age	10	1^3^	5^5 10 32 34 44^	4^25 35 40 42^	1^2^	0	Modest suggestion that greater age is associated with poorer hydration, but no suggestion of this in subgrouping (online supplemental file 10)
Gender	7	3 (men at greater risk)^5 25 40^	0	1^32^	3^2 10 44^	0 (women at greater risk)	Unclear
Ethnicity	4						Not assessable
Comorbidities	3	2^32 44^	0	1^2^	0	0	Not assessable
Settings	0						Subgrouping only (online supplemental file 11), no clear association of setting and hydration risk

For description of the findings of the studies, see online supplemental file 9; for subgroup analyses, see online supplemental files 10 and 11.

SWiM, synthesis without meta-analysis.

Overall, SWiM analysis suggested that poorer cognition, being diabetic and having renal impairment are likely to be associated with greater risk of dehydration, while dependent status may also be. Risk did not appear to differ markedly by age (in these already older patients) or hospital setting in subgrouping. Associations with broader economic status, ethnicity and number of comorbidities were not assessable.

### Change in prevalence during hospital stay

We looked for evidence of changes in low-intake dehydration prevalence during the hospital stay, to help in understanding how well hospitals are hydrating older patients with or without dehydration at admission. Only 5 of the 40 studies assessed dehydration after baseline, and only two of these used an accurate measure, directly measured plasma osmolality.^2 3^ These are briefly discussed here.

Bhalla *et al*^3^ assessed directly measured plasma osmolality at baseline and day 7 in 167 patients with acute stroke. Of the 70 taking only oral fluids mean osmolality dropped slightly from 292.4 (SD 8) to 291.6 (SD 8) mOsm/kg at day 7. Of the 47 only taking intravenous fluids mean osmolality was higher and didn’t fall (299.1 (SD 11) to 299.4 (SD 17) mOsm/kg). In the 39 on a mixture of oral and intravenous fluids mean osmolality fell most from 294.7 (SD 9) at baseline to 288.7 (SD 26) mOsm/kg at day 7.

El-Sharkawy *et al*^2^ provided data on directly measured osmolality at baseline and 48 hours, finding that of 200 older adults acutely admitted to a UK hospital 37% were dehydrated at baseline, and of these 62% were still dehydrated at 48 hours (those not dehydrated at baseline were not assessed at 48 hours so how their hydration status changed in hospital is not clear). They noted that dehydration may have remained high as only 8% of participants were clinically diagnosed with dehydration in medical notes.

The three studies using less reliable hydration measures were less consistent in finding small reductions in dehydration during the hospital stay. Khanimov *et al*^55^ reported changes in calculated osmolarity (equation not given) between baseline and the time of lowest documented albumin. Exact numbers were not provided but there was little change in osmolality in group 1 (albumin levels started and remained >3.5 g/dL, mean length of stay 3 days, 3399 participants) or 2 (albumin levels started <3.5 g/dL and remained at or above these levels, mean length of stay 5 days, 2762 participants), but a significant rise of around 2 mOsm/L in group 3 (any admission serum albumin level but albumin fell by more than 0.1 g/dL at some point during their hospital stay, mean duration 5 days, 1557 participants).

McCrow *et al*^56^ reported calculated osmolarity (equation not given) at baseline and day 4 in 44 participants with and without cognitive impairment. They found that 12 were dehydrated by their definition at admission, but the study provided conflicting information about day 4.

Oh and Seo^64^ provided data on ‘osmolality’ (unclear whether directly measured or calculated) before tube feeding was initiated (baseline data) and then daily to 3 days in their retrospective assessment of 85 patients with acute brain infarction. They found that with protocol led tube feeding osmolality fell from mean 306.1 mOsm (SD 41) at baseline to 297.3 (SD 18) at day 3.

Overall, the data suggest that mean osmolality reduces (hydration is improved) somewhat in older adults during hospital stays, though this varies from hospital to hospital and fluid intake methods, but many older adults remain dehydrated during their hospital stay.

### GRADE quality of evidence

We did not carry out a formal GRADE assessment but characterised the evidence of prevalence of low-intake dehydration in hospitalised older adults from the 19 studies with best hydration assessment according to the GRADE elements, starting with high quality evidence. This suggested that we downgrade once for both inconsistency (heterogeneity was high but partly explained by subgrouping characteristics) and publication bias (as Doi plots suggested major asymmetry, though as most studies were not consciously assessing hydration prevalence this is less likely), resulting in moderate-quality evidence that the prevalence of low-intake dehydration in hospitalised older adults was around 23% (95% CI 17% to 30%). However, this prevalence clearly varied according to individual characteristics of older adults and may also vary culturally from hospital to hospital and country to country.

## Discussion

### Statement of principal findings

We included 40 studies on low-intake dehydration in older adults, of which 19 provided high quality hydration assessment. These 19 studies included 14 398 participants, of whom 5039 (35%) were dehydrated. 16 of the 19 studies were conducted in Europe, and participants represented mixed groups of older adults in the ED, geriatric ward or medical wards or were diagnosed with stroke, cirrhosis, cognitive decline or requiring cardiac surgery. Individual studies reported 1% to 52% dehydrated participants. The main meta-analysis suggested 23% (95% CI 17% to 30%, GRADE moderate-quality evidence) were dehydrated, but heterogeneity was high (I^2^ 98%) and sensitivity analyses suggested 22%–35% were dehydrated (with 95% CIs running from 17% to 52%). As the pooled prevalence depended on meta-analytic methods, and because of very high heterogeneity in every analysis, the best way to describe the prevalence of dehydration in older hospitalised adults may be that a quarter to a third are likely to be dehydrated.

SWiM vote-counting suggested that poorer cognition, being diabetic and renal impairment are likely to be associated with greater risk of dehydration (higher levels of dependence may also be). Risk did not clearly differ by age (between younger and older elderly) or hospital setting in subgrouping. Limited evidence suggested that hydration was improved somewhat in older adults during their hospital stay, but many older adults remained dehydrated.

### Strengths and weaknesses of the systematic review

Strengths of this review included an extensive and complex search strategy, protocol publication, independent duplication of all major steps, risk of bias assessment based on JBI guidelines for prevalence reviews and careful analysis. However, included studies often did not describe their assessment measure, and many otherwise eligible studies used methods of assessment not specific to dehydration in older adults (like urea to creatinine ratio) or poorly diagnostic (such as skin turgor, urine colour, physician assessment or osmolarity equations not equating to directly measured osmolality).^6 8 23^ When directly measured osmolality is assessed it may not be mentioned in study abstracts making these studies difficult to identify with even complex searches, so we are likely to have missed some studies. In some cases, studies did not describe the equation they used to calculate osmolarity when discussing dehydration, or the setting, and some did not provide osmolality or osmolarity data in a way that allowed us to calculate prevalence at a useful cut-off (>300 mOsm/kg or mmol/L). Further, retrospective studies often did not specify that all serum measures used to calculate osmolarity had been taken from the same blood draw (which may affect accuracy adversely).

There is no definitive methodological guidebook for conducting systematic reviews of prevalence studies. We chose the strongest methods available^11^,^14^ and ran extensive sensitivity analyses, which suggested that the meta-analytic model chosen affects the prevalence estimate, making any single estimate less reliable. High levels of heterogeneity were present regardless of the analytical method chosen, which is common in prevalence reviews, and likely represents underlying variation in dehydration risk in different populations. Such high levels of heterogeneity are common in meta-analysis of prevalence and may be due to the nature of non-comparative proportional data.^66^ This heterogeneity does suggest that dehydration is associated with specific environmental, cultural or health factors, not simply due to ageing, and so is likely to be preventable.^1^ Promoting factors are likely to include diabetic status, cognitive function, renal impairment and possibly some types of dependence. Local social and cultural factors related to drinking, as well as the hospital culture surrounding drinking, may play a role but are difficult to assess in a systematic review.

Publication bias is also a potential cause for concern, but best methods to assess it, or manage it, in prevalence reviews remain unclear. The experimental Doi plot suggests that publication bias may be present in our analysis, and we are aware of four studies (713 participants) that could not be included in meta-analysis as data were not clearly enough presented. Other relevant studies may have been missed as they did not mention osmolarity, osmolality or fluid intake in the abstract and these measures were not noted in assigning indexing terms.

### Strengths and weaknesses in relation to other studies

The suggestion that a quarter to a third of hospitalised older adults have low-intake dehydration echoes estimates in community dwelling older adults. Parkinson *et al*^1^ systematically reviewed the prevalence of low-intake dehydration using the same robust measures. They estimated 15% (95% CI 0% to 43%) of older adults living in the community, and 34% (95% CI 9% to 61%) in long-term care were dehydrated, and those with more comorbidities had a higher prevalence (37%, 95% CI 14% to 62%) than those with fewer. An earlier systematic review using measures that lack diagnostic accuracy in older adults (tongue furrows, urea to creatinine ratio, hypernatraemia, skin turgor, physician assessment and undefined methods as well as serum osmolality)^8^ found a prevalence of low-intake dehydration in older adults in long-term care of 1–39%.^67^ We have not identified other systematic reviews of the prevalence of low-intake dehydration in hospitalised older adults.

### Meaning of the study

A quarter to a third of older hospitalised adults have low-intake dehydration, associated with poorer outcomes, longer hospital stays and increased risk of mortality. While those at greatest risk may include those with diabetes, poorer cognition or renal function and perhaps higher levels of dependency, these factors do not explain all the variation in prevalence between studies. Prioritising adequate fluid intake for all older hospital patients may be the most effective approach. Hospitals rightly focus on feeding hospital patients to support good nutrition, but a greater focus on drinking and hydration is needed as well, as factors supporting good hydration appear distinct from those supporting good nutrition.^40 68 69^

### Unanswered questions and future research

Research is needed to assess the best ways to support adequate drinking in hospital, and to accurately assess the effects of interventions to support hydration in older adults on hydration status, using robust measures of low-intake dehydration, and most importantly on health outcomes.

## Supplementary material

10.1136/bmjph-2025-002985 online supplemental file 1

10.1136/bmjph-2025-002985 online supplemental file 2

## Data Availability

All data relevant to the study are included in the article or uploaded as supplementary information.

## References

[1] Parkinson E, Hooper L, Fynn J (2023). Low-intake dehydration prevalence in non-hospitalised older adults: Systematic review and meta-analysis. Clin Nutr.

[2] El-Sharkawy A, Watson P, Neal KR (2015). Hydration and outcome in older patients admitted to hospital (The HOOP prospective cohort study). Age and Ageing.

[3] Bhalla A, Sankaralingam S, Dundas R (2000). Influence of Raised Plasma Osmolality on Clinical Outcome After Acute Stroke. Stroke.

[4] Bourdel-Marchasson I, Proux S, Dehail P (2004). One-year incidence of hyperosmolar states and prognosis in a geriatric acute care unit. Gerontology.

[5] El-Sharkawy AM, Devonald MAJ, Humes DJ (2020). Hyperosmolar dehydration: A predictor of kidney injury and outcome in hospitalised older adults. Clin Nutr.

[6] Volkert D, Beck AM, Cederholm T (2022). ESPEN practical guideline: Clinical nutrition and hydration in geriatrics. Clin Nutr.

[7] Lacey J, Corbett J, Forni L (2019). A multidisciplinary consensus on dehydration: definitions, diagnostic methods and clinical implications. Ann Med.

[8] Hooper L, Abdelhamid A, Attreed NJ (2015). Clinical symptoms, signs and tests for identification of impending and current water-loss dehydration in older people. Cochrane Database Syst Rev.

[9] Thomas DR, Cote TR, Lawhorne L (2008). Understanding Clinical Dehydration and Its Treatment. J Am Med Dir Assoc.

[10] Vivanti A, Harvey K, Ash S (2008). Clinical assessment of dehydration in older people admitted to hospital: what are the strongest indicators?. Arch Gerontol Geriatr.

[11] Munn Z, Moola S, Lisy K Chapter 5: Systematic reviews of prevalence and incidence.

[12] Munn Z, Moola S, Lisy K (2015). Methodological guidance for systematic reviews of observational epidemiological studies reporting prevalence and cumulative incidence data. Int J Evid Based Healthc.

[13] Borges Migliavaca C, Stein C, Colpani V (2020). on behalf of the Prevalence Estimates Reviews – Systematic Review Methodology G. How are systematic reviews of prevalence conducted? A methodological study. BMC Med Res Methodol.

[14] Barker TH, Migliavaca CB, Stein C (2021). Conducting proportional meta-analysis in different types of systematic reviews: a guide for synthesisers of evidence. BMC Med Res Methodol.

[15] Page MJ, McKenzie JE, Bossuyt PM (2021). The PRISMA 2020 statement: an updated guideline for reporting systematic reviews. BMJ.

[16] Hooper L, Bunn D, Edozie O (2021). Prevalence of low-intake dehydration among older adults in hospital: a systematic review and meta-analysis: CRD42021293763.

[17] Parkinson E, Hooper L, Poland F (2021). Prevalence of dehydration among older adults across settings: a systematic review and meta-analysis: CRD42021241252.

[18] Jimoh OF, Brown TJ, Bunn D (2019). Beverage Intake and Drinking Patterns-Clues to Support Older People Living in Long-Term Care to Drink Well: DRIE and FISE Studies. Nutrients.

[19] Bunn DK, Hooper L (2019). Signs and Symptoms of Low-Intake Dehydration Do Not Work in Older Care Home Residents-DRIE Diagnostic Accuracy Study. J Am Med Dir Assoc.

[20] Lefebvre C, Glanville J, Briscoe S (2024). Cochrane handbook for systematic reviews of interventions version 65.

[21] McGowan J, Sampson M, Salzwedel DM (2016). PRESS Peer Review of Electronic Search Strategies: 2015 Guideline Statement. J Clin Epidemiol.

[22] Khajuria A, Krahn J (2005). Osmolality revisited--deriving and validating the best formula for calculated osmolality. Clin Biochem.

[23] Hooper L, Abdelhamid A, Ali A (2015). Diagnostic accuracy of calculated serum osmolarity to predict dehydration in older people: adding value to pathology laboratory reports. BMJ Open.

[24] EFSA Panel on Dietetic Products, Nutrition, and Allergies (NDA) (2010). Scientific Opinion on Dietary Reference Values for water. EFS2.

[25] Munk T, Bech CB, Klausen TW (2021). Accuracy of the calculated serum osmolarity to screen for hyperosmolar dehydration in older hospitalised medical patients. Clin Nutr ESPEN.

[26] Barendregt JJ, Doi SA (2016). MetaXL version 5.3.

[27] Barendregt JJ, Doi SA (2016). MetaXL User Guide, Version 5.3.

[28] The World Bank (2024). World Bank Country and Lending Groups: Country Classification.

[29] Campbell M, McKenzie JE, Sowden A (2020). Synthesis without meta-analysis (SWiM) in systematic reviews: reporting guideline. BMJ.

[30] Atkins KJ, Scott DA, Silbert B (2021). Preventing Delirium and Promoting Long-Term Brain Health: A Clinical Trial Design for the Perioperative Cognitive Enhancement (PROTECT) Trial. *J Alzheimers Dis*.

[31] Seymour DG, Henschke PJ, Cape RD (1980). Acute confusional states and dementia in the elderly: the role of dehydration/volume depletion, physical illness and age. Age Ageing.

[32] Buaprasert P, Piyapaisarn S, Vanichkulbodee A (2021). Prevalence and risk factors of hypertonic dehydration among older patients admitted to the emergency department: A prospective cross-sectional study. Geriatr Gerontol Int.

[33] Claesson Lingehall H, Gustafson Y, Svenmarker S (2023). Is a hyperosmolar pump prime for cardiopulmonary bypass a risk factor for postoperative delirium? A double blinded randomised controlled trial. Scand Cardiovasc J.

[34] Fortes MB, Owen JA, Raymond-Barker P (2015). Is this elderly patient dehydrated? Diagnostic accuracy of hydration assessment using physical signs, urine, and saliva markers. J Am Med Dir Assoc.

[35] Kafri MW, Myint PK, Doherty D (2013). The diagnostic accuracy of multi-frequency bioelectrical impedance analysis in diagnosing dehydration after stroke. Med Sci Monit.

[36] Pfortmueller CA, Wiemann C, Funk GC (2014). Hypoglycemia is associated with increased mortality in patients with acute decompensated liver cirrhosis. J Crit Care.

[37] Sjöstrand F, Rodhe P, Berglund E (2013). The use of a noninvasive hemoglobin monitor for volume kinetic analysis in an emergency room setting. Anesth Analg.

[38] van Wijk N, Studer B, van den Berg CA (2022). Evident lower blood levels of multiple nutritional compounds and highly prevalent malnutrition in sub-acute stroke patients with or without dysphagia. Front Neurol.

[39] Walsh NP, Fortes MB, Raymond-Barker P (2012). Is whole-body hydration an important consideration in dry eye?. Invest Ophthalmol Vis Sci.

[40] Bech CB, Svendsen JA, Knudsen AW (2023). The association between malnutrition and dehydration in older adults admitted to a geriatric unit: An observational study. Clin Nutr ESPEN.

[41] Jespersen JB, Beck AM, Munk T (2023). Low-intake dehydration and nutrition impact symptoms in older medical patients - A retrospective study. Clin Nutr ESPEN.

[42] Lauriola M, Mangiacotti A, D’Onofrio G (2018). Neurocognitive Disorders and Dehydration in Older Patients: Clinical Experience Supports the Hydromolecular Hypothesis of Dementia. Nutrients.

[43] Miller C, Jones S, Timoroska A-M (2019). Incidence and identification of dehydration in acute stroke: an observational study.

[44] Nagae M, Umegaki H, Komiya H (2023). Dehydration and hospital-associated disability in acute hospitalized older adults. Eur Geriatr Med.

[45] Nielsen RL, Andersen AL, Kallemose T (2024). Evaluation of Multi-Frequency Bioelectrical Impedance Analysis against Dual-Energy X-ray Absorptiometry for Estimation of Low Muscle Mass in Older Hospitalized Patients. JCM.

[46] Sabanovic K, Skjøde Damsgaard EM, Gregersen M (2022). Preoperative dehydration identified by serum calculated osmolarity is associated with severe frailty in patients with hip fracture. Clin Nutr ESPEN.

[47] Sanson G, Marzinotto I, Matteis D (2021). Impaired hydration status in acutely admitted older patients: prevalence and impact on mortality. Age Ageing.

[48] Wojszel ZB (2020). Impending Low Intake Dehydration at Admission to A Geriatric Ward- Prevalence and Correlates in a Cross-Sectional Study. Nutrients.

[49] Zanetti M, Marzaro G, Colle P (2022). Predictors of short- and long-term mortality among acutely admitted older patients: role of inflammation and frailty. Aging Clin Exp Res.

[50] Betrosian A, Thireos E, Kofinas G (1999). Bacterial sepsis-induced rhabdomyolysis. Intensive Care Med.

[51] Buoite Stella A, Gaio M, Furlanis G (2020). Prevalence of hypohydration and its association with stroke severity and independence outcomes in acute ischemic stroke patients. J Clin Neurosci.

[52] Farhan S, Vogel B, Baber U (2019). Calculated Serum Osmolality, Acute Kidney Injury, and Relationship to Mortality after Percutaneous Coronary Intervention. Cardiorenal Med.

[53] Fiaux E, Noel D, Armengol G (2015). Usefulness of assessing hydration status in elderly patients over 70 years with suspected deep vein thrombosis. Rev Med Interne.

[54] Gou L, Xiang M, Ran X (2021). Hyperosmolarity Deserves More Attention in Critically Ill COVID-19 Patients with Diabetes: A Cohort-Based Study. Diabetes Metab Syndr Obes.

[55] Khanimov I, Wainstein J, Boaz M (2020). Reduction of serum albumin in non-critically ill patients during hospitalization is associated with incident hypoglycaemia. *Diabetes & Metabolism*.

[56] McCrow J, Morton M, Travers C (2016). Associations Between Dehydration, Cognitive Impairment, and Frailty in Older Hospitalized Patients: An Exploratory Study. J Gerontol Nurs.

[57] Pliquett RU, Schlump K, Wienke A (2020). Diabetes prevalence and outcomes in hospitalized cardiorenal-syndrome patients with and without hyponatremia. BMC Nephrol.

[58] Shen Y, Cheng X, Ying M (2017). Association between serum osmolarity and mortality in patients who are critically ill: a retrospective cohort study. BMJ Open.

[59] Sokolski M, Sokolska JM, Zymlinski R (2019). The significance of plasma osmolarity for in-hospital course and long-term outcome in patients with acute heart failure. Eur J Heart Fail.

[60] Aoki M, Asai M, Nishihori T (2007). The relevance of an elevation in the plasma vasopressin levels to the pathogenesis of Meniere’s attack. J Neuroendocrinol.

[61] Durakovic Z (1997). Does Arterial Hypotension Due to Cardiogenic Shock in Older Patients Lead to Functional Oliguria or to Acute Renal Failure?. Korean J Intern Med.

[62] Palmisano P, Accogli M, Zaccaria M (2014). Relationship between seasonal weather changes, risk of dehydration, and incidence of severe bradyarrhythmias requiring urgent temporary transvenous cardiac pacing in an elderly population. Int J Biometeorol.

[63] Tellini U, Pellizzari L, Corrà L (2005). Hyponatraemia in elderly with cancer. Trends in Medicine.

[64] Oh H, Seo W (2007). Alterations in fluid, electrolytes and other serum chemistry values and their relations with enteral tube feeding in acute brain infarction patients. J Clin Nurs.

[65] Murray J, Doeltgen S, Miller M (2016). Does a Water Protocol Improve the Hydration and Health Status of Individuals with Thin Liquid Aspiration Following Stroke? A Randomized Controlled Trial. Dysphagia.

[66] Gottlieb J, Torres F, Haddad T (2023). A randomized controlled trial of presatovir for respiratory syncytial virus after lung transplant. J Heart Lung Transplant.

[67] Paulis SJC, Everink IHJ, Halfens RJG (2018). Prevalence and Risk Factors of Dehydration Among Nursing Home Residents: A Systematic Review. J Am Med Dir Assoc.

[68] Marra MV, Simmons SF, Shotwell MS (2016). Elevated Serum Osmolality and Total Water Deficit Indicate Impaired Hydration Status in Residents of Long-Term Care Facilities Regardless of Low or High Body Mass Index. J Acad Nutr Diet.

[69] Heybeli C, Uzun O, Smith L (2025). Associations between malnutrition and dehydration among older adults: A cross-sectional observational study. Nutr Clin Pract.

[70] Furuya-Kanamori L, Barendregt JJ, Doi SAR (2018). A new improved graphical and quantitative method for detecting bias in meta-analysis. Int J Evid Based Healthc.

[71] Doi SAR, Barendregt JJ, Khan S (2015). Advances in the meta-analysis of heterogeneous clinical trials I: The inverse variance heterogeneity model. Contemp Clin Trials.

[72] Doi SAR, Barendregt JJ, Khan S (2015). Advances in the meta-analysis of heterogeneous clinical trials II: The quality effects model. Contemp Clin Trials.

