# Supplementary material for: Prevalence of low-intake dehydration in hospitalised older adults: systematic review and meta-analysis
Source: BMJ Public Health. 2026 May 12;4(2):e002985. doi: 10.1136/bmjph-2025-002985 (PMC13182373; doi:10.1136/bmjph-2025-002985)
Supplement: online supplemental file 1 [file bmjph-4-2-s001.pdf]

Supplementary Files for:

Prevalence of low-intake dehydration in hospitalised older adults: systematic review and meta-analysis

Lee Hooper, Ellice Parkinson, Asmaa Saber Abdelhamid, Ke Deng, Onyekwutozia Edozie, Charlotte Fenner, Lydia Frost, Miruna Ganeshamoorthy, Saranya Mohandas, Joseph Robson, Aisha Sharif, Cameron Wolmarans, Diane Kay Bunn

Contents

Supplementary File 1. Search strategies 2
Supplementary File 2. Reasoning behind meta-analytic methods 6
Supplementary File 3. Table of excluded studies with reasons for exclusion........ 8
Supplementary File 4. Characteristics of all 40 included studies 36
Supplementary File 5. Table of hydration data of the 40 included studies 42
Supplementary File 6. Table of fluid intake data 56
Supplementary File 7. Description of risk of bias of all 40 included studies 57
Supplementary File 8. Sensitivity analyses........ 60
Supplementary File 9. Detailed account of SWiM analysis and subgrouping. 61
Supplementary File 10. Subgrouping by participant age in years 66
Supplementary File 11. Subgrouping by setting........ 67
References........ 68

Supplementary File 1. Search strategies

Medline search (Update run 11July 2024)

Database: Ovid MEDLINE(R) ALL <1946 to July 10, 2024>
Search Strategy:
1 aged/ or "aged, 80 and over"/ or frail elderly/ (3533522)
2 exp Incidence/ or exp Prevalence/ (636073)
3 exp Epidemiology/ (28741)
4 (prevalen* or inciden*).ti,ab. (2079352)
5 2 or 3 or 4 (2264464)
6 exp Dehydration/ (15074)
7 exp Drinking/ or exp Beverages/ (191397)
8 (hydrat* or dehydrat* or euhydrat* or hypohydrat*).ti,ab. (123279)
9 ((drink* or beverage* or fluid* or water) adj3 (intake* or consum* or lack* or defici* or provision)).ti,ab. (54776)
10 (osmolarit* or osmolalit*).ti,ab. (24595)
11 6 or 7 or 8 or 9 or 10 (377024)
12 exp animals/ not humans.sh. (5238132)
13 11 not 12 (279646)
14 1 and 5 and 13 (3826)
15 limit 14 to yr="2024 -Current" (77)

Cochrane CENTRAL search (update run 11July2024)

Search Name: Dehydration prevalence Cochrane 10Apr2024
Date Run: 11/07/2024 16:00:59
Comment: Limited to 2024 to 2024

ID	Search	Hits
#1	MeSH descriptor: [Aged] explode all trees	276556
#2	MeSH descriptor: [Prevalence] explode all trees	7419
#3	MeSH descriptor: [Incidence] explode all trees	14390
#4	(prevalen* or inciden*):ti,ab	207941
#5	#2 or #3 or #4	214021
#6	MeSH descriptor: [Dehydration] explode all trees	762
#7	MeSH descriptor: [Drinking] explode all trees	758
#8	MeSH descriptor: [Beverages] explode all trees	9106
#9	(hydrat* or dehydrat* or euhydrat* or hypohydrat*):ti,ab	8006
#10	((drink* or beverage* or fluid* or water*) NEAR/3 (intake* or consum* or lack* or defici* or provision*)):ti,ab	7950
#11	(osmolarit* or osmolalit*):ti,ab	2201
#12	#6 or #7 or #8 or #9 or #10 or #11	25196
#13	#1 and #5 and #12 in Cochrane Reviews, Cochrane Protocols	6
#14	#1 and #5 and #12 with Publication Year from 2024 to 2024, in Trials	6

EMBASE search (update run 11July2024)

Database: Embase <1974 to 2024 July 10>
Search Strategy:
1 aged/ or aged hospital patient/ or frail elderly/ or institutionalized elderly/ or very elderly/ (3885090)
2 Incidence/ or Prevalence/ (1558009)
3 Epidemiology/ (268194)
4 (prevalen* or inciden*).ti,ab. (2961531)
5 2 or 3 or 4 (3485135)
6 Dehydration/ (51676)
7 Drinking/ or exp Beverages/ (309110)
8 (hydrat* or dehydrat* or euhydrat* or hypohydrat*).ti,ab. (145831)
9 ((drink* or beverage* or fluid* or water) adj3 (intake* or consum* or lack* or defici* or provision)).ti,ab. (69734)
10 (osmolarit* or osmolalit*).ti,ab. (30515)
11 6 or 7 or 8 or 9 or 10 (545484)
12 animal/ or nonhuman/ or animal experiment/ (9778971)
13 human/ or human experiment/ (26627057)
14 12 and 13 (2766814)
15 12 not 14 (7012157)
16 11 not 15 (401475)
17 1 and 5 and 16 (7402)
18 limit 17 to yr="2024 -Current" (328)

Proquest Dissertations and Theses Global (update run 11July2024)

Your search for **(TI,AB(prevalence) OR TI,AB(incidence)) AND (TI,AB(aged) OR TI,AB((“older adult” OR “older adults”)) OR TI,AB(“over 65*”) AND TI,AB(elder*)) AND (TI,AB(dehydrat*) OR TI,AB(fluid*) OR TI,AB(hydrat*) OR TI,AB(osmolal*) OR TI,AB(osmolar*)) AND (pd(20240101-20240801))** found 0 results.

Cinahl search (update run 11July2024)

#	Query	Limiters/Expanders	Last Run Via	Results
S14	S3 AND S4 AND (S5 or S6 or S8 or S9 or S10 or S11)	Limiters - Publication Date: 20220101-20241231<br>Expanders - Apply related words; Apply equivalent subjects<br>Search modes - Find all my search terms	Interface - EBSCOhost Research Databases<br>Search Screen - Advanced Search<br>Database - CINAHL Ultimate	3
S13	S3 AND S4 AND (S5 or S6 or S8 or S9 or S10 or S11)	Limiters - Publication Date: 20220101-20221231<br>Expanders - Apply related words; Apply equivalent subjects<br>Search modes - Find all my search terms	Interface - EBSCOhost Research Databases<br>Search Screen - Advanced Search<br>Database - CINAHL Ultimate	3
S12	S3 AND S4 AND (S5 or S6 or S8 or S9 or S10 or S11)	Expanders - Apply related words; Apply equivalent subjects<br>Search modes - Find all my search terms	Interface - EBSCOhost Research Databases<br>Search Screen - Advanced Search<br>Database - CINAHL Ultimate	13
S11	TI (osmolal* or osmolar*) OR AB (osmolal* or osmolar*)	Expanders - Apply related words; Apply equivalent subjects<br>Search modes - Find all my search terms	Interface - EBSCOhost Research Databases<br>Search Screen - Advanced Search<br>Database - CINAHL Ultimate	2,313
S10	TI hydrat* OR AB hydrat*	Expanders - Apply related words; Apply equivalent subjects<br>Search modes - Find all my search terms	Interface - EBSCOhost Research Databases<br>Search Screen - Advanced Search<br>Database - CINAHL Ultimate	6,832
S9	TI dehydrat* OR AB dehydrat*	Expanders - Apply related words; Apply equivalent subjects<br>Search modes - Find all my search terms	Interface - EBSCOhost Research Databases<br>Search Screen - Advanced Search<br>Database - CINAHL Ultimate	5,260
S8	TI fluid* OR AB fluid*	Expanders - Apply related words; Apply equivalent subjects<br>Search modes - Find all my search terms	Interface - EBSCOhost Research Databases<br>Search Screen - Advanced Search<br>Database - CINAHL Ultimate	68,670
S7	MH ('TI' or 'AB' (fluid*))	Expanders - Apply related words; Apply equivalent subjects<br>Search modes - Find all my search terms	Interface - EBSCOhost Research Databases<br>Search Screen - Advanced Search<br>Database - CINAHL Ultimate	0

S6	MH "Dehydration+"	Expanders - Apply related words; Apply equivalent subjects Search modes - Find all my search terms	Interface - EBSCOhost Research Databases Search Screen - Advanced Search Database - CINAHL Ultimate	4,577
S5	MH "Beverages+"	Expanders - Apply related words; Apply equivalent subjects Search modes - Find all my search terms	Interface - EBSCOhost Research Databases Search Screen - Advanced Search Database - CINAHL Ultimate	42,305
S4	MH "Aged+"	Expanders - Apply related words; Apply equivalent subjects Search modes - Find all my search terms	Interface - EBSCOhost Research Databases Search Screen - Advanced Search Database - CINAHL Ultimate	970,397
S3	('TI' or 'AB' (incidence)) OR ('TI' or 'AB' (prevalence))	Expanders - Apply related words; Apply equivalent subjects Search modes - Find all my search terms	Interface - EBSCOhost Research Databases Search Screen - Advanced Search Database - CINAHL Ultimate	4,890
S2	'TI' or 'AB' (incidence)	Expanders - Apply related words; Apply equivalent subjects Search modes - Find all my search terms	Interface - EBSCOhost Research Databases Search Screen - Advanced Search Database - CINAHL Ultimate	4,561
S1	S2 'TI' or 'AB' (incidence)	Expanders - Apply related words; Apply equivalent subjects Search modes - Find all my search terms	Interface - EBSCOhost Research Databases Search Screen - Advanced Search Database - CINAHL Ultimate	212

Supplementary File 2. Reasoning behind meta-analytic methods

Questions relevant for meta-analysis in prevalence reviews include:

- What type of data transformation should we use (untransformed, logit transformed or double arcsine transformed)?
- What form of meta-analytic model should we use (inverse variance fixed effects, the inverse variance heterogeneity model fixed effects, der Simonian and Laird random effects or quality effects)?
- How should we assess publication (small study) bias (funnel plots or Doi plots)?

What type of data transformation should we use for prevalence data?

A simulation study comparing logit, double arcsine transformation and no transformation of prevalence data and found that double arcsine was preferable (1). All three models performed similarly where the true prevalence was close to 0.5, but double arcsine transformation performed best for true prevalences further from 0.5. For this reason our primary method of analysis used double arcsine transformation.

Other authors have (2) raised concerns that double arcsine transformation works well for a single study, but less well in the context of meta-analysis of studies with different sample sizes. For that reason we ran sensitivity analyses of meta-analyses of at least 4 studies using logit transformation. We also ran sensitivity analyses to assess robustness of the findings to:

- Removal of single outlying studies
- Removal of studies at higher risk of bias (removing studies with Qi<0.5)

What form of meta-analytic model should we use?

While there may be a case to summarise prevalence data as a single proportion using meta-analysis, this may not be useful if the prevalence of dehydration varies widely between populations of hospitalised older adults (heterogeneity is high) (3). We planned to be cautious in this regard – to describe the prevalence of dehydration in different studies narratively, but also to summarise through meta-analysis where feasible.

Traditionally in meta-analysis we consider fixed and random effects models, and random effects models are strongly recommended for meta-analysis of prevalence data (3-6). But other models are available (7), including the inverse variance heterogeneity model developed to support meta-analysis of more heterogeneous data and considered appropriate for prevalence data (8), and quality effects analysis which weighs the analysis according to quality assessment so that stronger studies carry more weight, again recommended specifically for prevalence data when heterogeneity is high (9, 10).

For these reasons we presented prevalence data from included studies, discussing the mean, 95% CI, median, IQR and range of these prevalences, tabulating and summarising them narratively. In addition we conducted random effects meta-analysis as our primary

meta-analytic model, where meta-analysis appeared feasible. We ran quality effects models of meta-analysis as sensitivity analyses.

Heterogeneity between studies

As true heterogeneity in prevalence of low-intake dehydration is expected between populations of hospitalised older adults, the I^2 statistic (which describes variability between proportions expressed as a percentage (11)) was assumed likely to be high, but appropriate for prevalence data (5). Chi squared and tau squared tests can also be used to assess heterogeneity (5) but we chose to describe heterogeneity using I^2.

Assessment of publication bias

Funnel plots are recommended by JBI for assessment of publication bias (6), but they are not recommended by other methodologists (5). Indeed funnel plots showed asymmetry despite a lack of publication bias in models (12). We decided to use and report a new tool – Doi plots, which are reported to work well even with fewer than ten studies, but to interpret them tentatively (13).

Continuity correction

Post-hoc we realised that we were unable to run some forms of the sensitivity analysis meta-analyses due to a zero in the number of people with dehydration for one study. We applied the standard continuity correction of 0.5 as recommended (Meta-XL handbook (14)).

Supplementary File 3. Table of excluded studies with reasons for exclusion

Number	Reference	Exclusion reason
1	Abdul Wahab P, Mohd Yusoff D, Abdul Kadir A, Ali SH, Yeong Yeh L. Prevalence, Symptoms, and Associated Factors of Chronic Constipation Among Older Adults in North-East of Peninsular Malaysia. Clinical Nursing Research. 2022;31(2):348-55.	Wrong hydration measure
2	Ahn HJ, Chun MH, Lee J. Compliance and effect of thickener use in dysphagia patients with brain lesions: An observational pilot study. Medicine (United States). 2022;101(38):E30600.	Wrong hydration measure
3	Al Farsi RS, Al Alawi AM, Al Huraizi AR, Al-Saadi T, Al-Hamadani N, Al Zeedy K, et al. Delirium in Medically Hospitalized Patients: Prevalence, Recognition and Risk Factors: A Prospective Cohort Study. Journal of Clinical Medicine. 2023;12(12) (no pagination).	Wrong hydration measure
4	Al Otaibi T, Nagib A, Nair P, Halim MA, Khaled M, Hammad MA, et al. Acute Kidney Injury Among COVID-19-Positive Patients Is Associated With Higher Mortality: A Single-Center Experience. Exp Clin Transplant. 2024;22(Suppl 1):290-8.	Mean <65 years
5	Alagiakrishnan K, Bhanji RA, Kurian M. Evaluation and management of oropharyngeal dysphagia in different types of dementia: a systematic review. Archives of Gerontology & Geriatrics. 2013;56(1):01-Sep.	Wrong study design
6	Albanil RO, Garcia JC, Bueno BI, Pareja CC. Preventive isolation due to SARS-COV2 and delirium in hospitalized geriatric patients. European Geriatric Medicine. 2021;12(SUPPL 1):S189.	No hydration measure
7	Albert SG, Nakra BR, Grossberg GT, Caminal ER. Vasopressin response to dehydration in Alzheimer's disease. J Am Geriatr Soc. 1989;37(9):843-7.	Not hospital or medical setting
8	Alomari MA, Khabour OF, Alzoubi KH, Keewan E. Changes in dietary habits and eating behaviors during COVID-19 induced confinement: A study from Jordan. Human Nutrition and Metabolism. 2022;30:200169.	Wrong hydration measure
9	Alonso M, Trujillo Rodriguez F, Rubio Rubio JM, Vivancos Escobar JA, Aznar Reig A. [Investigation of the hypoosmolal syndrome in carcinomas of the lung (author's transl)]. Medicina Clinica. 1979;72(7):292-4.	Inclusion unclear - no author reply
10	Ando H, Isobe S, Amano T, Yamada T, Ohtsubo H, Yuba M, et al. Predictors of worsening renal function after computed tomography coronary angiography: assessed by cystatin C. Journal of cardiovascular computed tomography. 2012;6(1):31-6.	No hydration measure
11	Ang TE, Bivard A, Levi C, Ma H, Hsu CY, Campbell B, et al. Multi-modal CT in acute stroke: wait for a serum creatinine before giving intravenous contrast? No! International Journal of Stroke. 2015;10(7):1014-7.	No hydration measure
12	Angeloni N, Masevicius F, Outi I, Gutierrez M, Sterman S, Morales J, et al. Serum Sodium Behaviour During the Recovery of Renal Function in Critically Ill Adult Patients: Multicenter Prospective Cohort Study. Canadian Journal of Anesthesia. 2022;69(1 SUPPL):S129-S31.	Wrong hydration measure

13	Arinzon Z, Peisakh A, Schrire S, Berner YN. Delirium in long-term care setting: indicator to severe morbidity. Archives of Gerontology & Geriatrics. 2011;52(3):270-5.	Not hospital or medical setting
14	Arnau-Barres I, Miranda O, Canchucaja L, Vazquez O, Monllau JC, Martinez S, et al. Delirium in patients with acute hip fracture admitted to a multidisciplinary orthogeriatrics unit: Incidence, characteristics and associated risk factors. European Geriatric Medicine. 2018;9 (Supplement 1):S75.	Wrong hydration measure
15	Ashley S, Khan MS, Minhas AMK, Greene S. Clinical Course and Outcomes in Acute Heart Failure with Moderate-Severe Mitral or Tricuspid Regurgitation. Journal of the American College of Cardiology. 2024;83(13 Supplement):649.	No hydration measure
16	Atciyurt K, Heybeli C, Smith L, Veronese N, Soysal P. The prevalence, risk factors and clinical implications of dehydration in older patients: a cross-sectional study. Acta Clinica Belgica.01-Jul.	Not hospital or medical setting
17	Ates I, Ozkayar N, Toprak G, Yilmaz N, Dede F. Factors associated with mortality in patients presenting to the emergency department with severe hypernatremia. Internal & Emergency Medicine. 2016;11(3):451-9.	Inclusion unclear - no author reply
18	AvendaÃ±o-CÃ©spedes A, GarcÃ_a-Cantos N, GonzÃ¡lez-Teruel Mdel M, MartÃ_nez-GarcÃ_a M, Villarreal-Bocanegra E, Oliver-Carbonell JL, et al. Pilot study of a preventive multicomponent nurse intervention to reduce the incidence and severity of delirium in hospitalized older adults: MID-Nurse-P. Maturitas. 2016;86:86-94.	Wrong hydration measure
19	Badri VK, Chaitra KR, Devakar S, Neelopant SA. Clinical Profile and Etiology of Chronic Kidney Disease in a Tertiary Care Centre in Karnataka. International Journal of Academic Medicine and Pharmacy. 2023;5(6):1060-6.	Mean <65 years
20	Bae SJ, Kim K, Yun SJ, Lee SH. Predictive performance of blood urea nitrogen to serum albumin ratio in elderly patients with gastrointestinal bleeding. American Journal of Emergency Medicine. 2021;41:152-7.	No hydration measure
21	Baena AV, Allam MF, Del Castillo AS, Diaz-Molina C, Requena Tapia MJ, Abdel-Rahman AG, et al. Urinary bladder cancer risk factors in men: a Spanish case-control study. European Journal of Cancer Prevention. 2006;15(6):498-503.	No hydration measure
22	Bagshaw SM, Townsend DR, McDermid RC. Disorders of sodium and water balance in hospitalized patients. Can J Anaesth. 2009;56(2):151-67.	Wrong study design
23	Baldursdottir MB, Andresson JA, Jonsdottir S, Benediktsson H, Kalaitzakis E, Bjornsson ES. Ischemic Pancreatitis Is an Important Cause of Acute Pancreatitis in the Intensive Care Unit. Journal of Clinical Gastroenterology. 2023;57(1):97-102.	Mean <65 years

24	Baraldi LG, Steele EM, Louzada MLC, Monteiro CA. Associations between ultraprocessed food consumption and total water intake in the US population. Journal of the Academy of Nutrition and Dietetics. 2021;121(9):1695-703.	Wrong hydration measure
25	Beaver ME, Matheny KE, Roberts DB, Myers JN. Predictors of weight loss during radiation therapy. Otolaryngol Head Neck Surg. 2001;125(6):645-8.	Pre or post operative fluids
26	Beck AM, Seemer J, Knudsen AW, Munk T. Narrative Review of Low-Intake Dehydration in Older Adults. Nutrients. 2021;13(9).	Wrong study design
27	Begum MN, Johnson CS. A review of the literature on dehydration in the institutionalized elderly. e-SPEN. 2010;5(1):e47-e53.	Wrong study design
28	Bellafronte NT, Cuadrado GB, Chiarello PG. Assessment Of Body Composition By Bioelectrical Impedance In Peritoneal Dialysis. Clinical Nutrition ESPEN. 2023;Conference: ESPEN 2022 Congress. Vienna Austria. 54:563-4.	Mean <65 years
29	Bhatnagar D, Weinkove C. Serious hypernatraemia in a hospital population. Postgraduate Medical Journal. 1988;64(752):441-3.	No hydration measure
30	Bhattacharya A, Debnath D, Saha A, Saha S. Effect of Supplemental Pre-Operative Fluid on the Incidence of Postoperative Nausea and Vomiting Among Patients Undergoing Laparoscopic Cholecystectomy at Tripura medical College and DR. B.R. Ambedkar Teaching Hosptal. Research Journal of Medical Sciences. 2024;18(5):65-9.	No hydration measure
31	Bodrya K, Patel K, Patel N, Patel D, Modi R, Matin A. Jaundice on Presentation Is Associated with Higher In-Patient Mortality and Complications in Patients Admitted for Acute Pancreatitis: A Retrospective Study Based on National Inpatient Sample Database. Gastroenterology Research and Practice. 2022;2022:5048061.	Wrong hydration measure
32	Bonaventura M, Zanotti R. [Effectiveness of IPD treatment for delirium prevention in hospitalized elderly. A controlled randomized clinical trial]. Prof Inferm. 2007;60(4):230-6.	Wrong hydration measure
33	Bond VE, Doeltgen S, Kleinig T, Murray J. Dysphagia-related acute stroke complications: A retrospective observational cohort study. J Stroke Cerebrovasc Dis. 2023;32(6):107123.	Wrong hydration measure
34	Bond VE, Doeltgen S, Kleinig T, Murray J. Dysphagia-related acute stroke complications: A retrospective observational cohort study. Journal of Stroke and Cerebrovascular Diseases. 2023;32(6) (no pagination).	Wrong hydration measure
35	Bonelli J, Jancuska M. [Dehydration in the aged]. Wiener Medizinische Wochenschrift. 1984;134(22):487-93.	Participants chosen for dehydration

36	Borsuk DJ, Studniarek A, Marecik SJ, Park JJ, Kochar K. Protocol-Based Intravenous Fluid Hydration for Newly Created Ileostomies Decreases Readmissions Secondary to Dehydration. American Surgeon. 2021;87(6):897-902.	Mean <65 years
37	Botigue T, Miranda J, Escobar-Bravo MA, Lavedan A, Roca J, Masot O. Analysis of dehydration in older people in a nursing home in Spain: prevalence and associated factors. [Spanish]. Nutricion hospitalaria. 2021;17.	No hydration measure
38	Botigue T, Miranda J, Escobar-Bravo MA, Lavedan A, Roca J, Masot O. Analysis of dehydration in older people in a nursing home in Spain: prevalence and associated factors. Nutricion Hospitalaria. 2021;38(2):252-9.	Not hospital or medical setting
39	Brants HA, Lowik MR, Westenbrink S, Hulshof KF, Kistemaker C. Adequacy of a vegetarian diet at old age (Dutch Nutrition Surveillance System). Journal of the American College of Nutrition. 1990;9(4):292-302.	Not hospital or medical setting
40	Brooks CJ, Gortmaker SL, Long MW, Cradock AL, Kenney EL. Racial/ethnic and socioeconomic disparities in hydration status among US adults and the role of tap water and other beverage intake. American Journal of Public Health. 2017;107(9):1387-94.	Mean <65 years
41	Buchkremer F, Schuetz P, Mueller B, Segerer S. Classifying Hypotonic Hyponatremia by Projected Treatment Effects - A Quantitative 3-Dimensional Framework. Kidney International Reports. 2023;8(12):2720-32.	Participants chosen for dehydration
42	Buck J, Fromings Hill J, Collins R, Booth J, Fleming J. Effectiveness of non-pharmacological interventions delivered at home for urinary and faecal incontinence with homebound older people: systematic review of randomised controlled trials. Age and Ageing. 2024;53(6) (no pagination).	Wrong study design
43	Bunn D, Jimoh F, Wilsher SH, Hooper L. Increasing fluid intake and reducing dehydration risk in older people living in long-term care: a systematic review. Journal of the American Medical Directors Association. 2015;16(2):101-13.	Wrong study design
44	Burton JK, Craig LE, Yong SQ, Siddiqi N, Teale EA, Woodhouse R, et al. Non-pharmacological interventions for preventing delirium in hospitalised non-ICU patients. Cochrane Database of Systematic Reviews. 2021;7:CD013307.	No hydration measure
45	Butt MI, Aljamei H, Riazuddin M, AlHaqbani L, Albalwi R, Abothenain FFM, et al. Efficacy and safety of empagliflozin: a real-world experience from Saudi Arabia. Annals of Saudi Medicine. 2023;43(1):50-6.	Wrong hydration measure
46	Casimiro C, Garcia-de-Lorenzo A, Usan L. Prevalence of decubitus ulcer and associated risk factors in an institutionalized Spanish elderly population. Nutrition. 2002;18(5):408-14.	Not hospital or medical setting
47	Castro JMd, Kreitzman SM. A microregulatory analysis of spontaneous human feeding patterns. Physiology & Behavior. 1985;35:329-35.	Not hospital or medical setting

48	Castro JMd. Age-Related Changes in Natural Spontaneous Fluid Ingestion and Thirst in Humans. Journal of Gerontology. 1992;47(5):P321-P30.	Wrong hydration measure
49	Cebola M, Mahendra A, Pombo M, Marques B, Pinto H, Carolino E, et al. Comparison of nutritional status among elderly in hospital and in community environments. European Geriatric Medicine. 2016;7 (Supplement 1):S195.	No hydration measure
50	Ceravolo MJ, Alfano C, Geniere Nigra S, Cuzzoni G, Venturini L, Ricevuti G. Delirium in a geriatric population suffering from dementia: Complication or picture at the onset? European Geriatric Medicine. 2017;8 (Supplement 1):S86.	No hydration measure
51	Chadwick NM, Perman ML, Leavai F, Kaspar A. Acute Kidney Injury: Incidence, aetiology, management and outcome measures of a Samoan case series. Annals of Medicine and Surgery. 2022;75:103362.	Wrong hydration measure
52	Chatot-Henry C, Slimani S, Tenebay C. Nosocomial urinary infections in elderly persons. [French]. Soins. 2004;Gerontologie.(49):37-9.	No hydration measure
53	Chen LK, Lin MH, Hwang SJ, Chen TW. Hyponatremia among the institutionalized elderly in 2 long-term care facilities in Taipei. Journal of the Chinese Medical Association: JCMA. 2006;69(3):115-9.	Not hospital or medical setting
54	Chen Y, Peng Y, Zhang X, Chen L, Lin Y. Influence of Impaired Hydration Status on Postoperative in-Hospital Death in Patients with Acute Type A Aortic Dissection. Int J Gen Med. 2023;16:4419-28.	Mean <65 years
55	Chewcharat A, Curhan G. Trends in the prevalence of kidney stones in the United States from 2007 to 2016. Urolithiasis. 2021;49(1):27-39.	Wrong study design
56	Claure-Del Granado R, Ramirez-Yapura SG, Burdmann EA, Yu L, Younes-Ibrahim M, Ferreiro A, et al. Differences on outcomes between AKI and AKI on CKD in community-acquired AKI. Journal of the American Society of Nephrology. 2019;30:137.	Mean <65 years
57	Claverie P, Laroche ML, Karam HH, Geniaux H. Severe hyponatremia in an Emergency Department in 2015: A retrospective observational study. Fundamental and Clinical Pharmacology. 2017;31 (Supplement 1):35.	Participants chosen for dehydration
58	Clegg A, Siddiqi N, Heaven A, Young J, Holt R. Interventions for preventing delirium in older people in institutional long-term care. Cochrane Database of Systematic Reviews. 2014(1):CD009537.	Wrong study design
59	Cohen SM, Wexner SD, Binderow SR, Nogueras JJ, Daniel N, Ehrenpreis ED, et al. Prospective, randomized, endoscopic-blinded trial comparing precolonoscopy bowel cleansing methods. Diseases of the Colon & Rectum. 1994;37(7):689-96.	Pre or post operative fluids
60	Colligan B, Lin JY, Hansen D. Hypernatremic dehydration in the elderly nursing home population. Journal of the American Geriatrics Society. 2018;66 (Supplement 2):S272.	Not hospital or medical setting
61	Crook MA, Velauthar U, Moran L, Griffiths W. Review of investigation and management of severe hyponatraemia in a hospital population. Annals of Clinical Biochemistry. 1999;36(Pt 2):158-62.	Inclusion unclear - no author reply

62	Crowe MJ, Forsling ML, Rolls BJ, Phillips PA, Ledingham JG, Smith RF. Altered water excretion in healthy elderly men. Age Ageing. 1987;16(5):285-93.	Not hospital or medical setting
63	Cruise PA. BLADDER BEHAVIOR IN NONINSTITUTIONALIZED FRAIL ELDERLY PERSONS. 1987(8801305):157.	Not hospital or medical setting
64	Cuesta M, Slattery D, Goulden EL, Gupta S, Tatro E, Sherlock M, et al. Hyponatraemia in patients with community-acquired pneumonia; prevalence and aetiology, and natural history of SIAD. Clinical Endocrinology. 2019;90(5):744-52.	Mean <65 years
65	Cui X, Xie B, Wang H, Liu F, Mei L, Qin F, et al. Preventing contrast-induced acute kidney injury with probucol and hydration in patients with coronary heart disease: A systematic review and meta-analysis of randomized controlled trials. Medicine (United States). 2023;102(11):E33273.	Wrong study design
66	Culp K, Mentes J, Wakefield B. Hydration and acute confusion in long-term care residents. Western Journal of Nursing Research. 2003;25(3):251-66; discussion 67.	Not hospital or medical setting
67	Culp K, Tripp-Reimer T, Wadle K, Wakefield B, Akins J, Mobily P, et al. Screening for acute confusion in elderly long-term care residents. Journal of Neuroscience Nursing. 1997;29(2):86-8, 95.	Not hospital or medical setting
68	Cumming K, Hoyle GE, Hutchison JD, Soiza RL. Prevalence, incidence and etiology of hyponatremia in elderly patients with fragility fractures. PLoS ONE [Electronic Resource]. 2014;9(2):e88272.	No hydration measure
69	Das S, Sengupta U, Ganguly S. A Study of Etiology of Acute Kidney Injury and Acute on Chronic Kidney Disease Amongst in-Hospital Patients in a Tertiary Care Centre in India. Nephrology Dialysis Transplantation. 2023;38(Supplement 1):i1259.	Wrong hydration measure
70	Dash SC, Sundaray NK, Rajesh B, Pagad T. Hyponatremia in elderly in-patients. Journal of Clinical and Diagnostic Research. 2019;13(2):OC01-OC4.	Inclusion unclear - no author reply
71	de Castro JM. The relationship of spontaneous macronutrient and sodium intake with fluid ingestion and thirst in humans. Physiol Behav. 1991;49(3):513-9.	Mean <65 years
72	De La Motte L, Nordenvall C, Martling A, Buchli C. Preoperative use of angiotensin-converting enzyme inhibitors, angiotensin II receptor blockers and diuretics increases the risk of dehydration after ileostomy formation: Population-based cohort study. BJS Open. 2024;8(3) (no pagination).	Wrong hydration measure
73	de la Motte L, Nordenvall C, Martling A, Buchli C. Preoperative use of angiotensin-converting enzyme inhibitors, angiotensin II receptor blockers and diuretics increases the risk of dehydration after ileostomy formation: population-based cohort study. BJS open. 2024;8(3):08.	Wrong hydration measure
74	Deisler L, Wirth R, Frilling B, Janneck M, Rosler A. Hydration Status Assessment in Older Patients. Dtsch. 2023;120(40):663-9.	Wrong study design

75	Devriese SHIMMVOH. De Belgische Voedselconsumptiepeiling 1-2004. D/2006/2505/17, IPH/EPI REPORTS No 2006-016. 2006.	Not hospital or medical setting
76	Dewiasty E, Setiati S, Agustina R, Roosheroe AG, Abdullah M, Istanti R, et al. Prevalence of lactose intolerance and nutrients intake in an older population regarded as lactase non-persistent. Clinical Nutrition ESPEN. 2021;43:317-21.	Not hospital or medical setting
77	Diago D, Morillo J, Rodriguez-Mier V, Rivera FR, Mari MV. The Great Contender: A Descriptive Analysis of Modifiable Risk Factors and Readmission Rates among Patients with Congestive Heart Failure in Southern Puerto Rico. Journal of the American College of Cardiology. 2024;83(13 Supplement):770.	Participants chosen for dehydration
78	Docherty NG, Delles C, D'Haese P, Layton AT, Martinez-Salgado C, Vervaet BA, et al. Haemodynamic frailty - A risk factor for acute kidney injury in the elderly. Ageing Research Reviews. 2021;70:101408.	Wrong study design
79	Dominguez LJ, Donat-Vargas C, Banegas JR, Barbagallo M, Rodriguez-Artalejo F, Guallar-Castillon P. Adherence to a Healthy Beverage Score Is Associated with Lower Frailty Risk in Older Adults. Nutrients. 2022;14(18).	Wrong hydration measure
80	Dong Z, Song J, Ge M, Lin C, Zhang J, Chen J, et al. Effectiveness of a multidisciplinary comprehensive intervention model based on the Hospital Elderly Life Program to prevent delirium in patients with severe acute pancreatitis. Ann Palliat Med. 2020;9(4):2221-8.	No hydration measure
81	Dorner TE, Lackinger C, Haider S, Luger E, Kapan A, Luger M, et al. Nutritional intervention and physical training in malnourished frail community-dwelling elderly persons carried out by trained lay buddies: study protocol of a randomized controlled trial. BMC Public Health. 2013;13:1232.	Not hospital or medical setting
82	Dou L, Rao W, Liu Y, Zhang Y, He S, Xue L, et al. The Feasibility and Safety of Endoscopic Submucosal Dissection for Circumferential Superficial Esophageal Squamous Cell Neoplasms. Journal of Clinical Medicine. 2023;12(2) (no pagination).	No hydration measure
83	Duan H, Sun C, Zhu Y, Liu Q, Du Y, Lin H, et al. Association of Dietary Habits with Mild Cognitive Impairment among Elderly in Rural Area of North China. Current Alzheimer Research. 2021;18(3):256-64.	Wrong hydration measure
84	Dubrava M, Janosiova J, Borucky J, Koromhazova A, Majekova J, Stugelova K, et al. Polymorbidity in seniors hospitalized for COVID-19. Bratislavske Lekarske Listy. 2022;123(5):339-46.	Wrong hydration measure
85	Dunne TE, Neargarder SA, Cipolloni PB, Cronin-Golomb A. Visual contrast enhances food and liquid intake in advanced Alzheimer's disease. Clinical Nutrition. 2004;23(4):533-8.	Not hospital or medical setting
86	El-Sharkawy AM, Virdee A, Wahab A, Humes DJ, Sahota O, Devonald MAJ, et al. Dehydration and clinical outcome in hospitalised older adults: A cohort study. European Geriatric Medicine. 2017;8(1):22-9.	Wrong hydration measure
87	Engelheart S, Andren D, Repsilber D, Berteus Forslund H, Brummer RJ. Nutritional status in older people - An explorative analysis. Clinical Nutrition ESPEN. 2021;46:424-33.	Not hospital or medical setting

88	Engelheart S, Forslund HB, Brummer RJ, Ljungqvist O. Dehydration and loss of appetite: Key nutrition features in older people receiving home health care. Nutrition (Burbank, Los Angeles County, Calif). 2021;91-92:111385.	Not hospital or medical setting
89	Enhorning S, Melander O, Engstrom G, Elmstahl S, Lind L, Nilsson PM, et al. Seasonal variation of vasopressin and its relevance for the winter peak of cardiometabolic disease: A pooled analysis of five cohorts. Journal of Internal Medicine. 2022;292(2):365-76.	Mean <65 years
90	Eshak ES, Muraki I, Imano H, Yamagishi K, Tamakoshi A, Iso H. Manganese intake from foods and beverages is associated with a reduced risk of type 2 diabetes. Maturitas. 2021;143:127-31.	Mean <65 years
91	Eshetu B, Worede A, Fentie A, Chane E, Fetene G, Wondifraw H, et al. Assessment of Electrolyte Imbalance and Associated Factors Among Adult Diabetic Patients Attending the University of Gondar Comprehensive Specialized Hospital, Ethiopia: A Comparative Cross-Sectional Study. Diabetes, Metabolic Syndrome and Obesity. 2023;16:1207-20.	No hydration measure
92	Esmeray G, Senturan L, Doventas A. A study on efficacy of hydration administered by subcutaneous infusion in geriatric patients. Turk Geriatri Dergisi. 2018;21(3):438-45.	IV fluids
93	Fabian TJ, Amico JA, Kroboth PD, Mulsant BH, Corey SE, Begley AE, et al. Paroxetine-induced hyponatremia in older adults: a 12-week prospective study. Archives of Internal Medicine. 2004;164(3):327-32.	Not hospital or medical setting
94	Fasugba O, Mitchell BG, McInnes E, Koerner J, Cheng AC, Cheng H, et al. Increased fluid intake for the prevention of urinary tract infection in adults and children in all settings: a systematic review. Journal of Hospital Infection. 2020;104(1):68-77.	Not hospital or medical setting
95	Ferry M, Hininger-Favier I, Sidobre B, Mathey MF. Food and fluid intake of the SENECA population residing in Romans, France. J Nutr Health Aging. 2001;5(4):235-7.	Not hospital or medical setting
96	Fish DR, Mancuso CA, Garcia-Aguilar JE, Lee SW, Nash GM, Sonoda T, et al. Readmission After Ileostomy Creation: Retrospective Review of a Common and Significant Event. Ann Surg. 2017;265(2):379-87.	Pre or post operative fluids
97	Fletcher SJ, Slaymaker AE, Bodenham AR, Vucevic M. Urine colour as an index of hydration in critically ill patients. Anaesthesia. 1999;54(2):189-92.	Mean <65 years
98	Fotso Soh J, Beaulieu S, Trepiccione F, Linnaranta O, Torres-Platas G, Platt RW, et al. A double-blind, randomized, placebo-controlled pilot trial of atorvastatin for nephrogenic diabetes insipidus in lithium users. Bipolar Disorders. 2021;23(1):66-75.	Mean <65 years
99	Frangeskou M, Lopez-Valcarcel B, Serra-Majem L. Dehydration in the Elderly: A Review Focused on Economic Burden. Journal of Nutrition, Health & Aging. 2015;19(6):619-27.	Wrong study design

100	Gadalean F, Milas O, Suteanu-Simulescu A, Glavan M, Ienciu S, Mogos M, et al. Delirium among patients admitted to a Nephrology ward-risk factors and association with short-term outcomes: an observational prospective cohort study. Nephrology Dialysis Transplantation. 2024;39(Supplement 1):i2794.	Inclusion unclear - no author reply
101	Gambi N, Dotel Perez F, Dominguez Teba AJ. Dehydration in the elderly treated in the emergency room of the regional hospital. Revista de Neurologia. 2016;62 (2):30.	No hydration measure
102	Gandy J. First Findings of the United Kingdom Fluid Intake Study. Nutrition Today. 2012;47(4):S14-S6.	Not hospital or medical setting
103	Garbern SC, Chu TC, Yang P, Gainey M, Nasrin S, Kanekar S, et al. Clinical and socio-environmental determinants of multidrug-resistant vibrio cholerae 01 in older children and adults in Bangladesh. International Journal of Infectious Diseases. 2021;105:436-41.	Mean <65 years
104	Gautam G, Herath S, Weiss L, Hegde U. Iatrogenic Hyperhydration in Sickle Cell Disease Patients Admitted for Acute Pain Crisis Management: A Retrospective Analysis from a Single Institution. Blood. 2023;142(Supplement 1):7214.	Wrong hydration measure
105	Ghoshal UC, Singh R, Rai S. Prevalence and risk factors of gastroesophageal reflux disease in a rural Indian population. Indian journal of gastroenterology : official journal of the Indian Society of Gastroenterology. 2021.	Mean <65 years
106	Gines A, Escorsell A, Gines P, Salo J, Jimenez W, Inglada L, et al. Incidence, predictive factors, and prognosis of the hepatorenal syndrome in cirrhosis with ascites. Gastroenterology. 1993;105(1):229-36.	Mean <65 years
107	Givens DJ, Karnell LH, Gupta AK, Clamon GH, Pagedar NA, Chang KE, et al. Adverse events associated with concurrent chemoradiation therapy in patients with head and neck cancer. Arch Otolaryngol Head Neck Surg. 2009;135(12):1209-17.	Mean <65 years
108	Glasgow MA, Shields K, Vogel RI, Teoh D, Argenta PA. Postoperative readmissions following ileostomy formation among patients with a gynecologic malignancy. Gynecol Oncol. 2014;134(3):561-5.	Wrong hydration measure
109	Goda M, Yamakura T, Sasaki K, Tajima T, Ueno M. Safety and efficacy of canagliflozin in elderly patients with type 2 diabetes mellitus: a 1-year post-marketing surveillance in Japan. Current Medical Research & Opinion. 2018;34(2):319-27.	No hydration measure
110	Golomb D, Goldberg H, Lavi A, Kafka I, Kleinmann N, Shvero A, et al. Do weather parameters affect the incidence of renal colic in a predominantly warm country? A multicenter study. Journal of Clinical Urology. 2024;17(1):16-21.	Mean <65 years
111	Gordon C, Hewer RL, Wade DT. Dysphagia in acute stroke. British Medical Journal Clinical Research Ed. 1987;295(6595):411-4.	No hydration measure

112	Gu H, Wang L, Cao D, Li W, Ma M. Graded nutritional interventions in patients with dysphagia after stroke: an assessment of the effectiveness of therapeutic strategies for different swallowing functions. International Journal of Neuroscience. 2024.	Inclusion unclear - no author reply
113	HÃ_ussinger D. The role of cellular hydration in the regulation of cell function. Biochem J. 1996;313 (Pt 3)(Pt 3):697-710.	No hydration measure
114	Halland M, Koloski NA, Jones M, Byles J, Chiarelli P, Forder P, et al. Prevalence correlates and impact of fecal incontinence among older women. Diseases of the Colon & Rectum. 2013;56(9):1080-6.	Not hospital or medical setting
115	Hamilton LA, Behal ML, Carter AR, Rowe AS. Patient-Specific Risk Factors Associated With the Development of Hyperchloremia in a Neurocritical Care Intensive Care Unit. Journal of Pharmacy Practice. 2023;36(1):110-6.	IV fluids
116	Haveman-Nies A, Groot LDd, Staveren WVv. Fluid intake of elderly Europeans. The journal of nutrition, health & aging. 1997;1 3:151-5.	Not hospital or medical setting
117	Hazzard E, Walton K, McMahon AT, Milosavljevic M, Tapsell LC. Nutrition-related hospital presentations and admissions among radiotherapy outpatients: a systematic literature review. Journal of Human Nutrition & Dietetics. 2018;31(3):357-69.	Wrong study design
118	Hejazi S, Majd HA, Abedi ZN. Study of association between lifestyle and hemorrhoids among patients referring to Tehran University of medical sciences hospitals in 2007. Medical Sciences Journal of Islamic Azad University. 2008;18(2):Pe127-Pe31, En13.	Mean <65 years
119	Holler JG, Eriksson R, Jensen TO, van Wijhe M, Fischer TK, Sogaard OS, et al. First wave of COVID-19 hospital admissions in Denmark: a Nationwide population-based cohort study. BMC Infectious Diseases. 2021;21(1):39.	No hydration measure
120	Hooper L, Bunn D, Jimoh FO, Fairweather-Tait SJ. Water-loss dehydration and aging. Mechanisms of Ageing & Development. 2014;136-137:50-8.	Wrong study design
121	Hooper L. Why, Oh Why, Are So Many Older Adults Not Drinking Enough Fluid? Journal of the Academy of Nutrition & Dietetics. 2016;116(5):774-8.	Wrong study design
122	Hoorn EJ, Betjes MG, Weigel J, Zietse R. Hypernatraemia in critically ill patients: too little water and too much salt. Nephrol Dial Transplant. 2008;23(5):1562-8.	Mean <65 years
123	Hoorn EJ, Zietse R. Hydration to prevent contrast-induced nephropathy. [Dutch]. Nederlands Tijdschrift voor Geneeskunde. 2018;161:D2442.	Wrong study design
124	Hooton TM, Vecchio M, Iroz A, Tack I, Dornic Q, Seksek I, et al. Effect of Increased Daily Water Intake in Premenopausal Women With Recurrent Urinary Tract Infections: A Randomized Clinical Trial. JAMA Intern Med. 2018;178(11):1509-15.	Participants chosen for dehydration

125	Hosia-Randell H, Suominen M, Muurinen S, Pitkala KH. Use of laxatives among older nursing home residents in Helsinki, Finland. Drugs & Aging. 2007;24(2):147-54.	Not hospital or medical setting
126	Hosie A, Phillips J, Lam L, Kochovska S, Noble B, Brassil M, et al. A Multicomponent Nonpharmacological Intervention to Prevent Delirium for Hospitalized People with Advanced Cancer: A Phase II Cluster Randomized Waitlist Controlled Trial (The PRESERVE Pilot Study). J Palliat Med. 2020;23(10):1314-22.	Wrong hydration measure
127	Hussain SF, Heinze N, Castle CL, Godier-Mcbard LR, Kempapidis T, Gomes RSM. Snapshot of health-related behaviours in adults living with disabilities 1 year into the COVID-19 pandemic: a cross-sectional survey study. BMJ Open. 2022;12(7):e060512.	Wrong hydration measure
128	Indira NB, Reddy KVN, Reddy KN, Kalyan B. Comparison of Clinical Profile and Biochemical Profile in Diabetic Ketoacidosis Patients. Journal of Cardiovascular Disease Research. 2023;14(8):222-38.	Mean <65 years
129	Islam N, Badiuzzaman M, Asifudduza M, Owashak Faysal M, Quadir R, Ali S, et al. Association of Calculated Pre-Procedure Serum Osmolality with Development of Contrast-Induced Nephropathy among Patients Undergoing Percutaneous Coronary Intervention. Journal of Cardiovascular Disease Research. 2023;14(4):1558-72.	Mean <65 years
130	Isobe S, Yamada T, Sato K, Katagiri T, Ohyama H, Hayashi M, et al. Diabetes with preserved renal function is an independent risk factor for renal function deterioration after coronary computed tomography angiography. Journal of Computer Assisted Tomography. 2013;37(5):750-4.	Pre or post operative fluids
131	Iwamoto T, Akazawa M, Ami M, Shimizu T, Umahara T, Takasaki M. [Five elderly patients with cerebral infarction seen during a heat wave]. Nippon Ronen Igakkai Zasshi - Japanese Journal of Geriatrics. 1999;36(8):565-71.	No hydration measure
132	J S, Hegde S, Avinash, Madhusudhan. Serum Calcium Concentration and its Association with Disease Severity in Critically Ill Patients Admitted to Intensive Care Unit. The Journal of the Association of Physicians of India. 2023;71(1):1.	No hydration measure
133	Jiang YW, Zhang YB, Pan A. [Consumption of sugar-sweetened beverages and artificially sweetened beverages and risk of cardiovascular disease: a meta-analysis]. Chung-Hua Yu Fang i Hsueh Tsa Chih [Chinese Journal of Preventive Medicine]. 2021;55(9):1159-67.	No hydration measure
134	Jimenez Mola S, Idoate Gil J, Lopez Lopez K, Lopez Vinas M, Saez Lopez P, Martin Perez E. Oropharyngeal dysphagia in a geriatric outpatient unit. European Geriatric Medicine. 2017;8 (Supplement 1):S79.	Not hospital or medical setting
135	Jodaitis L, Vaillant F, Snacken M, Boland B, Spinewine A, Dalleur O, et al. Orthostatic hypotension and associated conditions in geriatric inpatients. Acta Clinica Belgica. 2015;70(4):251-8.	No hydration measure
136	Justiniano CF, Temple LK, Swanger AA, Xu Z, Speranza JR, Cellini C, et al. Readmissions With Dehydration After Ileostomy Creation: Rethinking Risk Factors. Dis Colon Rectum. 2018;61(11):1297-305.	Wrong hydration measure

137	Kakeshita K, Koike T, Imamura T, Fujioka H, Yamazaki H, Kinugawa K. Altered arginine vasopressin-cyclic AMP-aquaporin 2 pathway in patients with chronic kidney disease. Clinical and Experimental Nephrology. 2022;26(8):788-96.	Not hospital or medical setting
138	Kaki S, Reilly C. Characteristics and outcomes of emergency department patients with acute kidney injury (AKI) stage 2 and 3 electronic alerts at a single UK centre. Nephrology Dialysis Transplantation. 2024;39(Supplement 1):i2826.	Wrong hydration measure
139	Kant AK, Graubard BI, Atchison EA. Intakes of plain water, moisture in foods and beverages, and total water in the adult US populationâ€nutritional, meal pattern, and body weight correlates: National Health and Nutrition Examination Surveys 1999â€2006. The American Journal of Clinical Nutrition. 2009;90(3):655-63.	Not hospital or medical setting
140	Karunaratne TB, Clave P, Ortega O. Complications of oropharyngeal dysphagia in older individuals and patients with neurological disorders: insights from Mataro hospital, Catalonia, Spain. Frontiers in Neurology. 2024;15(no pagination).	No hydration measure
141	Kashiji A, Tajiri M, Chikugo M, Nomura S, Yasui-Yamada S, Tani-Suzuki Y, et al. Hyponatremia is a Prognostic Factor in Patients Receiving Nutrition Support. American Journal of the Medical Sciences. 2021;361(6):744-50.	No hydration measure
142	Ke C, Xiao J, Wang Z, Yu C, Yang C, Hu Z. Characteristics of patients with kidney injury associated with COVID-19. International Immunopharmacology. 2021;96:107794.	Mean <65 years
143	Kenney WL, Chiu P. Influence of age on thirst and fluid intake. Med Sci Sports Exerc. 2001;33(9):1524-32.	Wrong study design
144	Keser I, Cvijetic S, Ilic A, Colic Baric I, Boschiero D, Ilich JZ. Assessment of Body Composition and Dietary Intake in Nursing-Home Residents: Could Lessons Learned from the COVID-19 Pandemic Be Used to Prevent Future Casualties in Older Individuals? Nutrients. 2021;13(5):29.	Not hospital or medical setting
145	Khan J, Mather S, Ismail S, Ngoma P, Chattopadhyay T. Does the implementation of a delirium checklist improve the assessment of delirium in older people? Age and Ageing. 2017;46 (Supplement 1):i1.	No hydration measure
146	Khanasuk Y, Tanavalee A. Low urine output during the first twenty-four hours after total knee arthroplasty. Journal of the Medical Association of Thailand. 2015;98 Suppl 1:S42-8.	Pre or post operative fluids
147	Kiesswetter E, Sieber CC, Volkert D. Nutritional situation of older adults living in German speaking countries. Aktuelle Ernahrungsmedizin. 2016;41(5):362-9.	Wrong study design
148	Kim DK, Shin SD, Ro YS, Song KJ, Jeong J, Hong KJ. Interaction effect between prehospital hydration and initial cardiac rhythm in traumatic out-of-hospital cardiac arrest: a nationwide observational study. Signa Vitae. 2024;20(2):27-37.	Wrong hydration measure

149	Kleiner SM. Water: an essential but overlooked nutrient. J Am Diet Assoc. 1999;99(2):200-6.	No relevant included studies
150	Kohno K, Umemoto N, Tsutsumi R, Kawakami A, Sakaue H, Tamura K, et al. Diaper-zero program (prompted voiding care) improves diaper use in nursing home residents. Journal of Medical Investigation. 2023;70(1.2):221-5.	Not hospital or medical setting
151	Koopmans RT, Ekkerink JL, Sirre LG, Verkuylen MM, van den Hoogen HJ, van Weel C. [Multi-infarct dementia in nursing home patients; more comorbidity and shorter life expectancy than in Alzheimer's disease]. Nederlands Tijdschrift voor Geneeskunde. 1992;136(45):2223-7.	No hydration measure
152	Krishna BV, Patil A, Nadgir SD, Chandrasekhar MR. Incidence of Vibrio cholerae serogroup O139 infection with low virulence in Hubli, Karnataka (India). Indian Journal of Pathology & Microbiology. 2003;46(1):142-4.	Wrong hydration measure
153	Kristensen PK, Hjelholt TJ, Madsen M, Pedersen AB. Current Trends in Comorbidity Prevalence and Associated Mortality in a Population-Based Cohort of Hip Fracture Patients in Denmark. Clinical Epidemiology. 2023;15:839-53.	No hydration measure
154	Kurita Y, Suzuki K, Yagi S, Hasegawa S, Sato T, Hosono K, et al. Pre-emptive hydration with lactated Ringer's solution could reduce the incidence of post-endoscopic retrograde cholangiopancreatography pancreatitis in at-risk patients: Propensity score-matched analysis. Journal of Hepato-Biliary-Pancreatic Sciences. 2023;30(6):777-83.	No hydration measure
155	Kyne L, Merry C, O'Connell B, Kelly A, Keane C, O'Neill D. Factors associated with prolonged symptoms and severe disease due to Clostridium difficile. Age & Ageing. 1999;28(2):107-13.	No hydration measure
156	Lange-Asschenfeldt C, Kojda G, Cordes J, Hellen F, Gillmann A, Grohmann R, et al. Epidemiology, symptoms, and treatment characteristics of hyponatremic psychiatric inpatients. Journal of Clinical Psychopharmacology. 2013;33(6):799-805.	Not hospital or medical setting
157	Langer G, Wan CS, Fink A, Schwingshackl L, Schoberer D. Nutritional interventions for preventing and treating pressure ulcers. Cochrane Database Syst Rev. 2024;2:CD003216.	Participants chosen for dehydration
158	Lebensmittel MRIBfEu. ErnÃ_hrung Ã_lterer Menschen in stationÃ_ren Einrichtungen (ErnSTES-Studie). National Verzehrsstudie II, Ergebnisbericht Teil 2. 2008.	Wrong study design
159	Lee DH, Lee BK, Song KH, Jung YH, Park JS, Lee SM, et al. Prevalence and risk factors for central diabetes insipidus in cardiac arrest survivor treated with targeted temperature management. American Journal of Emergency Medicine. 2016;34(8):1400-5.	Wrong hydration measure
160	Lee J, Lam L, Woo J, Kwok T. Lower fluid and fruits/vegetable intake in questionable dementia among older Hong Kong Chinese. Journal of Nutrition, Health & Aging. 2010;14(1):45-9.	Mean <65 years

161	Lee JH, Machtay M, Unger LD, Weinstein GS, Weber RS, Chalian AA, et al. Prophylactic gastrostomy tubes in patients undergoing intensive irradiation for cancer of the head and neck. Arch Otolaryngol Head Neck Surg. 1998;124(8):871-5.	Pre or post operative fluids
162	Lemming EW, Pitsi T. The Nordic Nutrition Recommendations 2022 - food consumption and nutrient intake in the adult population of the Nordic and Baltic countries. Food and Nutrition Research. 2022;66:8572.	Not hospital or medical setting
163	Lesnik A, Piko N, Zeleznik D, Bevc S. Dehydration of Older Patients in Institutional Care and the Home Environment. Research in Gerontological Nursing. 2017;10(6):260-6.	Wrong hydration measure
164	Li S, Xiao X, Zhang X. Hydration Status in Older Adults: Current Knowledge and Future Challenges. Nutrients. 2023;15(11) (no pagination).	Not hospital or medical setting
165	Li S, Xiao X, Zhang X. Hydration Status in Older Adults: Current Knowledge and Future Challenges. Nutrients. 2023;15(11):02.	Wrong study design
166	Li Y, Wang L, Sun J, Xie T, Fu J, Feng C, et al. Effects of Subcostal Anterior Quadratus Lumborum Block with and without Dexmedetomidine on Postoperative Rehabilitation in Patients Undergoing Laparoscopic Renal Surgery: A Prospective Double-Blinded Randomized Controlled Study. Drug Design, Development and Therapy. 2023;17:3281-93.	Wrong study design
167	Liamis G, Tsimihodimos V, Doumas M, Spyrou A, Bairaktari E, Elisaf M. Clinical and laboratory characteristics of hypernatraemia in an internal medicine clinic. Nephrology Dialysis Transplantation. 2008;23(1):136-43.	Mean <65 years
168	Liang CK, Chu CS, Hsu YH, Chou MY, Wang YC, Lin YT, et al. Effects of modified version of the Hospital Elder Life Program on post-discharge cognitive function and activities of daily living among older adults undergoing total knee arthroplasty. Archives of Gerontology & Geriatrics. 2021;93:104284.	No hydration measure
169	Lim KB, Lim SY, Hor JW, Krishnan H, Mortadza F, Lim JL, et al. Orthostatic hypotension in Parkinson's disease: Sit-to-stand vs. supine-to-stand protocol and clinical correlates. Parkinsonism and Related Disorders. 2024;123(no pagination).	No hydration measure
170	Lim Z, Ling N, Ho VWT, Vidhya N, Chen MZ, Wong BLL, et al. Delirium is significantly associated with hospital frailty risk score derived from administrative data. International Journal of Geriatric Psychiatry. 2023;38(1) (no pagination).	Inclusion unclear - no author reply
171	Lim Z, Ling N, Ho VWT, Vidhya N, Chen MZ, Wong BLL, et al. Delirium is significantly associated with hospital frailty risk score derived from administrative data. International Journal of Geriatric Psychiatry. 2023;38(1):e5872.	Wrong hydration measure

172	Lin KY, Chen PC, Chen SC, Huang YL, Chen HC. Evaluation of the effects of glucose on osmolal gap using freezing point depression and vapor pressure methods. Kaohsiung Journal of Medical Sciences. 2018;34(7):409-14.	Wrong hydration measure
173	Lin Y, Kong J, Tian T, Zhong X, Zhang S, Zhou H, et al. Identification of Novel Phenotypes Correlated with CKD: A Phenotype-Wide Association Study. International Journal of Medical Sciences. 2022;19(13):1920-8.	Inclusion unclear - no author reply
174	Lindeman RD, Romero LJ, Liang HC, Baumgartner RN, Koehler KM, Garry PJ. Do Elderly Persons Need to Be Encouraged to Drink More Fluids? The Journals of Gerontology: Series A. 2000;55(7):M361-M5.	Wrong hydration measure
175	Lindner G, Kneidinger N, Holzinger U, Druml W, Schwarz C. Tonicity balance in patients with hypernatremia acquired in the intensive care unit. Am J Kidney Dis. 2009;54(4):674-9.	Not hospital or medical setting
176	Lindroos E, Saarela RK, Soini H, Muurinen S, Suominen MH, Pitkala KH. Caregiver-reported swallowing difficulties, malnutrition, and mortality among older people in assisted living facilities. Journal of Nutrition, Health & Aging. 2014;18(7):718-22.	Mean <65 years
177	Liu C, Bhat S, Sharma P, Yuan L, O'Grady G, Bissett I. Risk factors for readmission with dehydration after ileostomy formation: A systematic review and meta-analysis. Colorectal Disease. 2021;23(5):1071-82.	Not hospital or medical setting
178	Liu HZ, Wu GH. Prevalence of malnutrition in general surgical patients and analysis of the relation between nutritional status and prognosis. 2004(H034239).	Wrong study design
179	Liu Q, Donoso Brown E, Johnson S, Moss-Corcoran A. Providing Interest-based Activity Choices in Acute Care to Prevent Delirium: A Multicomponent Quality Improvement Project for a Hospital Elder Life Program (HELP). Epidemiology. 2022;70(SUPPL 1):S248.	No hydration measure
180	Lo JA, Kim JS, Jo MJ, Cho EJ, Ahn SY, Ko GJ, et al. Impact of water consumption on renal function in the general population: a cross-sectional analysis of KNHANES data (2008-2017). Clinical and Experimental Nephrology. 2021;25(4):376-84.	No hydration measure
181	Loda I, D'Angelo E, Marzetti E, Kerminen H. Prevention, Assessment, and Management of Malnutrition in Older Adults with Early Stages of Cognitive Disorders. Nutrients. 2024;16(11) (no pagination).	Wrong hydration measure
182	Lopes MMGD, Sousa IM, Queiroz SA, Bezerra MRO, Gonzalez MC, Fayh APT. Bioelectrical impedance vector analysis is different according to the comorbidity burden in post-acute myocardial infarction. Nutrition in Clinical Practice. 2024;39(2):450-8.	Wrong study design
183	Lu X, Zhen L, Qin F, Bai X, Wang L, Liu H, et al. Application value of short-term prehabilitation in elderly patients undergoing gastrointestinal tumor surgery. [Chinese]. Chinese Journal of Practical Nursing. 2023;39(11):801-6.	No hydration measure

184	Luger E, Dorner TE, Haider S, Kapan A, Lackinger C, Schindler K. Effects of a Home-Based and Volunteer-Administered Physical Training, Nutritional, and Social Support Program on Malnutrition and Frailty in Older Persons: A Randomized Controlled Trial. J Am Med Dir Assoc. 2016;17(7):671.e9-.e16.	No hydration measure
185	Ļwik MR, Westenbrink S, Hulshof KF, Kistemaker C, Hermus RJ. Nutrition and aging: dietary intake of apparently healthy elderly (Dutch Nutrition Surveillance System). J Am Coll Nutr. 1989;8(4):347-56.	Not hospital or medical setting
186	Ma W, Chen J, Wang W, Zhou Y, Yu S. [Analysis of prevalence and risk factors of hyperuricemia in subjects undergoing routine physical examinations in Guangzhou]. [Chinese]. Nan Fang Yi Ke Da Xue Xue Bao = Journal of Southern Medical University. 2012;32(12):1812-5.	Mean <65 years
187	Mack GW, Weseman CA, Langhans GW, Scherzer H, Gillen CM, Nadel ER. Body fluid balance in dehydrated healthy older men: thirst and renal osmoregulation. J Appl Physiol (1985). 1994;76(4):1615-23.	Not hospital or medical setting
188	Magnerou AM, Doumbe J, Lamou EGB, Massi DG, Tegueu CK. Prevalence and associated factors in post-stroke dysphagia. Journal of the Neurological Sciences. 2023;Conference: World Congress of Neurology(WCN 2023 . Montreal Canada. 455 Supplement).	Wrong hydration measure
189	Mandala C, Veronese N, Dominguez LJ, Candore G, Accardi G, Smith L, et al. Use of bioelectrical impedance analysis in centenarians: a systematic review. Aging Clinical and Experimental Research. 2023;35(1):01-Jul.	Wrong study design
190	Mannesse CK, Jansen PA, Van Marum RJ, Sival RC, Kok RM, Haffmans PM, et al. Characteristics, prevalence, risk factors, and underlying mechanism of hyponatremia in elderly patients treated with antidepressants: a cross-sectional study. Maturitas. 2013;76(4):357-63.	Mean <65 years
191	Mannesse CK, Jansen PAF, Van Marum RJ, Sival RC, Kok R, Judith Haffmans PM, et al. Hyponatremia in elderly patients treated with antidepressant drugs: A cross-sectional study on prevalence and clinical characteristics. European Geriatric Medicine. 2010;1:S82.	Not hospital or medical setting
192	Mao E, Li M, Cui Y, Chen S. Prevalence and risk factors associated with dehydration of patients with dysphagia in eastern China: A cross-sectional study. International journal of nursing practice. 2024:e13236.	Mean <65 years
193	Marcomini I, Pisoni L, Mellino A, Labaran R, Milani L. Evaluation of Delirium Among Elders in the Emergency Department: A Cross-Sectional Study. Dccn. 2024;43(3):130-5.	Wrong hydration measure
194	Marion TJ, Joel C, Florence CP, Jacques P, Antoine R, Laure CB. Impact of the COVID-19 pandemic on unplanned hospitalizations for geriatric syndromes in France from January to October 2020. European Geriatric Medicine. 2021;12(SUPPL 1):S160.	Wrong hydration measure
195	Mariz J, Santos NC, Afonso H, Rodrigues P, Faria A, Sousa N, et al. Risk and clinical-outcome indicators of delirium in an emergency department intermediate care unit (EDIMCU): an observational prospective study. BMC Emergency Medicine. 2013;13:2.	Mean <65 years

196	Marshall KA, Burson R, Gall K, Saunders MM. Hospital Admissions for Malnutrition and Dehydration in Patients With Dementia. Home Healthcare Now. 2016;34(1):32-7.	No hydration measure
197	Martin A, Arreola V, Ortega O, Vinas P, Nascimento W, Costa A, et al. Prevalence of Oropharyngeal Dysphagia and Malnutrition in Patients with Covid-19 at a General Hospital During Spring 2020 Pandemics. Dysphagia. 2023;38(Supplement 1):S51.	No hydration measure
198	Masot O, Lavedan A, Nuin C, Escobar-Bravo MA, Miranda J, Botigue T. Risk factors associated with dehydration in older people living in nursing homes: Scoping review. International Journal of Nursing Studies. 2018;82:90-8.	Not hospital or medical setting
199	Mathews KP. Causes and outcomes of severe hyponatremia in elderly hospitalized patients in a large tertiary care Indian teaching hospital. Journal of the American Geriatrics Society. 2015;63:S183.	No hydration measure
200	Mayo MAB, Izquierdo JQI. Ingestion of liquids and hydration in non-institutionalized older people in a municipality of Valencia (SPAIN). [Spanish]. Revista Espanola de Nutricion Comunitaria. 2019;25(2):54-61.	Not hospital or medical setting
201	Mboringong AB, Ngomtcho SC, Armel E, Linda E, Luc D, Patricia M, et al. Epidemiological description and analysis of factors associated with cholera morbidity and mortality in Cameroon from 2018 to 2023. medRxiv. 2024;22.	Mean <65 years
202	Megarbane B, Marsanne C, Meas T, Medeau V, Guillausseau PJ, Baud FJ. Acute lower limb ischemia is a frequent complication of severe diabetic hyperosmolarity. Diabetes & Metabolism. 2007;33(2):148-52.	Participants chosen for dehydration
203	Meiring PJ, Joubert G. Constipation in elderly patients attending a polyclinic. South African Medical Journal Suid-Afrikaanse Tydskrif Vir Geneeskunde. 1998;88(7):888-90.	Not hospital or medical setting
204	Melucci AD, Aquina CT, Loria A, McDonald G, Ghaffar A, Ramsdale E, et al. Impact of Incident Hospital-Acquired Geriatric Syndromes on Shortterm Outcomes following Gastrointestinal Cancer Resection. Annals of Surgical Oncology. 2023;30(Supplement 1):S20.	No hydration measure
205	Melucci AD, Loria A, Aquina CT, McDonald G, Schymura MJ, Schiralli MP, et al. New Onset Geriatric Syndromes and One-year Outcomes Following Elective Gastrointestinal Cancer Surgery. Ann Surg. 2024;279(5):781-8.	Wrong hydration measure
206	Mentes J. Oral hydration in older adults: greater awareness is needed in preventing, recognizing, and treating dehydration. Am J Nurs. 2006;106(6):40-9; quiz 50.	No hydration measure
207	Mentes JC. A typology of oral hydration problems exhibited by frail nursing home residents. Journal of gerontological nursing. 2006;32(1):13-9; quiz 20.	Not hospital or medical setting
208	Mihaescu A, Bucuras P, Dragota R, Chisavu L, Balan A, Marc L, et al. The prevalence of contrast induced acute kidney injury in patients with peripheral arterial disease treated by endovascular procedures. Nephrology Dialysis Transplantation. 2019;34 (Supplement 1):a441.	IV fluids

209	Molaschi M, Ponzetto M, Massaia M, Villa L, Scarafiotti C, Ferrario E. Hypernatremic dehydration in the elderly on admission to hospital. Journal of Nutrition, Health & Aging. 1997;1(3):156-60.	Participants chosen for dehydration
210	Mudge AM, McRae P, Banks M, Blackberry I, Barrimore S, Endacott J, et al. Effect of a Ward-Based Program on Hospital-Associated Complications and Length of Stay for Older Inpatients: The Cluster Randomized CHERISH Trial. JAMA Internal Medicine. 2022;182(3):274-82.	Wrong hydration measure
211	Mukand JA, Cai C, Zielinski A, Danish M, Berman J. The effects of dehydration on rehabilitation outcomes of elderly orthopedic patients. Arch Phys Med Rehabil. 2003;84(1):58-61.	Wrong hydration measure
212	Mullen M, Koay WJ, Duggan J. Reviewing time-to-surgery for acute hip fractures in older adults to develop 'SipTilSend' policies. Age and Ageing. 2023;52(Supplement 3):iii47.	Pre or post operative fluids
213	Munoz EC, Patino AO, Marin DJ, Cadavid LM, Ortega Quiroga L, Gonzalez Lopera D, et al. Immediate Impact on Tear Osmolarity Following the Application of Different Ocular Lubricants in Patients Experiencing Moderate Dry Eye Symptoms. Cornea. 2024;43(8):1040-3.	Wrong hydration measure
214	Nakagawa K, Yoshimi K, Yoshizawa A, Aritaki K, Yamaguchi K, Nakane A, et al. The Safety of Oral Rehydration Solution Jelly for Water and Electrolyte Intake in Patients with Dysphagia. Therapeutics and Clinical Risk Management. 2023;19:219-27.	Wrong hydration measure
215	Nardocci M, Polsky JY, Moubarac J-C. Consumption of ultra-processed foods is associated with obesity, diabetes and hypertension in Canadian adults. Canadian journal of public health = Revue canadienne de sante publique. 2021;112(3):421-9.	Wrong hydration measure
216	Nasiri N, Rahmati S, Etminan A, Sharifi H, Bazrafshan A, Karamouzian M, et al. Kidney Complications of COVID-19: A Systematic Review and Meta-Analysis. Journal of Research in Health Sciences. 2021;21(1):e00503.	Wrong study design
217	Natsume O. A clinical investigation of nocturnal polyuria in patients with nocturia: a diurnal variation in arginine vasopressin secretion and its relevance to mean blood pressure. Journal of Urology. 2006;176(2):660-4.	Not hospital or medical setting
218	Nieves-Anaya I, Vargas MB, Mayorga H, Garcia OP, Colin-Ramirez E, Atilano-Carsi X. Comparison of nutritional and hydration status in patients undergoing twice and thrice-weekly hemodialysis: a silent drama in developing countries. International Urology & Nephrology. 2021;53(3):571-81.	Mean <65 years
219	Nkonde C, Bell B, Tait A, Tan G, El-Zebdeh H, Yoshimatsu Y, et al. The Prevalence of Oral Frailty and Its Association with Dysphagia, Frailty and Formal Care Needs. Age and Ageing. 2023;52(Supplement 1):i11-i2.	Wrong hydration measure
220	Nyborg G, Brekke M, Straand J, Gjelstad S, Romoren M. Potentially inappropriate medication use in nursing homes: an observational study using the NORGEP-NH critria. BMC Geriatrics. 2017;17(1):220.	Not hospital or medical setting

221	Obata K, Naito H, Yakushiji H, Obara T, Ono K, Nojima T, et al. Incidence and characteristics of medical emergencies related to dental treatment: a retrospective single-center study. Acute Medicine and Surgery. 2021;8(1):e651.	Wrong hydration measure
222	Oh H, Seo W. Age differences in fluid balance and serum Na+ and K+ levels after nasogastric tube feeding in stroke patients: elderly vs nonelderly. Jpen: Journal of Parenteral & Enteral Nutrition. 2006;30(4):321-30.	Participant overlap with another study
223	Okamoto A, Kanda Y, Kimura SI, Oyake T, Tamura K, from the Japan Febrile Neutropenia Study G. Predictive and risk factor analysis for bloodstream infection in high-risk hematological patients with febrile neutropenia: post-hoc analysis from a prospective, large-scale clinical study. International Journal of Hematology. 2021;114(4):472-82.	Mean <65 years
224	Oliver CM, Warnakulasuriya S, McGuckin D, Singleton G, Martin P, Santos C, et al. Delivery of drinking, eating and mobilising (DrEaMing) and its association with length of hospital stay after major noncardiac surgery: observational cohort study. Br J Anaesth. 2022;129(1):114-26.	No hydration measure
225	Olszewska M, Schwermer K, Hoppe K, Misian M, Baum E, Pawlaczyk K, et al. Overhydration as a modifiable cardiovascular and all-cause mortality risk factor in hemodialysis patients. Hemodialysis International. 2017;21 (1):A24.	Dialysis
226	Ormseth CH, Lahue SC, Oldham MA, Josephson SA, Whitaker E, Douglas VC. Predisposing and Precipitating Factors Associated with Delirium: A Systematic Review. JAMA Network Open. 2023;6(1):E2249950.	Wrong study design
227	Ouertani I, Allouche E, Omrane M, Boudiche F, Bazdeh L, Ben Hamida F, et al. POS-010 THE HEART-KIDNEY CROSS TALK IN CARDIAC INTENSIVE CARE UNIT. Kidney International Reports. 2021;6(4 Supplement):S4.	Wrong hydration measure
228	Palevsky PM, Bhagrath R, Greenberg A. Hypernatremia in hospitalized patients. Ann Intern Med. 1996;124(2):197-203.	Wrong hydration measure
229	Palmer SC, Wong G, Iff S, Yang J, Jayaswal V, Craig JC, et al. Fluid intake and all-cause mortality, cardiovascular mortality and kidney function: a population-based longitudinal cohort study. Nephrology Dialysis Transplantation. 2014;29(7):1377-84.	Not hospital or medical setting
230	Pandey S, Kaushik S, Chandna V. Study of Incidence and Management of Catheter Associated Urinary Tract Infection. International Journal of Academic Medicine and Pharmacy. 2023;5(5):1610-6.	No hydration measure
231	Paquette IM, Solan P, Rafferty JF, Ferguson MA, Davis BR. Readmission for Dehydration or Renal Failure After Ileostomy Creation. Diseases of the Colon & Rectum. 2013;56(8):974-9.	Pre or post operative fluids
232	Park J. Nocturia, post-void residual, and fluid and caffeine intake in older Korean men living in rural areas. International Journal of Urology. 2017;24 (Supplement 1):65-6.	Not hospital or medical setting

233	Parkinson E, Hooper L, Fynn J, Wilsher SH, Oladosu T, Poland F, et al. Low-intake dehydration prevalence in non-hospitalised older adults: Systematic review and meta-analysis. Clin Nutr. 2023;42(8):1510-20.	Not hospital or medical setting
234	Pasman HR, Onwuteaka-Philipsen BD, Kriegsman DM, Ooms ME, Ribbe MW, van der Wal G. Discomfort in nursing home patients with severe dementia in whom artificial nutrition and hydration is forgone. Archives of Internal Medicine. 2005;165(15):1729-35.	Not hospital or medical setting
235	Paterna S, Gaspare P, Fasullo S, Sarullo FM, Di Pasquale P. Normal-sodium diet compared with low-sodium diet in compensated congestive heart failure: is sodium an old enemy or a new friend? Clinical Science. 2008;114(3):221-30.	No hydration measure
236	Patricia D, Sheil O, Roche E. Reported hydration intake of older patients in an emergency department. Age and Ageing. 2023;52(Supplement 3):iii17.	Wrong hydration measure
237	Paulis SJC, Everink IHJ, Halfens RJG, Lohrmann C, Schols J. Prevalence and Risk Factors of Dehydration Among Nursing Home Residents: A Systematic Review. Journal of the American Medical Directors Association. 2018;19(8):646-57.	Not hospital or medical setting
238	Peng Y, Wei H. Role of recombinant human brain natriuretic peptide combined with sodium nitroprusside in improving quality of life and cardiac function in patients with acute heart failure. Experimental and Therapeutic Medicine. 2020;20(1):261-8.	Wrong hydration measure
239	Phillips PA, Bretherton M, Johnston CI, Gray L. Reduced osmotic thirst in healthy elderly men. Am J Physiol. 1991;261(1 Pt 2):R166-71.	Not hospital or medical setting
240	Phillips PA, Bretherton M, Risvanis J, Casley D, Johnston C, Gray L. Effects of drinking on thirst and vasopressin in dehydrated elderly men. Am J Physiol. 1993;264(5 Pt 2):R877-81.	Not hospital or medical setting
241	Phillips PA, Jonhnston CI, Gray L. Thirst and fluid intake in the elderly. Thirst: physiological and psychological aspects ILSI Human Nutrition. 1991:403-11.	Mean <65 years
242	Phillips PA, Rolls BJ, Ledingham JG, Morton JJ. Body fluid changes, thirst and drinking in man during free access to water. Physiol Behav. 1984;33(3):357-63.	No hydration measure
243	Pioli MR, Couto RM, Francisco JA, Antoniassi DQ, de Souza CR, de Olivio MY, et al. Effectiveness of Oral Hydration in Preventing Contrast-Induced Nephropathy in Individuals Undergoing Elective Coronary Interventions. Arquivos Brasileiros de Cardiologia. 2023;120(2) (no pagination).	No hydration measure
244	Piquet MA, Ozsahin M, Larpin I, Zouhair A, Coti P, Monney M, et al. Early nutritional intervention in oropharyngeal cancer patients undergoing radiotherapy. Support Care Cancer. 2002;10(6):502-4.	Pre or post operative fluids
245	Puga AM, Partearroyo T, Varela-Moreiras G. Hydration status, drug interactions, and determinants in a Spanish elderly population: a pilot study. Journal of Physiology & Biochemistry. 2018;74(1):139-51.	Not hospital or medical setting

246	Pundi K, Perino AC, Fan J, Din N, Szummer K, Heidenreich P, et al. Association of CHA<inf>2</inf>DS<inf>2</inf>-VASc and HAS-BLED to frailty and frail outcomes: From the TREAT-AF study. American Heart Journal. 2023;261:85-94.	Wrong hydration measure
247	Quandt SA, Savoca MR, Leng X, Chen H, Bell RA, Gilbert GH, et al. Dry mouth and dietary quality in older adults in north Carolina. Journal of the American Geriatrics Society. 2011;59(3):439-45.	Not hospital or medical setting
248	Radulescu D, Cuiban E, David C, Garneata L, Turcu FL, Bogeanu C, et al. Urosepsis and hypovolemia the main causes of AKI in nursing home residents. Nephrology Dialysis Transplantation. 2024;39(Supplement 1):i1871-i3.	Inclusion unclear - no author reply
249	Rahayu RA, Boedhi-Darmojo R. Incidence and cause of acute confusion in elderly patients. Medical Journal of Indonesia. 2002;11(1):30-5.	No hydration measure
250	Rahayu RA, Boedhi-Darmojo R. Incidence and cause of acute confusion in elderly patients. Medical Journal of Indonesia. 2002;11(1):30-5.	Wrong study design
251	Reed PS, Zimmerman S, Sloane PD, Williams CS, Boustani M. Characteristics associated with low food and fluid intake in long-term care residents with dementia. Gerontologist. 2005;45(SPEC. ISS. 1):74-80.	Not hospital or medical setting
252	Ribeiro RSV, da Rosa MI, Bozzetti MC. Malnutrition and associated variables in an elderly population of Criciuma, SC. [Portuguese]. Revista Da Associacao Medica Brasileira. 2011;57(1):56-61.	Not hospital or medical setting
253	Robertson GL. Abnormalities of thirst regulation. Kidney Int. 1984;25(2):460-9.	No hydration measure
254	Robinson HG, Hermann J. Low-Income Older Adults' Use of Food Pantries as a Way to Cope with Food Insecurity. 2018(10686413):79.	Not hospital or medical setting
255	RodrÃ_guez N, HernÃ¡ndez R, Herrera H, Barbosa J, HernÃ¡ndez-Valera Y. Nutritional status of institutionalized Venezuelan elderly. InvestigaciÃ_n ClÃ_nica. 2005;46(3):219-28.	Not hospital or medical setting
256	Roe B, Flanagan L, Maden M. Systematic review of systematic reviews for the management of urinary incontinence and promotion of continence using conservative behavioural approaches in older people in care homes. Journal of Advanced Nursing. 2015;71(7):1464-83.	Not hospital or medical setting
257	Rosager EV, Helto ALK, Fox Maule CU, Friis-Hansen L, Petersen J, Nielsen FE, et al. Incidence and Characteristics of the Hyperosmolar Hyperglycemic State: A Danish Cohort Study. Diabetes Care. 2024;47(2):272-9.	Wrong hydration measure
258	Roser P, Mende KC, Dimitriadis GK, Mader MMD, Aberle J, Flitsch J, et al. The Potential of Self-Assessment and Associated Factors for Delayed Symptomatic Hyponatremia Following Transsphenoidal Surgery: A Single Center Experience. Journal of Clinical Medicine. 2023;12(1) (no pagination).	No hydration measure

259	Rosinger AY, John JD, Murdock KW. Ad libitum dehydration is associated with poorer performance on a sustained attention task but not other measures of cognitive performance among middle-to-older aged community-dwelling adults: A short-term longitudinal study. American journal of human biology : the official journal of the Human Biology Council. 2024;36(6):e24051.	Not hospital or medical setting
260	Roxanas M, Hibbert E, Field M. Venlafaxine hyponatraemia: incidence, mechanism and management. Australian & New Zealand Journal of Psychiatry. 2007;41(5):411-8.	Treatment alters sodium etc.
261	Saeki S, Watanabe N, Iida R, Kashiwazaki M, Itoh S, Ogawa S, et al. [A study of post-operative delirium in elderly patients]. Masui - Japanese Journal of Anesthesiology. 1998;47(3):290-9.	Unable to translate
262	Sailer CO, Winzeler B, Nigro N, Suter-Widmer I, Arici B, Bally M, et al. Characteristics and outcomes of patients with profound hyponatraemia due to primary polydipsia. Clinical Endocrinology. 2017;87(5):492-9.	Mean <65 years
263	Saito Y, Sakamoto T, Kobayashi M, Takekuma Y, Higuchi I, Okamoto K, et al. Evaluation of Prediabetes in Cisplatin-induced Nephrotoxicity in the Short Hydration Method: A Subgroup Analysis. In Vivo. 2024;38(2):800-6.	No hydration measure
264	Salama H, Omer MH, Shafqat A, Binahmed A, Alghamdi GM, Saeed M, et al. Avoidable emergency department visits among palliative care cancer patients: novel insights from Saudi Arabia and the Middle East. BMC Palliative Care. 2024;23(1) (no pagination).	End of life care
265	Sampson EL, Candy B, Jones L. Enteral tube feeding for older people with advanced dementia. Cochrane Database of Systematic Reviews. 2009(2).	Wrong study design
266	Sasaki CT, Leder SB. A typology of CAPS oral hydration problems exhibited by frail nursing home residents. Dysphagia. 2007;22(1):77.	Not hospital or medical setting
267	Sato N, Uno S, Kurita Y, Kim S. Efficacy and safety of intravenous OPC-61815 compared with oral tolvaptan in patients with congestive heart failure. ESC heart failure. 2022;9(5):3275-86.	Wrong hydration measure
268	Sayeh W, Farrow D, Jay B, Dhoop SM, Chinnam S, Rahyel AE, et al. Preventive Measures to Decrease the Risk of Post-Ercp Pancreatitis: A Systemic Review and Meta-Analysis. Gastroenterology. 2023;164(6 Supplement):S-381.	Wrong study design
269	Schaefer SM, Kaiser A, Behrendt I, Eichner G, Fasshauer M. Association of Alcohol Types, Coffee, and Tea Intake with Risk of Dementia: Prospective Cohort Study of UK Biobank Participants. Brain Sciences. 2022;12(3):360.	Wrong hydration measure
270	Schutz A, Eichinger W, Breuer M, Gansera B, Kemkes BM. Acute mesenteric ischemia after open heart surgery. Angiology. 1998;49(4):267-73.	Pre or post operative fluids

271	Scolapio JS, Spangler PR, Romano MM, McLaughlin MP, Salassa JR. Prophylactic placement of gastrostomy feeding tubes before radiotherapy in patients with head and neck cancer: is it worthwhile? J Clin Gastroenterol. 2001;33(3):215-7.	Pre or post operative fluids
272	Scorza C, Ferraz HB, Scorza FA. Association between water and fiber intake, constipation and levodopa dose in patients with Parkinson's disease. Clinical Nutrition ESPEN. 2023;Conference: ESPEN 2023. Lyon France. 58:733.	Inclusion unclear - no author reply
273	Serra-Prat M, Lorenzo I, Martinez J, Palomera E, Pleguezuelos E, Ferrer P. Relationship between Hydration Status and Muscle Catabolism in the Aged Population: A Cross-Sectional Study. Nutrients. 2023;15(22):08.	Not hospital or medical setting
274	Shemiesa RS, Abdelsalama M, Elnagara SS, Mohameda AH, Sayed-Ahmeda N, Tawfika M. Characteristics, risk factors and outcomes of community-acquired acute kidney injury in the elderly: a prospective tertiary hospital study, Egypt. African Health Sciences. 2022;22(2):350-61.	Wrong hydration measure
275	Silva M, Rodrigues S, Correia D, Torres D, Lopes C, Severo M. EATING IN OR OUT OF HOME IN THE PORTUGUESE POPULATION: ARE THERE DIFFERENCES IN DIETARY INTAKE? BMJ Nutrition, Prevention and Health. 2022;5(Supplement 1):A7.	Wrong hydration measure
276	Siqueira JH, Santana NMT, Pereira TSS, Moreira AD, Bensenor IM, Barreto SM, et al. Consumption of alcoholic and non-alcoholic beverages: ELSA-Brasil results. Ciencia & Saude Coletiva. 2021;26(suppl 2):3825-37.	Not hospital or medical setting
277	Siritaweesuk N, Satirapoj B, Chaiprasert A, Nata N, Kajanakul I, Supasyndh O. Protective effects of oral L-arginine supplement on acute kidney injury in high risk patients after intravenous contrast media injection: A randomized controlled trial. Nephrology. 2020;25 (SUPPL 4):32-3.	No hydration measure
278	Slattery ML, Caan BJ, Anderson KE, Potter JD. Intake of fluids and methylxanthine-containing beverages: association with colon cancer. International Journal of Cancer. 1999;81(2):199-204.	Not hospital or medical setting
279	Soos R, Bako C, Gyebrovszki A, Gordos M, Csala D, Adam Z, et al. Nutritional Habits of Hungarian Older Adults. Nutrients. 2024;16(8) (no pagination).	Inclusion unclear - no author reply
280	Soric T, Mavar M, Rumbak I. Metabolic Syndrome and Dietary Habits in Hospitalized Patients with Schizophrenia: A Cross-Sectional Study. Medicina. 2021;57(3):10.	Mean <65 years
281	Spangler PF, Risley TR, Bilyew DD. THE MANAGEMENT OF DEHYDRATION AND INCONTINENCE IN NONAMBULATORY GERIATRIC PATIENTS. Journal of Applied Behavior Analysis. 1984;17(3):397-401.	Not hospital or medical setting
282	Sreeram S, Mahesh E, Gurudev, Gireesh, Rajashekar, Pooja, et al. Clinico. Indian Journal of Nephrology. 2023;33(Supplement 1):S102.	Wrong hydration measure

283	Sri-On J, Thong-On K, Kredarunsooksree T, Paksopis T, Ruangsiri R. Prevalence and Risk Score for Hypertonic Dehydration Among Community-Dwelling Older Adults: an Analysis of the Bangkok Falls Study. Gerontology. 2023.	Not hospital or medical setting
284	Stachenfeld NS, DiPietro L, Nadel ER, Mack GW. Mechanism of attenuated thirst in aging: role of central volume receptors. Am J Physiol. 1997;272(1 Pt 2):R148-57.	Not hospital or medical setting
285	Stachenfeld NS, Mack GW, Takamata A, DiPietro L, Nadel ER. Thirst and fluid regulatory responses to hypertonicity in older adults. Am J Physiol. 1996;271(3 Pt 2):R757-65.	Not hospital or medical setting
286	Starace E, De Pasquale G, Morenghi E, Crippa C, Matteucci S, Pieri G, et al. Hospital Malnutrition in the Medicine and Neurology Departments: A Complex Challenge. Nutrients. 2023;15(24) (no pagination).	Inclusion unclear - no author reply
287	Stookey JD. High prevalence of plasma hypertonicity among community-dwelling older adults: results from NHANES III. Journal of the American Dietetic Association. 2005;105(8):1231-9.	Not hospital or medical setting
288	Suarez Carantona C, Escobar Cervantes C, Fabregate M, Lopez Rodriguez M, Bara Ledesma N, Soto Perez-Olivares J, et al. Oral Sodium Chloride in the Prevention of Contrast-Associated Acute Kidney Injury in Elderly Outpatients: The PNIC-Na Randomized Non-Inferiority Trial. Journal of Clinical Medicine. 2023;12(8) (no pagination).	Wrong hydration measure
289	Szabo M, Pleck AP, Soos SA, Keczer B, Varga B, Szell J. A preoperative ultrasound-based protocol for optimisation of fluid therapy to prevent early intraoperative hypotension: a randomised controlled study. Perioperative Medicine. 2023;12(1) (no pagination).	IV fluids
290	Takamata A, Ito T, Yaegashi K, Takamiya H, Maegawa Y, Itoh T, et al. Effect of an exercise-heat acclimation program on body fluid regulatory responses to dehydration in older men. Am J Physiol. 1999;277(4):R1041-50.	Not hospital or medical setting
291	Tamir YA, Tellem R, Cialic R, Barak O. Reducing mortality of at-risk elders: First results of a customized HELP program in the surgical division of a large Israeli tertiary hospital. European Geriatric Medicine. 2022;13(Supplement 1):S18.	No hydration measure
292	Tay CL, Myint PK, Mohazmi M, Soiza RL, Tan MP. Prevalence and documented causes of hyponatraemia among geriatric patients attending a primary care clinic. Medical Journal of Malaysia. 2019;74(2):121-7.	Wrong hydration measure
293	Taylor EN, Fung TT, Curhan GC. DASH-style diet associates with reduced risk for kidney stones. Journal of the American Society of Nephrology. 2009;20(10):2253-9.	Not hospital or medical setting
294	Thiel A, Nau R, Willers T, Ruther E, Prange HW. [Intensive care treatment of psychiatric patients]. Nervenarzt. 1994;65(3):183-90.	Mean <65 years
295	Thomas SD, White GH, Coates PS. Prevalence of falls and fractures in hyponatremic patients presenting to an emergency department. Clinical Chemistry. 2010;56(6):A74.	Participants chosen for dehydration

296	Tilleul J, Glacet-Bernard A, Coscas G, Soubrane G, Souied EH. [Underlying conditions associated with the occurrence of retinal vein occlusion]. Journal Francais d Opthalmologie. 2011;34(5):318-24.	No hydration measure
297	Toh V, Tee SP, Lee SH. Clinical characteristics and predictors of mortality in patients with melioidosis: the Kapit experience. Tropical Medicine & International Health. 2021;26(6):664-71.	Mean <65 years
298	Tomsen N, Bolivar-Prados M, Ortega O, Clave P. Therapeutic Effect on Swallowing Function and on Hydration Status of a New Liquid Gum-Based Thickener in Independently-Living Older Patients with Oropharyngeal Dysphagia. Nutrients. 2023;15(21) (no pagination).	Not hospital or medical setting
299	Townsend MK, Jura YH, Curhan GC, Resnick NM, Grodstein F. Fluid intake and risk of stress, urgency, and mixed urinary incontinence. American Journal of Obstetrics & Gynecology. 2011;205(1):73.e1-6.	Not hospital or medical setting
300	Trads M, Deutch SR, Pedersen PU. Supporting patients in reducing postoperative constipation: fundamental nursing care - a quasi-experimental study. Scandinavian Journal of Caring Sciences. 2018;32(2):824-32.	Inclusion unclear - no author reply
301	Tsai CH, Chang P, Sung YP. Related factors of incontinence and continence management strategies among the elderly of long-term care facilities in Taiwan. Neurourology and Urodynamics. 2018;37 (Supplement 5):S146.	Not hospital or medical setting
302	Tzoulis P, Bouloux PM. Inpatient hyponatraemia: adequacy of investigation and prevalence of endocrine causes. Clinical Medicine. 2015;15(1):20-Apr.	Treatment alters sodium etc.
303	Uhm SJ, Meszaros E, Hammonds K, Patel I, Herrington JD. Characterizing the impact of magnesium and potassium-supplemented hydration with cisplatin and the subsequent electrolyte replacement requirements. Journal of Oncology Pharmacy Practice. 2021;27(5):1125-31.	Wrong hydration measure
304	Uyterlinde W, Chen C, Nijkamp J, Obbink MG, Sonke JJ, Belderbos J, et al. Treatment adherence in concurrent chemoradiation in patients with locally advanced non-small cell lung carcinoma: results of daily intravenous prehydration. Radiotherapy & Oncology. 2014;110(3):488-92.	Mean <65 years
305	Vanderlee L, White CM, Kirkpatrick SI, Rynard VL, Jauregui A, Adams J, et al. Nonalcoholic and Alcoholic Beverage Intakes by Adults across 5 Upper-Middle- and High-Income Countries. Journal of Nutrition. 2021;151(1):140-51.	Mean <65 years
306	Vasudev A, Mohamed I, Jacques H, Nicolson P. A quality improvement project aimed toreduce the number of patients onbournville ward suffering withconstipation. Age and Ageing. 2021;50(SUPPL 1).	No hydration measure
307	Venturini C, Orlandoni P, Jukic Peladic N, Giorgini N, Cola C, Sparvoli D, et al. High prevalence of dysphagia among hospitalised elderly patients: Its impact on nutritional and functional status. Clinical Nutrition. 2013;32:S33.	Wrong hydration measure

308	Venturini C, Orlandoni P, Jukic Peladic N, Giorgini N, Cola C, Sparvoli D, et al. Prevalence of malnutrition among hospitalized geriatric patients and its management. Clinical Nutrition. 2013;32:S187.	Wrong hydration measure
309	Vinas P, Martin-Martinez A, Cera M, Riera SA, Escobar R, Clave P, et al. Characteristics and Therapeutic Needs of Older Patients with Oropharyngeal Dysphagia Admitted to a General Hospital. J Nutr Health Aging. 2023;27(11):996-1004.	Wrong hydration measure
310	Vinas P, Martin-Martinez A, Cera M, Riera SA, Escobar R, Clave P, et al. Characteristics and Therapeutic Needs of Older Patients with Oropharyngeal Dysphagia Admitted to a General Hospital. Journal of Nutrition, Health and Aging. 2023;27(11):996-1004.	Wrong hydration measure
311	Vu M, Sileanu FE, Aspinall SL, Niznik JD, Springer SP, Mor MK, et al. Antihypertensive Deprescribing in Older Adult Veterans at End of Life Admitted to Veteran Affairs Nursing Homes. Journal of the American Medical Directors Association. 2021;22(1):132-40.e5.	Wrong hydration measure
312	Wakefield BJ, Mentes J, Holman JE, Culp K. Postadmission dehydration: risk factors, indicators, and outcomes. Rehabilitation Nursing Journal. 2009;34(5):209-16.	Mean <65 years
313	Wakefield BJ, Mentes J, Holman JE, Culp K. Risk factors and outcomes associated with hospital admission for dehydration. Rehabil Nurs. 2008;33(6):233-41.	Wrong hydration measure
314	Walecka-Kapica EM, Kaczka A, Blonska A, Gasiorowska A. The impact of nutritional and lifestyle factors on the incidence of colon diverticulosis in different age groups. United European Gastroenterology Journal. 2020;8 (8 SUPPL):292-3.	No hydration measure
315	Wang HW, Jiang MY. Higher volume of water intake is associated with lower risk of albuminuria and chronic kidney disease. Medicine. 2021;100(20):e26009.	Mean <65 years
316	Wang LM, Tsai ST, Ho LT, Hu SC, Lee CH. Rhabdomyolysis in diabetic emergencies. Diabetes Research & Clinical Practice. 1994;26(3):209-14.	Participants chosen for dehydration
317	Warren JL, Bacon WE, Harris T, McBean AM, Foley DJ, Phillips C. The burden and outcomes associated with dehydration among US elderly, 1991. Am J Public Health. 1994;84(8):1265-9.	Wrong hydration measure
318	Watne LO, Torbergsen AC, Conroy S, Engedal K, Frihagen F, Hjorthaug GA, et al. The effect of a pre- and postoperative orthogeriatric service on cognitive function in patients with hip fracture: randomized controlled trial (Oslo Orthogeriatric Trial). BMC Med. 2014;12:63.	Wrong hydration measure
319	Weidner E, Hancke L, Nydahl P, Spies C, Lutz A. [Non-pharmacological Management of Postoperative Delirium]. Anasthesiol Intensivmed Notfallmed Schmerzther. 2023;58(9):494-512.	Wrong study design
320	Williams SW, Williams CS, Zimmerman S, Sloane PD, Preisser JS, Boustani M, et al. Characteristics associated with mobility limitation in long-term care residents with dementia. Gerontologist. 2005;45(SPEC. ISS. 1):62-7.	Not hospital or medical setting

321	Woo J, Lee J, Park SY. Delirium of elderly patients in the emergency room. European Neuropsychopharmacology. 2012;22:S386-S7.	No hydration measure
322	Woo J, Swaminathan R. Plasma osmolality in an elderly population. Journal of Medicine. 1991;22(2):69-75.	Not hospital or medical setting
323	Woodhouse R, Burton JK, Rana N, Pang YL, Lister JE, Siddiqi N. Interventions for preventing delirium in older people in institutional long-term care. Cochrane Database of Systematic Reviews. 2019;4:CD009537.	Wrong study design
324	Wu M, Jiang S, Lu X, Zhong Y, Song Y, Fan Z, et al. Aggressive hydration with lactated ringer solution in prevention of post-endoscopic retrograde cholangiopancreatography pancreatitis: A systematic review and meta-analysis. Medicine. 2021;100(16):e25598.	Wrong study design
325	Xia X, Zhang X, Xia H. A study of factors related to the incidence of cataract in patients with non-insulin dependent diabetes mellitus. Yen Ko Hsueh Pao [Eye Science]. 2001;17(3):180-2.	Mean <65 years
326	Xiao H, Barber J, Campbell ES. Economic burden of dehydration among hospitalized elderly patients. Am J Health Syst Pharm. 2004;61(23):2534-40.	Wrong hydration measure
327	Xue Y, Wang Q, Zhao H, Pan R, Xia Y, Wang H, et al. The efficacy and safety of modified ultraearly oral hydration for alleviating thirst in patients after thoracoscopic surgery: a prospective randomized controlled trial. BMC anesthesiol. 2024;24(1):105.	No hydration measure
328	Yagi S, Furukawa S, Miyake T, Yoshida O, Shiraishi K, Tange K, et al. Aging is Associated With Constipation in Japanese Patients With Ulcerative Colitis: A Post Hoc Analysis. Gerontology and Geriatric Medicine. 2023;9(no pagination).	No hydration measure
329	Yang L, Kemp L, Mirk A, Manns K. Evaluation of Empagliflozin Use in Older Adult Veterans. Journal of the American Geriatrics Society. 2023;71(Supplement 1):S353-S4.	Wrong hydration measure
330	Yoo JI, Choi H, Song SY, Park KS, Lee DH, Ha YC. Relationship between water intake and skeletal muscle mass in elderly Koreans: A nationwide population-based study. Nutrition. 2018;53:38-42.	Not hospital or medical setting
331	Yoshikawa D, Isobe S, Sato K, Ohashi T, Fujiwara Y, Ohyama H, et al. Importance of oral fluid intake after coronary computed tomography angiography: an observational study. European Journal of Radiology. 2011;77(1):118-22.	Pre or post operative fluids
332	Young J, Green J, Farrin A, Collinson M, Hartley S, Smith J, et al. A multicentre, pragmatic, cluster randomised, controlled feasibility trial of the POD system of care. Age Ageing. 2020;49(4):640-7.	No hydration measure
333	Yurtdas Depboylu G, Acar Tek N, Akbulut G, Gunel Z, Kamanli B. Functional Constipation in Elderly and Related Determinant Risk Factors: Malnutrition and Dietary Intake. Journal of the American Nutrition Association. 2022.	Wrong hydration measure

334	Zacchia M, Navarria A, Baughman S, Roux W, Malhotra S, Yuan G, et al. Pharmacokinetics of Setmelanotide in Individuals with Renal Impairment: Results from a Phase 1, Open-Label, Single-Dose Study. Nephrology Dialysis Transplantation. 2023;38(Supplement 1):i1133.	No hydration measure
335	Zadek F, Beatrice B, Mulazzani F, Bonenti C, Tardini F, Langer T, et al. Sodium alterations during continuous veno-venous renal replacement therapy. Intensive Care Medicine Experimental Conference: European Society of Intensive Care Medicine Annual Congress, ESICM. 2023;11(Supplement 1).	Mean <65 years
336	Zhang Y, Wang X, Sang X, Zhou Z, Dai G, Zhang X. Effect of Fluid Therapy in Early Morning on the Incidence of Post-Induction Hypotension During Non-Cardiac Surgery After Noon: A Single-Center Retrospective Study. Drug Des Devel Ther. 2024;18:1339-47.	No hydration measure
337	Zhou JH, Qi L, Wang J, Liu SX, Shi WH, Ye LL, et al. [Prediction model related to 6-year risk of frailty in older adults aged 65 years or above in China]. Chung Hua Liu Hsing Ping Hsueh Tsa Chih. 2024;45(6):809-16.	Not hospital or medical setting
338	Zhu Q, Gao P, Fu S, Wang H, Bai Y, Luo L, et al. Prognostic Value of Plasma NT-proBNP levels in Hospitalized Patients Older than 80 Years of Age in a Hospital in Beijing, China. Arquivos Brasileiros de Cardiologia. 2021;116(6):1027-36.	No hydration measure
339	Zizza CA, Ellison KJ, Wernette CM. Total water intakes of community-living middle-old and oldest-old adults. The journals of gerontology Series A, Biological sciences and medical sciences. 2009;64(4):481-6.	Not hospital or medical setting
340	Zylberman M, Couselo FAD. Incidence and mortality due to hyponatremia in patients with cancer. [Spanish]. Revista de Nefrologia, Dialisis y Trasplante. 2010;30(3):100-9.	Mean <65 years

Supplementary File 4. Characteristics of all 40 included studies

Summary: Twenty nine included studies were conducted in Europe (of which 8 in the UK), six in Asia, three in Australia or New Zealand and two in North America. Study designs were mixed, including two RCTs, 24 cohort studies, one case control, 12 cross-sectional studies and one diagnostic accuracy study. Mean age by study ranged from 65 to 86 years, and studies either restricted participants to older adults (using a lower age cut-off), or included a range of ages, but due to the setting (e.g. geriatric unit) or medical condition (e.g. stroke) mean age was ≥65 years. The number of participants ranged from 9 to 16,598, of which 17 to 89% were women. Participants included patients diagnosed with severe vertigo, stroke (including those with dysphagia post-stroke), myocardial infarction with cardiogenic shock, acute heart failure, deep vein thrombosis, decompensated liver cirrhosis, cardiorenal syndrome with or without sepsis, bacteraemic sepsis, hip fracture, neoplasms, COVID, cognitive decline, or requiring cardiac surgery, transvenous cardiac pacing or percutaneous coronary intervention. Others represented mixed groups of older adults in the emergency department, geriatric ward, medical ward or ICU.

Study ID, continent	Study design	Study dates	Population description	N with data on hydration status	Age in years, Mean (SD)*	Female (n, %)	Characteristics related to subgrouping**
Aoki 2007 (15), Asia	Cross sectional	Unclear	Patients admitted for severe vertigo	9 aged 65+ of 38 in total	72 (7) aged 65+	8, 89% aged 65+	None reported
Bech 2023 (16), Europe	Cohort	May-Nov 2022	Adults aged 65+ on geriatrics unit	114	Median 85.5, IQR 80 to 89	65, 57%	Renal status: 12% had eGFR <30<br>Diabetes: 19%<br>Comorbidities: 83% (4+ prescribed meds)<br>Dependency: 18% bed or chair bound, 47% not bed or chair bound, but don't go out
Betrosian 1999 (17), Europe	Cohort	Jan 1993 to Jan 1996	Patients with bacteraemic sepsis in ICU	157	69 (15)	93, 59%	Renal status: 31% had acute renal failure
Bhalla 2000 (18), Europe	Cohort	Nov 1998 to Nov 1999	Patients with diagnosed stroke	167	73 (12)	87, 52%	Diabetes: 10% had diabetes<br>Stroke: 100%<br>Dependency: 12% totally dependent
Bourdel-Marchasson 2004 (19), Europe	Cohort	Dec 1999 to May 2000	Adults aged 75+ on geriatric ward	436	85 (6)	321, 74%	Cognitive impairment: 35% (assessed using series of questions)<br>Diabetes: 17% had diabetic osmolarity

Buaprasert 2021 (20), Asia	Cross sectional	May to Jul 2017	Adults aged 65+ in ED	370	78 (8)	234, 63%	Ethnicity: All Thai (Asian)<br>Renal: 27% had moderate or severe renal disease<br>Cognitive impairment: 6% dementia, also 6-item cognitive impairment test<br>Diabetes: 22% had diabetes with end organ damage<br>Stroke: 17%<br>Comorbidities: CCI median 5, IQR 4-7<br>Dependency: 8% had complete dependence by ADL
Buoite Stella 2020 (21), Europe	Cohort	Aug 2017 to Jun 2018	Patients with diagnosis of acute ischemic stroke	119	69 (16)	56, 47%	Renal status: 0% (excluded chronic renal disease)<br>Diabetes: 22% had diabetes<br>Stroke: 100%<br>Dependency: 2% mRS >2
Claesson Lingehall 2023 (22), Europe	RCT	Apr 2019 to Jun 2020	Patients 65+ scheduled for cardiac surgery	195	73 (4)	53, 27%	Cognitive impairment: median MMSE 28, IQR 3<br>Diabetes: 22% had diabetes<br>Stroke: 9% cerebrovascular disease<br>Dependency: 93% fully independent (% dependent not reported)
Durakovic 1997 (23), Europe	Cohort	Jan 1983 to Dec 1993	Adults 65+ with acute myocardial infarction & cardiogenic shock	11	73 (5)	3, 27%	Renal status: 64% (assessment unclear)
El-Sharkawy 2015 (24), Europe	Cohort	Aug 2012 to Apr 2014	Adults aged 65+ with emergency admission	187	82 (7)	87, 47%	Renal status: 11% had AKI, measured eGFR<br>Cognitive impairment: Mean MMSE 24, SD 6<br>Delirium: 27%<br>Comorbidities: Median 4<br>Dependency: mean BI 15 (appears to be of 20)
El-Sharkawy 2020 (25), Europe	Cohort	May 2011 to Oct 2013	Patients aged 65+ with emergency admission to medical specialities	6632	NR	3469, 525	Renal status: 23% had AKI<br>Cognitive impairment: dementia in 6% of dehydrated, 2% euhydrated<br>Comorbidities: CCI median 1

Farhan 2019 (26), N. America	Cohort	Jun 2012 to May 2017	Patients undergoing Percutaneous coronary intervention (PCI)	1927	65.3 (13)	650, 34%	Renal status: 35% had CKD<br>Diabetes: 48% had diabetes
Fiaux 2015 (27), Europe	Cross sectional	May 2010 to Apr 2013	Patients aged 70+ with suspected deep vein thrombosis	144	82 (6)	93, 65%	Renal status: 28% (assessment unclear)
Fortes 2015 (28), Europe	Cross sectional	May-Nov 2011	Adults aged 60+ admitted to acute medical care or ED	130	78 (9)	71, 55%	Renal status: 0% (CKD stages 1-5 excluded)<br>Cognitive impairment: 0% (all provided informed consent)
Gou 2021 (29), Asia	Cohort	Feb 2020	Critically ill adults with SARS-CoV-2 on ICU	146	NR (63% aged 65+)	56, 38%	Renal status: 5% had AKI or chronic renal disease<br>Diabetes: 34% had diabetes<br>Stroke: 7% cerebrovascular disease
Jespersen 2023 (30), Europe	Cohort	May 2019 to Feb 2022	Older patients acutely admitted to medical ward	99	81 (8)	60, 61%	Renal status: 12% (eGFR <30)
Kafri 2013 (31), Europe	Cross sectional	Apr 2011 to Oct 2011	Stroke patients admitted within 48 hours of symptom onset	27	71 (11)	11, 41%	Cognitive impairment: 0% (all provided informed consent)<br>Stroke: 100%<br>Diabetes: assessed mean serum glucose<br>Dependency: 4% dependent, 11% semi-independent by pre-morbid Rankin scores
Khanimov 2020 (32), Asia	Cohort	Jan 2010 to Dec 2013	Patients discharged from internal medicine units	7698	72 (17)	3898, 50%	Renal status: 14% had chronic renal failure<br>Cognitive impairment: 4% had dementia<br>Diabetes: 27%<br>Stroke: 4% cerebrovascular disease<br>Comorbidities: Mean CCI close to 1
Lauriola 2018 (33), Europe	Case control	Jan 2015 to Mar 2017	Adults with or without cognitive decline, Geriatric Unit	1091 (571 cases, 520 controls)	79 (7)	549, 50%	Ethnicity: All Caucasian<br>Renal status: Mean eGFR 71 SD 18<br>Cognitive impairment: Mean MMSE 23, SD 7<br>Comorbidities: Mean CIRS 2.3, SD 1.5<br>Dependency: mean ADL 6 (SD 0, range 6-6), mean IADL 8 (SD 0, range 8-8)

McCrow 2016 (34), Anzac	Cohort	Jul 2013 to Nov 2014	Patients aged 60+ admitted to internal medical unit	44	81 (9)	24, 55%	Cognitive impairment: 61% cognitive impairment
Miller 2019 (35), Europe	Cohort	Mar-June 2017	Adult patients admitted with stroke	41 of 48	Mean 75, range 48-100	25, 50%	Stroke: 100%<br>Dependency: mean NIHSS score 7.6 (5-14 means mild to moderately severe stroke - lower is milder)
Munk 2021 (36), Europe	Diagnostic	Apr-May 2019	Patients aged 65+ years admitted to ED	90	Median 78, IQR 72 to 86	48, 53%	Renal status: 41% with eGFR 30 to <60 (<30 excluded)<br>Cognitive impairment: 0%<br>Diabetes: 16% had diabetes
Murray 2016 (37), Anzac	RCT	Nov 2009 to Feb 2013	Adults with swallowing problems post-stroke	14	79 (6)	4, 29%	Cognitive impairment: 36% (CI not defined)<br>Stroke: 100%
Nagae 2023 (38), Asia	Cohort	Oct 2019 to Mar 2022	Patients aged 65+ years admitted to geriatric ward	192	85 (6)	113, 59%	Renal function: measured eGFR<br>Cognitive impairment: 49% had dementia, MMSE median 20 IQR 8-25 in dehydrated, 23, 18-27 euhydrated.<br>Diabetes: 29% had diabetes<br>Comorbidities: CCI median 2, IQR 1-3<br>Dependency: Median BI 90 (IQR 65-100, minimal impairment), IADL median 5 (IQR 2-7)
Nielsen 2024 (39), Europe	Cohort	Oct 2018 to Apr 2021	Older adults acutely admitted to ED	42	79 (7)	27, 64%	Ethnicity: All Caucasian (others excluded)<br>Stroke: NR (31% CVD)<br>Comorbidities: 10% with CCI of 2+
Oh 2007 (40), Asia	Cross sectional	1997- 2001	Hospitalised stroke patients to be tube fed	85	68 (12)	47, 55%	Diabetes: 15% had diabetes<br>Stroke: 100%
Palmisano 2014 (41), Europe	Cross sectional	Sep 2007 to Aug 2012	Patients 65+ for urgent transvenous cardiac pacing in ED	79	82 (8)	47, 60%	Renal status: 5% had chronic renal failure<br>Diabetes: 4% had diabetes<br>Stroke: 6%

Pfortmueller 2014 (42, 43), Europe	Cross sectional	Jan 2002 to Dec 2012	Patients 65+ with decompensated liver cirrhosis in ED	54	69 (4)	13, 24%	Renal status: 37% had eGFR <60<br>Diabetes: 50%
Pliquett 2020 (44), Europe	Cohort	Unclear (pre 2020 over 4 years)	Cardiorenal syndrome (CRS) patients without sepsis in internal medicine	93	74 (13)	82, 48%	Renal status: 54% had CKD, 46% AKI<br>Diabetes: 66%
Sabanovic 2022 (45), Europe	Cohort	Mar 2018 to Aug 2020	Patients 65+ with hip fracture in geriatric unit	214	81 (8)	147, 69%	Cognitive impairment: 19% mild to mod impairment, 7% severe
Sanson 2021 (46), Europe	Cohort	Oct 2015 to Jul 2016	Patients 65+ admitted from ED to Internal Medicine	4613	NR	2675, 58%	Renal status: 45% had eGFR <60<br>Comorbidities: CCI median 7, IQR 5-8<br>Dependency: 20% dependent (Norton scale up to 9)
Shen 2017 (47), N. America	Cohort	2001-2008	Adults on ICU with calculable osmolarity	16598	65 (23)	7107, 43%	Diabetes: 19%<br>Stroke: 15% cerebral disease
Sjöstrand 2013 (43, 48), Europe	Cohort	Spring-summer 2010	Patients 75+ admitted to ED awaiting treatment	36	84 (6)	20, 56%	Renal status: 35% eGFR <60
Sokolski 2019 (49, 50), Europe	Cohort	2010-2 and 2016-7	Patients admitted with acute heart failure	338	68 (13)	80, 24%	None reported
Tellini 2005 (51), Europe	Cross sectional	Unclear (pre 2005, over 6 months)	Elderly patients with neoplasm on geriatric ward	15	79 (7)	8, 53%	None reported
Van Wijk 2023 (52), Europe	Cross sectional	Jan 2018 to Jul 2019	Ischemic stroke patients 50-75 years, some with oropharyngeal dysphagia	36	65 (7)	6, 17%	Ethnicity: 167 Caucasian, 1 Asian<br>Cognitive impairment: 0%<br>Diabetes: 0% (excluded)<br>Stroke: 100%<br>Dependency: 44% severe/extreme problems with self-care, 35% slight to moderate problems, dysphagia in some
Vivanti 2008 (53), Anzac	Cohort	May-Dec 2002	Older adults voluntarily admitted to geriatric rehab	43	78 (8)	28, 65%	Renal function: renal calculae reported<br>Diabetes: unclear (but results of correlation with dehydration reported)

Walsh 2012 (54), Europe	Cross sectional	Jul-Dec 2011	Older hospital patients on acute medical unit	99	77 (8)	55, 50%	Renal status: 0% (chronic renal disease excluded)
Wojszel 2020 (55), Europe	Cross sectional	7 months in 2014-2015	Older patients admitted to geriatric ward	358	median 82, IQR 78 to 86	274, 77%	Renal status: 53% chronic renal disease<br>Cognitive impairment: 34% had dementia<br>Diabetes: 30% had diabetes<br>Stroke: 15% stroke or TIA<br>Comorbidities: 59% multimorbidity, median no of meds 7, IQR 5-9<br>Dependency: 25% noted as being severely frail
Zanetti 2022 (56), Europe	Cohort	Jan – Dec 2019	Older adults acutely admitted to geriatric Unit	529	85 (7)	326, 62%	Renal status: 28% kidney failure<br>Cognitive impairment: 35% had dementia<br>Diabetes: 43% had diabetes<br>Stroke: 11%<br>Comorbidities: Cumulative Illness Rating Scale mean 4.6, SD 2<br>Dependency: IADL mean 3.6 (0 is dependent, 8 independent)

* Data reported as mean (standard deviation) unless otherwise stated.

**These include any reporting on the following characteristics: Ethnicity, renal status, cognitive impairment, Diabetes, stroke, delirium, comorbidities, dependency

ADL: Activities of daily living, CCI: Charlson Comorbidity Index, CIRS: Cumulative illness rating scale

EGFR: estimated glomerular filtration rate, ICU: intensive care unit, IADL: Instrumental activities of daily living, IQR: Interquartile range, ED: emergency department, RCT: randomised controlled trial

Supplementary File 5. Table of hydration data of the 40 included studies

Summary: Of the forty included studies:

- 12 assessed directly measured osmolality in mOsm/kg (assessed by freezing point depression) (18, 20, 22, 24, 28, 31, 36, 42, 48, 52-54).
- 15 assessed calculated osmolarity using the Khajuria Krahn equation (16, 25, 30, 31, 33, 35, 36, 38, 39, 42, 45, 46, 48, 55, 56), but four did not provide enough information to assess number dehydrated at the 300mMol/L cut-off(30, 39, 45, 55) and four also provided directly measured osmolality which was used in preference to calculated data (31, 36, 42, 48), so data from seven were used in combined analyses (16, 25, 33, 35, 38, 46, 56).
- 11 studies assessed calculated osmolarity using equations other than Khajuria Krahn (17, 19, 21, 26, 27, 29, 32, 34, 44, 47, 49), of which four did not provide data for meta-analysis(19, 26, 27, 29). Five assessed osmolarity or osmolality but the exact method used was not clear and we received no reply from our author enquiries(15, 23, 40, 41, 51). Of these 16 studies (non-Khajuria Krahn equation or unclear) 10 could be combined in meta-analysis(15, 17, 21, 23, 32, 34, 40, 41, 47, 49)
- Two studies assessed fluid intake fairly rigorously(37, 40), but neither provided intake by sex (necessary to assess adequate intake).
- One study assessed salivary osmolality(28).

Study name & type of dehydration assessment	Osmolality (directly measured) in mOsm/kg			Osmolarity (calculated) in mmol/L			Fluid intake assessed? Plus tear osmolarity	Author conclusions on hydration
	Method	Dichotomous data*	Continuous data**	Method	Dichotomous data*	Continuous data**	Y/N	
Aoki 2007<br><br>Osmolality stated, unclear if directly	NA	NA	NA	Method: Blood test<br>Equation used: unclear (called osmolality but no	Cut off: >300 mOsm/kg<br>No (%) above cut off:	Mean (SD): NA<br><br>No (%) >300 mmol/L:	N	Only for broader group (including many younger participants)

measured or calculated				methodology provided)	Meniere's 1/3 (302, 232, 291) >300 other vertigo 0/6 (284, 292, 289, 296, 291, 292)			
Bech 2023<br><br>KK calculated osmolarity	NA	NA	NA	Method: First available blood values for sodium, urea, potassium and glucose (in mmol/l) from emergency ward retrieved from electronic patient record<br><br>Equation used: 1.86 x (Na+ + K+) + 1.15 glucose +urea +14 (all in mmol/L) (KK).	Cut off: 300 mmol/L<br><br>No (%) above cut off: 26 of 114 (22.8%)	Mean (SD): NA median 291.7, IQR 282.6 to 298.9<br><br>No (%) >300 mmol/L:	N (though did ask some questions on fluid intake)	A low correlation was observed between malnutrition and dehydration in older hospitalized patients
Betrosian 1999<br><br>Calculated osmolarity, not KK	NA	NA	NA	Method: Bloods drawn on admission to ICU<br>Equation used: 2Na+ + glucose/18 + urea nitrogen/2.8, all in mg/dl	Cut off: Unclear	Mean (SD):<br>Group 1: 317.3 (16.3)<br>Group 2: 314.3 (25.1)<br><br>No (%) >300 mmol/L: 117 of 157 (75%)	N	Data indicate that hyperosmolality predispose for rhabdomyolysis caused from any type of bacteria.
Bhalla 2000<br><br>Directly measured osmolality <u>and</u>	Method: depression of freezing point (Advanced Micro, Advanced	Cut off:<br>No (%) above cut off:	Mean (SD): 295.2 (9.8)<br><br>No (%) >300mOsm/kg:	Method: Calculated plasma osmolality was also estimated using the equation 2x (sodium +	Cut off:<br><br>No (%) above cut off:<br>Not reported.	Mean (SD):<br><br>No (%) >300 mmol/L: Not reported.	N	The present method of hydration with fluids may be inappropriate

calculated osmolarity, not KK	Instruments), bloods taken within 24 hours of stroke onset		52 of 167 (31%)	potassium) + glucose + urea.				
Bourdel-Marchasson 2004<br><br>Calculated osmolarity, not KK	NA	NA	NA	Method: Osmolarity calculated at admission.<br><br>Equation used: Plasma osmolarity was calculated with the following formula: 2Na+ + 2K+ + BUN + glucose in mmol/L	Cut off: ≥320mmol/L No (%) above cut off: 48 of 436<br><br>No data for a 300 cut-off	Mean (SD): 342.7 (21) BUT osmolarity only reported for the 48 of 436 patients with osmolarity ≥320mmol/L<br><br>No (%) >300 mmol/L: unclear	N	Hyperosmolar states occurred in cognitively impaired and dependent patients
Buaprasert 2021<br><br>Directly measured osmolality	Method: "Fiske Model 210 Micro Osmometer… freezing point depression technique…. analyzed immediately"	Cut off: >300 mOsm/kg<br><br>No (%) above cut off: 80 of 370 (21.6%)	Mean (SD):<br><br>No (%) >300mOsm/kg:	NA	NA	NA	N	One-fifth of older patients admitted to ED presented with" serum osmolality >300 mOsm/kg. Higher levels of comorbidities were a risk factor for low-intake dehydration.
Buoite Stella 2020<br><br>Calculated osmolarity, not KK	NA	NA	NA	Method: Blood samples obtained on admission and analysed in laboratory.<br><br>Equation used: 2Na+ + 2K+ + Glucose + BUN (all values in mmol/L)	Cut off:<br>No (%) above cut off:	Mean (SD): 298 (5)<br><br>No (%) >300 mmol/L: 41 of 119 (34%)	N	Significant association between hypohydration, poor prognosis and worse functional outcome after acute ischemic stroke

Claesson Lingehall 2023<br><br>Directly measured osmolality	Method: author reply states "freezing point depression at -1.858°C method" Baseline measures used.	Cut off: >295mOsm/kg<br><br>No (%) above cut off: 45% of 98 (44) and 40% of 97 (39):<br>total 83 of 195 (43%)<br><br>No data for a 300 cut-off	Mean (SD): 295 (7) mOsm/kg in 195<br><br>No (%) >300mOsm/kg: 46 of 195 (24%)	NA	NA	NA	N	nil (authors were interested in post-intervention changes in delirium)
Duraković 1997<br><br>Osmolality stated, unclear if directly measured or calculated	NA	NA	NA	Method: unclear Equation used: unclear	Cut off: >300 No (%) above cut off: 9/11	Mean (SD): 312 (9)<br><br>No (%) >300 mmol/L: not calculated	N	Unclear - none specific to hydration
El-Sharkawy 2015<br><br>Directly measured osmolality	Method: serum osmolality by freezing point depression from blood sampled at admission	Cut off: >300<br><br>No (%) above cut off: 69 of 187 (37%)	Mean (SD): 288.6 (12.4) mOsmol/kg in 118 euhydrated<br><br>312.4 (16.5) mOsmol/kg in 69 dehydrated	NA	NA	NA	N, no formal assessment of fluid intake, though some estimation	Hyperosmolar dehydration is common in hospitalised older adults and is associated with poor outcome.

El-Sharkawy 2020<br><br>KK calculated osmolarity	NA	NA	NA	Method: data retrieved from the electronic database of a large UK university teaching hospital<br><br>Equation used: 1.86 x (Na+ + K+) + 1.15 glucose +urea +14 (all in mmol/L) (KK).	Cut off: >300 mOsm/l<br><br>No (%) above cut off: 1802 of 6632 (27.2%)	Mean (SD):<br><br>No (%) >300 mmol/L:	N	Hyperosmolar dehydration (HD) is common in hospitalised older adults and is associated with increased LOS, risk of AKI and mortality
Farhan 2019<br><br>Calculated osmolarity, not KK	NA	NA	NA	Method: Blood measurements of serum glucose, Na, and BUN taken from hospital records<br><br>Equation used: 1.86 Na [in mmol/L]) + (glucose [in mg/dL] / 18) + (BUN [in mg/dL] / 2.8) + 9	Cut off:<br>No (%) above cut off:	Mean (SD): only reported as quartiles<br><br>No (%) >300 mmol/L: not calculable	N	Raised serum osmolality is associated with increased risk of AKI and 1-year mortality in patients undergoing PCI.
Fiaux 2015<br><br>Calculated osmolarity, not KK	NA	NA	NA	Method: Day 0 data used.<br><br>Equation used: 2 Na+ + 2 K+ + glucose + urea (all in mM)	Cut off: >295 mOsm/L<br>No (%) above cut off: 69.1% of 97 patients in the DVT + group and 53.2% of 47 patients in the DVT- group<br>92 of 144 were dehydrated	Mean (SD):<br><br>No (%) >300 mmol/L: unclear	N	Dehydration does not appear predictive of the occurrence of DVT

					No data for a 300 cut-off			
Fortes 2015<br><br>Directly measured osmolality	Method: lithium heparin treated blood centrifuged immediately … plasma aspirated and triplicate measurements of osmolality made immediately using a freezing point depression osmometer (Model 330 MO; Advanced Instruments)	Cut off: ≥295 mOsm/kg<br><br>No (%) above cut off: 27 of 130<br><br>No data for a 300 cut-off	Mean (SD):<br>299 (6) for 27<br>283 (6) for 25<br>283 (9) for 78<br>Combined: 286 (10) for 130<br><br>No (%) >300mOsm/kg: 12 of 130 (9%)	NA	NA	NA	N, but saliva ry osmol -ality meas ured	Physical signs and urine markers show little utility to determine if an elderly patient is dehydrated
Gou 2011<br><br>Calculated osmolarity, not KK	NA	NA	NA	Method: Laboratory tests on hospital admission<br><br>Equation used:<br>2x (sodium + potassium) + random blood glucose (all in mmol/L)	Cut off: > 320 mOsm/L<br><br>No (%) above cut off: 7 of 146 (5.1%)<br><br>No data for a 300 cut-off	Mean (SD): NR<br>Median plasma osmolarity was 296.5 (IQR 290.3 to 305.3)<br><br>No (%) >300 mmol/L: unclear	N	Plasma hyperosmolarity may contribute to the death rates of critically ill patients with COVID=19.
Jespersen 2023<br><br>KK calculated osmolarity	NA	NA	NA	Method: Retrospective, serum measures taken within 12 days of nutrition assessment on referral to clinical dietitian.	Cut off: >295mmol/L<br>No (%) above cut off: 40 (40%) of 99<br><br>No data for a 300 cut-off	Mean (SD): Not reported, median 292.6 (IQR 285.6 to 301.0) in 99 patients	N	in older hospitalized patients low-intake dehydration was present in more than 1/3

				Equation used:<br>1.86 x (Na+ + K+) + 1.15 glucose +urea +14 (all in mmol/L) (KK).		No (%) >300 mmol/L: not calculable		
Kafri 2013<br><br>Directly measured osmolality and KK calculated osmolarity	Method: Serum osmolality analysed using freezing point depression (Advanced Instruments model 2020 osmometer), on admission	Cut off: >300<br><br>No (%) above cut off: 6 of 27 (22%)	Mean (SD): 295.5 (7.5)<br><br>No (%) >300mOsm/kg: not calculated	Method: Abbott c8000 analyser (Abbott Laboratories) standardised and automated<br><br>Equation used: serum osmolarity ([2Na+]+[2K+]+Urea+ Glucose, all in mmol/L) (KK)	Cut off: >300<br><br>No (%) above cut off: 12 of 27 (44%)	Mean (SD): 298.2 (6.9)<br><br>No (%) >300 mmol/L: 12 of 27	N	MF-BIA is not appropriate for diagnosing dehydration post stroke as it has low diagnostic accuracy.
Khanimov 2020<br><br>Calculated osmolarity, equation not reported	NA	NA	NA	Method: osmolarity calculated at admission<br>Equation used: not reported	Cut off:<br>No (%) above cut off:	Mean (SD): Reported as 3 groups.<br>Overall: mean 303.6 (14.8) in 7718 patients<br><br>No (%) >300 mmol/L: 4670 of 7718 (61%)	N	Unclear - none specific to hydration
Lauriola 2018<br><br>KK calculated osmolarity	NA	NA	NA	Method: not reported<br><br>Equation used:<br>1.86 x (Na+ + K+) + 1.15 glucose +urea +14 (all in mmol/L)	Cut off: 295 and >300<br><br>No (%) above cut off: 574 of 845 reported as "dehydrated"	Mean (SD): Overall: 297.5 (6.3) in 1091<br><br>No (%) >300 mmol/L: 292 of 845 (43%)	N	Those with cognitive impairment had increased risk of dehydration ($p \leq 0.001$). In multivariate analysis dehydration was associated with ADL ($p < 0.001$) and IADL ($p <$

				(KK).	but unclear if relates to 295 or >300 cut off (so not used)			0.001), but not age, pressure sore risk of nutritional status.
McCrow 2016<br><br>Calculated osmolarity, equation not reported	NA	NA	NA	Method: unclear, collected at baseline Equation used: not reported	Cut off: >300<br><br>No (%) above cut off: 3 of 44 (7%)	Mean (SD): not reported<br><br>No (%) >300 mmol/L:	N	dehydration is common among older hospitalized patients and frailty may increase the risk for dehydration in cognitively intact older adults
Miller 2019<br><br>KK calculated osmolarity	NA	NA	NA	Method: Assessed on admission<br><br>Equation used: 1.86 x (Na+ + K+) + 1.15 glucose +urea +14 (all in mmol/L) (KK).	Cut off: >300<br><br>No (%) above cut off:<br>9 of 41 (22%)	Mean (SD): not reported<br><br>No (%) >300 mmol/L:	N	Dehydration is common in stroke patients admitted to hospital but assessment and diagnosis of dehydration is not routinely documented
Munk 2021<br><br>Directly measured osmolality <u>and</u> KK calculated osmolarity	Method: Non-fasting venous routine standard blood sample (before any iv fluids administered) with laboratory tests for Na+, K+, urea, glucose and also directly measured osmolality.	Cut off: >300<br><br>No (%) above cut off:<br>10 of 90 (11%)	Mean (SD): not stated, median 291 (IQR 281-297) for all 90 participants<br><br>No (%) >300mOsm/kg:	Method: as before<br><br>Equation used: 1.86 x (Na+ + K+) + 1.15 glucose +urea +14 (all in mmol/L) (KK).	Cut off:<br><br>No (%) above cut off:	Mean (SD): Not reported, median 293 (IQR 287-299)<br><br>No (%) >300 mmol/L: not calculable<br><br>11 of 90 taken from Fig 2	N	The [KK] equation … was found to be an accurate objective diagnostic tool to assess low-intake dehydration in older hospitalised medical patients and markedly superior to current clinical practice.
Murray 2016<br><br>Fluid intake	NA	NA	NA	NA	NA	NA	Y (for detail	those in the water protocol group consumed on average

							s see separ ate table)	38% and those in the thickened liquids only group consumed 53% of their calculated daily fluid requirements
Nagae 2023<br><br>KK calculated osmolarity	NA	NA	NA	Method:<br>First blood tests (within 48 hours of admission)<br><br>Equation used:<br>1.86 x (Na+ + K+) + 1.15 glucose +urea +14 (all in mmol/L) (KK).	Cut off:<br>>300<br><br>No (%) above cut off:<br>60 of 192 (31%)	Mean (SD):<br>297.9 (11.4)<br><br>No (%) >300 mmol/L:	N	Dehydration predicted hospital associated disability in acute hospitalized older adults.
Nielsen 2024<br><br>KK calculated osmolarity	NA	NA	NA	Method: Plasma isolated from whole blood, collected on test days.<br><br>Equation used:<br>1.86 x (Na+ + K+) + 1.15 glucose +urea +14 (all in mmol/L) (KK).	Cut off:<br>>295mmol/L<br><br>No (%) above cut off:<br>12 of 38 or 32%<br><br>>300 cut off not reported	Mean (SD): not reported<br><br>No (%) >300 mmol/L:	N	32% of our study sample was dehydrated.
Oh 2007<br><br>Fluid intake and osmolality	NA	NA	NA	Method: serum electrolyte levels on the day before tube feeding collected by	Cut off:<br><br>No (%) above cut off:	Mean (SD):<br>306.1 (41)	Y (for detail s see	fluid imbalance and over-hydration incidences were significantly

stated, unclear if directly measured or calculated				retrospective medical record review<br><br>Equation used: Not stated		No (%) >300 mmol/L: 48 of 85 (56%)	separ ate table)	increased after tube feeding
Palmisano 2014<br><br>Osmolality stated, unclear if directly measured or calculated	NA	NA	NA	Method:<br>Equation used: unclear	Cut off:<br>No (%) above cut off:	Mean (SD): 294.1 (15.6)<br><br>No (%) >300 mmol/L: 28 of 79 (35%)	N	Compared with other patients, those observed during the hottest months were significantly older and more likely to have higher osmolality (p=0.049)
Pfortmueller 2014<br><br>Directly measured osmolality and KK calculated osmolarity	Method: freezing point depression (Advanced 3900 osmometer, CV <1%)	Cut off: >300 mOsm/kg<br><br>No (%) above cut off:<br>6 of 54 (11%)	Mean (SD): 290.9 (8.6)<br><br>No (%) >300mOsm/kg:	Method:<br>Equation used: 1.86 x (Na+ + K+) + 1.15 glucose + urea + 14 (all in mmol/L) (KK)	Cut off:<br>No (%) above cut off:	Mean (SD): 291.7 (10.8)<br><br>No (%) >300 mmol/L: 12 of 54 (22%)	N	None relating to dehydration
Pliquett 2020<br><br>Calculated osmolarity, not KK	NA	NA	NA	Method: not stated<br>Equation used: 2x Na + Urea + Glucose (all in mmol/L).	Cut off:<br><br>No (%) above cut off:	Mean (SD): In 93 patients without hyponatremia: 308.4 (18.9)<br><br>No (%) >300 mmol/L: unclear for full group	N	All CRS patients showed a high prevalence of diabetes mellitus and a high one-year mortality.
Sabanovic 2022	NA	NA	NA	Method: potassium, sodium, urea,	Cut off: >295 mmol/L	Mean (SD): not reported	N	Pre-operative osmolarity is associated with severe

KK calculated osmolarity				glucose routinely measured within 24 hours of admission to ED, retrieved post hoc from electronic patient records<br><br>Equation used:<br>1.86 x (Na+ + K+) + 1.15 glucose + urea + 14 (all in mmol/L) (KK)	No (%) above cut off:<br>86 of 214 (40%) pre-operatively<br><br>>300 not reported	No (%) >300 mmol/L:		frailty at discharge and prolonged hospital stay in older patients with hip fractures.
Sanson 2021<br><br>KK calculated osmolarity	NA	NA	NA	Method:<br><br>Equation used:<br>1.86 x (Na+ + K+) + 1.15 glucose + urea + 14 (all in mmol/L) (KK)	Cut off: >300<br><br>No (%) above cut off: 2385 of 4613 (52%) participants	Mean (SD): Not reported, median 301 (IQR 293.7 to 310.6)<br><br>No (%) >300 mmol/L:	N	impaired hydration is common in older people acutely admitted to medical care and is associated with poor outcome.
Shen 2017<br><br>Calculated osmolarity, not KK	NA	NA	NA	Method: initial osmolarity after intensive care admission used. Only values measured at the same time were used in calculations.<br><br>Equation used:<br>2 Na+ + 2 K+ + (glucose/18) + (urea/2.8). [units not specified]	Cut off:<br>No (%) above cut off:	Mean (SD): 302.8 (11.8)<br><br>No (%) >300 mmol/L: 9937 of 16598 (60%)	N	hyperosmolarity is associated with increased mortality in patients who are critically ill with cardiac, cerebral, vascular and gastrointestinal admission diagnoses, but not for respiratory disease
Sjöstrand 2013	Method:	Cut off:	Mean (SD): 299.7 (7)	Method: sodium, potassium, urea,	Cut off:	Mean (SD): 301.3 (5.8)	N	None relevant to dehydration

Directly measured osmolality and KK calculated osmolarity	Venous serum samples analysed immediately at ISO-certified laboratory using freezing point depression (Osmometer Advanced 2020, CV unclear)	>300 mOsm/kg<br><br>No (%) above cut off:<br>16 of 36 (44%)	No (%) >300mOsm/kg:	glucose by indirect ISE, glucose assessment was duplicated and mean recorded, Hitachi 917<br><br>Equation used:<br>1.86 x (Na+ + K+) + 1.15 glucose + urea + 14 (all in mmol/L) (KK)	No (%) above cut off:	No (%) >300 mmol/L: 21 of 36 (59%)		
Sokolski 2019<br><br>Calculated osmolarity, not KK	NA	NA	NA	Method: Admission serum osmolarity was calculated<br><br>Equation used:<br>1.86 x sodium [mmol/L] + (glucose [mg/dL]/18) + (urea [mg/dL]/2.8) + 9)	Cut off: quartiles of osmolarity reported<br><br>No (%) above cut off:	Mean (SD): 296 (14) for 361<br><br>No (%) >300 mmol/L: 131 of 338 (39%)	N	serum osmolarity associated with clinical status and both in-hospital and out-of-hospital outcomes.
Tellini 2005<br><br>Osmolality stated, unclear if directly measured or calculated	NA	NA	NA	Method:<br>appears only to be reported in those with hyponatraemia<br><br>Equation used:<br>not reported	Cut off:<br>No (%) above cut off:	Mean (SD): 265.3 (9.6) in 15 w hyponatraemia, not reported in the 88 without hyponatraemia<br><br>No (%) >300 mmol/L: not calculable	N	In those with hyponatremia, plasma osmolarity was always below the lower limit of the normal range
Van Wijk 2023	Method: freezing point depression	Cut off:	Mean (SD): 294.5 (6.3) for 34 stroke	NA	NA	NA	N<br>Water intake	Stroke patients had poorer hydration status (higher blood osmolality)

Directly measured osmolality	assessed within 10 days of recruitment (post stroke)	No (%) above cut off:	patients with dysphagia (patients without dysphagia omitted)<br><br>No (%) >300mOsm/kg: 7 of 34 (19%)				assessed as part of a 24-hour food diary only	compared to, and reported water intake of only 80% of, healthy controls.
Vivanti 2008<br><br>Change data<br><br>Directly measured osmolality	Method:<br>Author reply 4Oct2024 states freezing point depression. Paper states non-fasting blood sample analysed in the hospital laboratory under standard conditions.	Cut off: >295<br><br>No (%) above cut off: 7 (17%) of 43<br><br>No data for a 300 cut-off	Mean (SD): Combined groups 275.4 (10.2) n=43<br><br>No (%) >300mOsm/kg: 0 of 43 (0%)	NA	NA	NA	N<br>But asked about fluid intake over 3 years	Physical, rather than biochemical, parameters more often identified mild dehydration.
Walsh 2012<br><br>Directly measured osmolality <u>and</u> tear osmolality	Method:<br>Blood collected into vacutainer tube containing lithium heparin (Becton Dickinson, Oxford, UK), spun, freezing point depression assessed in triplicate (Model 330 MO;	Cut off:<br><br>No (%) above cut off:	Mean (SD):<br>DE group 288 (11) n=54<br>CON group 284 (10) n=45<br><br>Whole group 286.2 (10.7) n=99<br><br>No (%) >300mOsm/kg: 10 of 99 (10%)	NA	NA	NA	N<br><br>But tear osmolality assessed	Those with dry eyes have higher osmolality than those without

	Advanced Instruments osmometer, Norwood, MA)							
Wojszel 2020<br><br>KK calculated osmolarity	NA	NA	NA	Method: assessed on admission<br><br>Equation used: 1.86 x (Na+ + K+) + 1.15 glucose + urea + 14 (all in mmol/L) (KK)	Cut off: >295 mMol/L<br><br>No (%) above cut off: 209 of 358 (58.4%)<br><br>>300 not reported	Mean (SD): not stated, Median 296.5 (IQR 292.2 to 299.6)<br><br>No (%) >300 mmol/L: unable to calculate	N	Chronic kidney disease, diabetes, taking procognitive medications and hypertension were the main variables predicting raised osmolarity in multivariate analysis
Zanetti 2022<br><br>KK calculated osmolarity	NA	NA	NA	Method: admission laboratory data were collected from the hospital electronic archive and medical records<br><br>Equation used: 1.86 x (Na+ + K+) + 1.15 glucose + urea + 14 (all in mmol/L) (KK)	Cut off: > 300 mmol/L<br><br>No (%) above cut off: 151 of 529 (28.5%)	Mean (SD): 295.1 (13.6)<br><br>No (%) >300 mmol/L: not calculated	N	Impaired hydration is a potentially modifiable risk factor for severe outcomes in older acutely hospitalized patients.

*Dichotomous data were numbers dehydrated given within the publication

**Continuous data were used where possible to estimate numbers above the 300 cut-off assuming normal distribution of osmolal data.

KK: osmolarity calculated using the validated Khajuria Krahn equation(57)

Supplementary File 6. Table of fluid intake data

Study name	Method of assessment	Fluid intake	Conclusions
Murray 2016<br><br>Fluid intake	Intake calculated from fluid balance charts (drinks only) recorded by nursing staff over 24 hours. Soups, custards, ice-cream, yoghurt not included. Fluid balance charts completed daily.<br><br>Compared fluid intake in those on thickened fluid only vs thickened fluid plus free water. Intake goal 30 ml per kg body weight and separately BUN/Cr <20.	Mean (SD):<br>not reported (only BUN/Cr)<br><br>No (%) with >1.6 L in women, >2.0L in men: not reported<br><br>71 % of participants were classified as dehydrated at entry to the study with BUN/Cr ratio >20	Those in the water protocol group consumed on average 38% and those in the thickened liquids only group consumed 53% of their calculated daily fluid requirements
Oh 2007<br><br>Fluid intake and osmolality stated, unclear if directly measured or calculated	Fluid balance (intake/output) on four consecutive days (from the day before to third day of tube feeding) collected by retrospective medical record review.	24-hour intake before tube feeding: 3145ml/d (SD 2281)<br>24-hour input-output before: 121ml/d (SD 716)	Fluid imbalance and over-hydration incidences were significantly increased after tube feeding

Supplementary File 7. Description of risk of bias of all 40 included studies

Summary: Twenty three of the 40 included studies (58%) clearly assessed dehydration in a reliable way, using directly measured osmolality or calculated osmolarity using the Khajuria Krahn equation. No studies clearly assessed fluid intake reliably. Recruitment was appropriate in 21 studies (53%), unclear in three and at high risk of bias in the remaining 16 studies. Participants and setting were well described in 20 studies (50%), with significant gaps in the remaining 20. The sampling frame focussed on older adults in 27 included studies (68%), was unclear in two and more mixed in the remaining 11. Data analysis had sufficient coverage of the sample in 28 studies (70%), was unclear in two and problematic in the remaining 10. Studies directly reported the number of dehydrated participants (using our cut offs) and the sample size in 15 studies (38%), we had to estimate dehydration from mean osmolality or osmolarity or were not able to estimate dehydration numbers in the remaining 25 studies. The response rate was judged adequate in 20 studies (50%), unclear in nine and inadequate in 11. No further issues were identified in 33 studies (83%), but further issues raised in seven.

Study ID	Dehydration measure reliable	Fluid measure reliable	Recruitment appropriate	Participants & setting described	Sample frame appropriate to recruit older adults	Data analysis with sufficient coverage of sample	No participants dehydrated and total sample size reported	Response rate adequate	Further issues
Aoki 2007		N/A							
Bech 2023		N/A							
Betrosian 1999		N/A							
Bhalla 2000		N/A							
Bourdel-Marchasson 2004		N/A							
Buaprasert 2021		N/A							
BuoiteStella 2020		N/A							
ClaessonLingehall 2023		N/A							
Durakovic 1997		N/A							
El-Sharkawy 2015		N/A							
El-Sharkawy 2020		N/A							

Farhan 2019		N/A							
Fiaux 2015		N/A							
Fortes 2015		N/A							
Gou 2021		N/A							
Jespersen 2023		N/A							
Kafri 2013		N/A							
Khanimov 2020		N/A							
Lauriola 2018		N/A							
McCrow 2016		N/A							
Miller 2019		N/A							
Munk 2021		N/A							
Murray 2016	N/A								
Nagae 2023	☺	N/A							
Nielsen 2024		N/A							
Oh 2007									
Palmisano 2014		N/A							
Pfortmueller 2014		N/A							
Pliquett 2020		N/A							
Sabanovic 2022		N/A							
Sanson 2021		N/A							
Shen 2017		N/A							
Sjostrand 2013		N/A							
Sokolski 2019		N/A							
Tellini 2005		N/A							
vanWijk 2023		N/A							
Vivanti 2008		N/A							
Walsh 2012		N/A							

Wojszel 2020		N/A							
Zanetti 2022		N/A							

Green: low risk of bias

Orange: unclear risk of bias

Red: high risk of bias

N/A: not applicable

Supplementary File 8. Sensitivity analyses.

Sensitivity analyses for the prevalence of low-intake dehydration in older hospitalised adults by dehydration assessment method using combined data on directly measured and calculated with KK osmolality/ osmolarity

Characteristic	Mean (95% CI) pooled prevalence, I^2 %	Level of asymmetry by Doi plot	I^2 (measure of heterogeneity between studies)
Main analysis: RE MA, double arcsine	23% (17 to 30%)	major asymmetry	98%
RE MA, logit	24% (18 to 30%)	major asymmetry	98%
QE MA, double arcsine	34% (19 to 50%)	major asymmetry	98%
QE MA, logit	34% (19 to 50%)	major asymmetry	98%
IVhet MA, double arcsine	35% (18 to 52%)	major asymmetry	98%
IVhet MA, logit	35% (18 to 52%)	major asymmetry	98%
Removing outliers (RE MA DA)	Sanson 2021 removed: 22% (19 to 26%)	minor asymmetry	89%

Supplementary File 9. Detailed account of SWiM analysis and subgrouping.

We aimed to use subgrouping to understand which groups of older adults were more likely to be dehydrated. Information on the characteristics of included studies related to subgrouping by potential causal factors are in Appendix 4. The associations (noted below) were generally univariate analyses, and confounders not adjusted for. We defined a positive association to be when being healthier was associated with lower levels of low-intake dehydration (or worse health/wellbeing associated with higher risk of dehydration).

Dependency

Meta-analytic subgrouping by dependency and functional status was not feasible as functional status was not mentioned in eight of the 19 included studies, and was measured in a variety of ways in the remainder (including Barthel index, activities of daily living (ADL), instrumental activities of daily living (IADL), Rankin score, NIHSS score, the Norton scale, presence of not of dysphagia and whether participants were able to go out or get out of a bed or chair. Data were provided as mean, median or number below/above specific cut-offs, and for the whole scale or sub-scores. Additionally, functional status although reported, was not reported by hydration status in most studies(22, 25, 35, 36, 42, 46, 48, 54, 56). As meta-analytic subgrouping was not possible, we carried out SWiM analysis using vote-counting of studies assessing the relationship between hydration and some measure of dependency. This method was also used for other subgrouping components.

Three studies found that higher dependency was associated with statistically significantly higher risk of dehydration (positive association):

1. Bhalla(18) found that mean admission osmolality was significantly higher in patients dependent after their stroke,
2. Lauriola(33) found that dehydration was associated with poorer ADL scores (mean 5.2 hydrated, 4.8 dehydrated, p<0.001) and IADL scores (mean 6.1 hydrated, 5.0 dehydrated, p<0.001)
3. Nagae(38) found that dehydrated participants had significantly poorer Barthel Index scores (median 80, IQR 50-95) than hydrated participants (median 95, IQR 75-100, p<0.01), and poorer IADL scores (median 3, IQR 0-7 vs 6, 2-7, p=0.014) at baseline.

No studies found that higher dependency was associated with statistically significantly lower risk of dehydration (negative association).

Five found no statistically significant association between dependency and dehydration:

1. Bech(16) suggested that there was no statistically significant association between dependency and hydration status (unclear association)
2. Buaprasert(20) found complete dependence (by ADL, higher ADL suggests greater disability) in 5% of those who were dehydrated, and 9% of those not dehydrated (p=0.25, negative association)
3. El-Sharkawy 2015(24) found that mean Barthel Index (higher scores means greater independence) was similar in 69 dehydrated participants (14.7, SD 5.7) and 118 euhydrated (15.2, SD 5.2, p=0.63, positive association).
4. Kafri(31) found pre-morbid Rankin scores indicating moderate to severe disability (3-5) in 2 of 6 (33%) dehydrated participants and 2 of 21 euhydrated (10%, positive association)

5. Van Wijk(52) found osmolarity was not significantly different between stroke patients with dysphagia (294.5mOsm/kg, SD 6.3, n=x) or without dysphagia (295.2mOsm/kg, SD 6.9, n=z, negative association).

Cognition
Of the 19 studies,13 did not assess cognition or did not report associations with hydration. In the remaining six studies cognition was assessed in various ways, including the Mini-mental state exam (MMSE), six-item cognitive impairment scale, dementia diagnosis or specific types of dementia diagnosis. Vivanti(53) found that no participants were diagnosed with dementia so could not assess the relationship with hydration.

Three studies found that poorer dehydration was associated with poor cognition (positive association):
1. El-Sharkawy 2020(25) reported dementia in 6.4% of participants with dehydration, and 2.0% of those who were euhydrated, p<0.001.
2. Lauriola(33) found mean osmolarity of 298.4mmol/L (SD 5.5) in 571 people with cognitive impairment, and of 296.53mmol/L (SD 6.98) in 520 without impairment (OR 2.02, 95% CI 1.47 to 2.76 in those with poorer cognition, p<0.001).
3. Nagae(38) found that dehydrated participants had significantly lower MMSE (greater cognitive impairment, median 20, IQR 8-25) than well hydrated (median 23, IQR 18-27, p<0.01).

No studies found that poorer cognition was statistically significantly associated with better hydration (negative association). Two studies found no statistically significant association between cognition and dehydration:
1. Buaprasert(20) found no relationship between cognitive status and hydration using either the 6-item cognitive impairment test (higher scores indicated greater impairment, median 5, IQR 4-6.7 in dehydrated, median 7, IQR 6-8 in hydrated participants, p=0.334, negative association) or dementia diagnosis (3.8% of the dehydrated participants and 6.9% of dehydrated participants were diagnosed with dementia, p=0.434, positive association).
2. El-Sharkawy 2015(24) found mean MMSE of 24.5 (of 30, SD 5.9) in 118 euhydrated participants, compared to 24.4 (SD 6.6, p=0.93) in 69 dehydrated participants (positive association).

Diabetic status
Twelve studies did not assess diabetic status or did not assess the relationship between diabetes and hydration. Of the seven which did assess this relationship, three found that being diabetic increased the risk of dehydration or higher osmolality (positive association):
1. Bech(16) found higher prevalence of diabetes among those who were dehydrated (28.6% vs 12.3%, p=0.034)
2. Bhalla(18) found that diabetic patients had higher osmolality (mean 300mOsm/kg) than non-diabetics (mean 294, 95% CI of the difference -11 to -1.4, p=0.01).
3. Vivanti(53) found having diabetes was associated with increased odds of dehydration (OR 1.1 to 2)

No studies found that those with diabetes were statistically significantly less likely to be dehydrated (negative association). Four studies found no statistically significant association between diabetes and hydration:

1. Buaprasert(20) found diabetes with end organ damage in 26.3% of dehydrated and 20.3% of hydrated participants, p=0.257 in univariate analysis (positive association)
2. Kafri(31) found mean serum glucose 8.4mmol/L (SD 4.3) in 12 hydrated participants, mean 6.3mmol/L (SD 1.8) in 9 with impending dehydration, and 8.8 (SD 3.9) in those with dehydration (>300mOsm/kg, unclear association)
3. Munk(36) found no relationship between dehydration and diabetic diagnosis (unclear association).
4. Nagae(38) found that dehydrated participants had slightly higher risk of diabetes (36.7%) as well hydrated participants (25.8%, p=0.12, positive association)

Renal impairment
Twelve studies did not assess renal status, excluded those with renal disease or did not report associations between dehydration and renal health. Of the remaining seven, six studies found that those with poorer renal function were more likely to be dehydrated (positive association):

1. Bech(16) found a higher prevalence of those with poor renal function (eGRF <30) among those who were dehydrated (22% vs 5%, p=0.008)
2. Buaprasert(20) found moderate or severe renal disease in 41.3% of dehydrated participants and 23.1% of hydrated (p=0.001, and a similar relationship with eGFR) in univariate analysis.
3. El-Sharkawy 2015(24) found eGFR at admission was higher (mean 62.8, SD 18.8) in the 118 euhydrated participants, compared to in the 69 dehydrated participants (mean 46.1, SD 19.5, p<0.001). They also found that a clinical diagnosis of acute kidney injury was more common in those who were dehydrated (p<0.001).
4. Nagae(38) found that dehydrated participants had poorer renal function (eGFR mean 46.2, SD 24.2) than well hydrated participants (59.9, SD 19.6).
5. Sanson(46) found a significant correlation between osmolarity and eGFR, r=-498
6. Vivanti(53) found renal calculi were associated with increased risk of dehydration (OR 2.8, 95%CI 0.2 to 36.4)

No studies found that poorer renal function was associated with better hydration (negative association). Munk(36) excluded those with renal failure or eGFR<30, and found that in the remaining participants there was no significant relationship between dehydration and eGFR (unclear association).

Economic status
All the included studies were from high-income countries except for Buaprasert (Thailand, an upper-middle income economy(20)), so subgrouping by economic status was not possible.

Age
Nine of the 19 studies did not report assessing a relationship between age and hydration status. Among the ten that did, one study found that increasing age was associated with statistically significantly increased osmolality or risk of dehydration: Bhalla(18) found that age was significantly associated with plasma osmolality (coefficient = 0.15 per year increase in age, 95% CI 0.03 to 0.27; P=0.02, positive association).

None found that increasing age was associated with statistically significant lower osmolality or lower risk of dehydration, and eight found no statistically significant relationship between age and hydration, of which five suggested positive associations:

1. El-Sharkawy 2020(25) found the mean age of participants with dehydration was 79 years (SD 8.3) and without dehydration 77 years (8.1), not statistically significantly different but a positive association
2. Buaprasert(20) found that the odds of dehydration not statistically significantly higher in those aged at least 80 years (OR 1.35, 95% CI 0.82 to 2.22) in univariate analysis (positive association)
3. Vivanti(53) found a similar age in dehydrated (mean 78.5 years, SD 8.6, n=7) and well hydrated (77.1, 6.8, n=36) participants (positive association)
4. Fortes(28) reported the mean (SD) age of those with low-intake dehydration as 78.3 (9.6), while that of the euhydrated participants was 76.3 (7.7) (p>0.05, positive association).
5. Nagae(38) found that age was not associated with dehydration - mean age 84.3 years (SD 5.7) in hydrated participants, 85.5 (5.8) dehydrated, p=0.21 (positive association)

Four suggested unclear associations:

1. Lauriola(33) suggested that dehydration risk was not associated with age (r2=0.0046, p=0.77, unclear association)
2. Kafri(31) found a mean age of 72.3 years (SD 12.5, n=12) in hydrated participants, 73.5 (11.4, n=9) in dehydrated, 68.7 years (SD 8.0, n=6) impending participants (unclear association)
3. Munk(36) reported no association between participant age and hydration status (p=0.40, unclear association)
4. Bech(16) reported that there were “no other differences in baseline characteristics”, so presumably found no statistically significant relationship between age and hydration status, unclear association

One suggested a negative association:

1. El-Sharkawy 2015(24) found that the mean age was 82 years (SD 7) in euhydrated participants, 81 years (SD 8) in dehydrated participants, p=0.86 (negative association)

Subgrouping by mean or median age of participants was possible for 17 of the 19 studies (2 did not report participant age(25, 46)) but there was no suggestion of a linear association between age and dehydration prevalence (Appendix 8).

Gender
Twelve studies did not report whether they assessed any relationship between gender and hydration. Of the remaining seven, no studies found that women were more at risk of dehydration, but three found that men were more at risk of dehydration:

- Bech(16) found that fewer females (42.9% vs. 67.7%, p = 0.013) were dehydrated
- El-Sharkawy 2020(25) found that 25% of women were dehydrated, and 29% of men, p<0.001
- Munk(36) found that dehydration was more common in men than women (p<0.05)

Four found no relationship between gender and dehydration:

- Buaprasert(20) found no relationship, p=0.915 in univariate analysis
- El-Sharkawy 2015(24) reported that 34% of men were dehydrated, and 40% of women, p=0.38

- Nagae(38) found that gender was not associated with dehydration - 42.4% male in the hydrated group, 38.3% male in dehydrated, p=0.59
- Vivanti(53) found that 18% of women and 13% of men were dehydrated (p-value not stated, but numbers of participants were small)

Ethnicity
Most studies did not report ethnicity of participants. All participants in Buaprasert(20) were of Thai origin, all participants in Lauriola and Nielsen(33, 39) were Caucasian, as were almost all van Wijk(52) participants (one was Asian). No studies reported dehydration status by ethnicity.

Comorbidities
Sixteen studies did not report the relationship between number of comorbidities and dehydration, while two studies found a statistically significant positive association, and one a non-statistically significant, unclear relationship.

- Buaprasert(20) found that Charlston Comorbidity Index (CCI, a count of comorbidities) score ≥5 was associated with serum osmolarity >300 mOsm/kg among older patients (adjusted odds ratio: 1.82; 95% confidence interval: 1.03–3.21, p=0.038 in multivariate analysis, positive association). Specific comorbidities associated with raised serum osmolality in univariate analysis for conditions with at least 10 participants included myocardial infarction (p=0.002), congestive heart failure (p=0.008), peripheral vascular disease (0.038), moderate or severe renal disease (p=0.001), but not with cerebrovascular disease, dementia, chronic pulmonary disease, ulcer, hemiplegia, diabetes with end organ damage, or any tumour.
- Nagae(38) found that those who were dehydrated had more comorbidities (median 3, IQR 1-4) than those who were well hydrated (median 2, IQR 1-3, p=0.02, positive association).
- El-Sharkawy 2015(24) found no relationship between dehydration at admission and CCI, median CCI score was 4 for both dehydrated and euhydrated participants (p=0.22).

Settings
We were able to subgroup studies by setting in meta-analysis, see Appendix 8. There were no clear differences in dehydration prevalence between older adults in geriatric wards, emergency or acute care, post-stroke or planned admission settings.

Supplementary File 10. Subgrouping by participant age in years

Random effects meta-analysis, using double arcsine transformed data, including the 17 studies with directly measured osmolality or calculated osmolarity using the Khajuria Krahn equation that also reported a mean or median age for participants (not reported by 2 studies (25, 46). Subgrouping is by mean (or median) age in years.

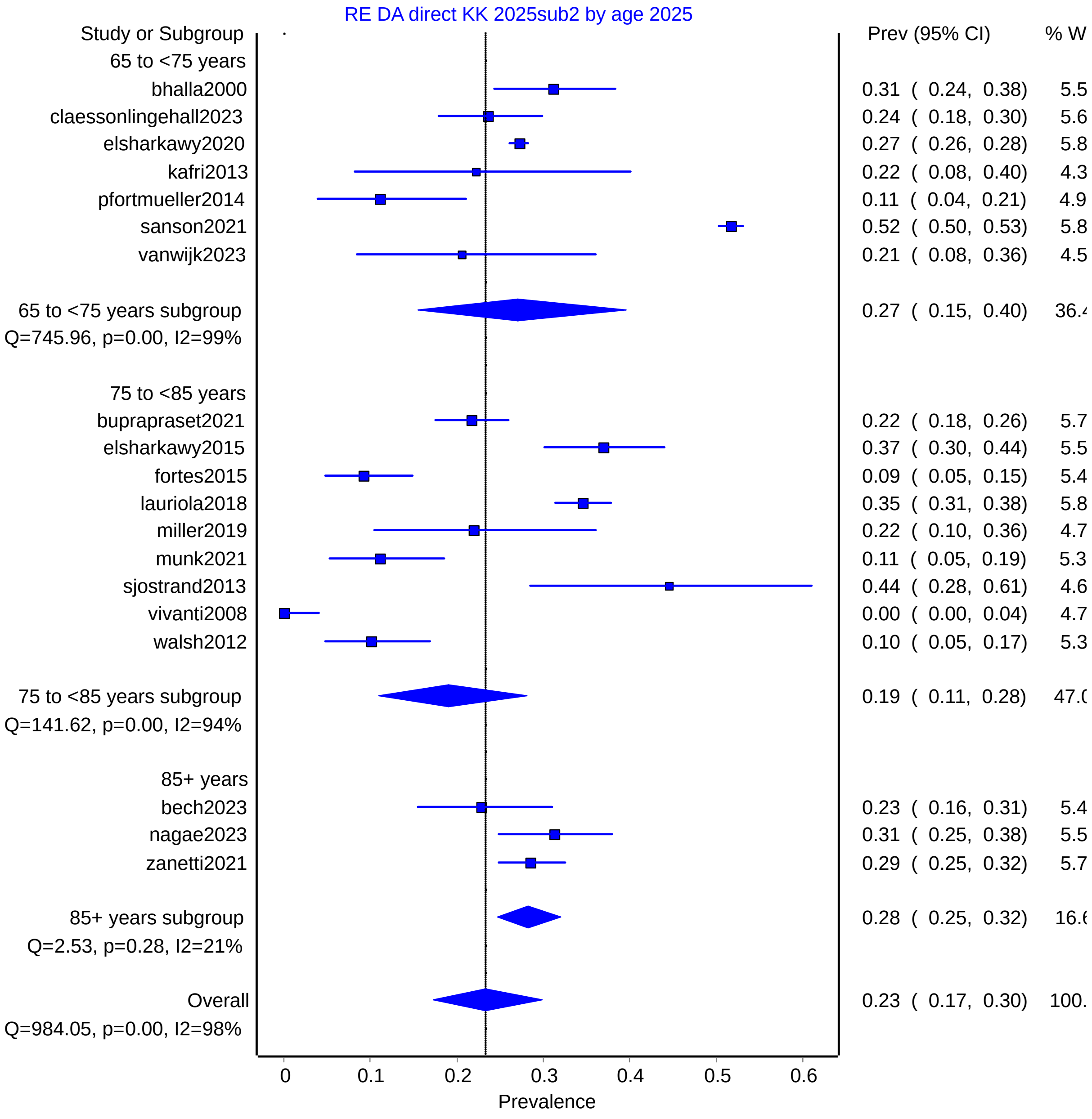

Supplementary File 11. Subgrouping by setting

Random effects meta-analysis, using double arcsine transformed data, including the 19 studies with directly measured osmolality or calculated osmolarity using the Khajuria Krahn equation. Subgrouping is by type of hospital setting (geriatric unit, ED or acute care, and post-stroke).

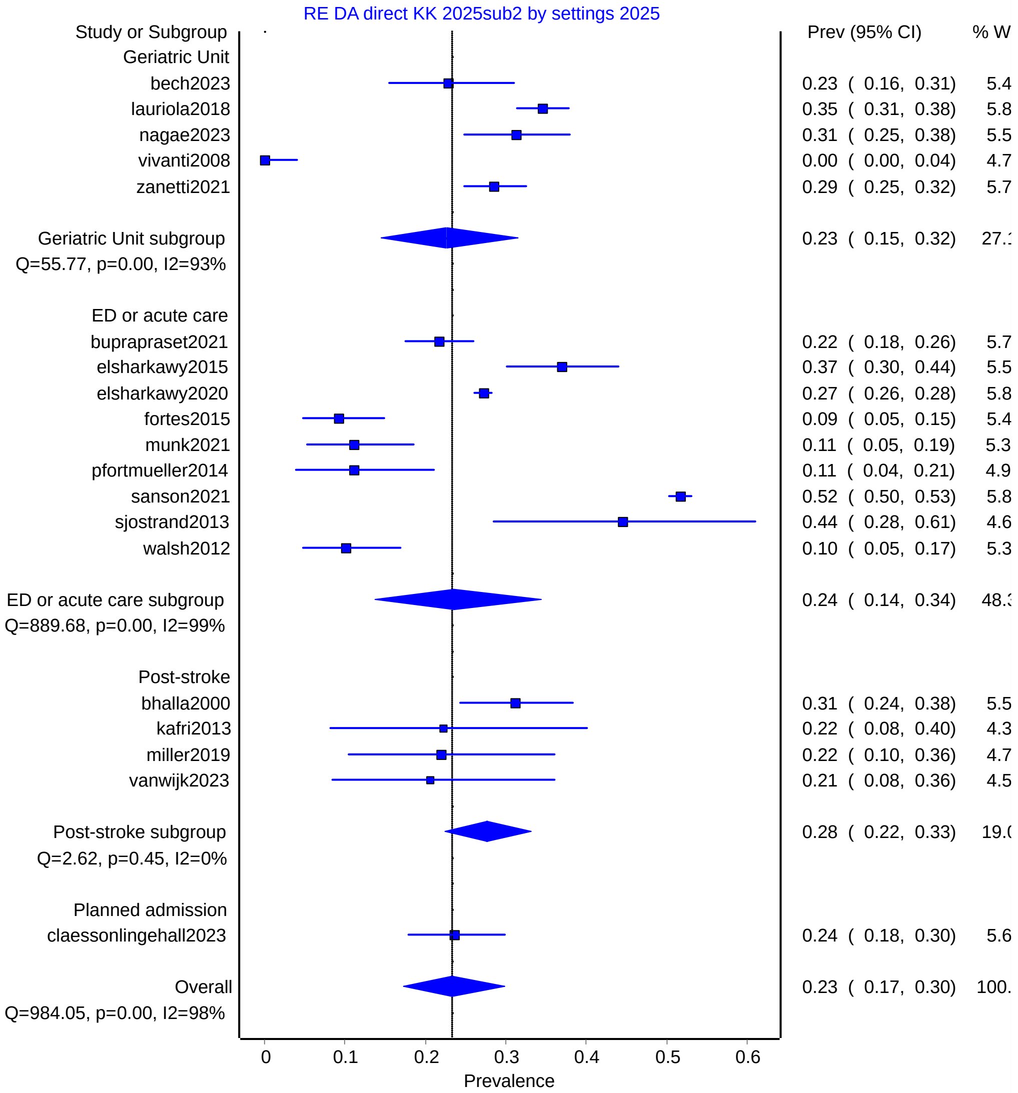

References

1. Barendregt JJ, Doi SA, Lee YY, Norman RE, Vos T. Meta-analysis of prevalence. Journal of Epidemiology and Community Health. 2013;67(11):974-8.
2. Röver C, Friede T. Double arcsine transform not appropriate for meta-analysis. Research Synthesis Methods. 2022;13(5):645-8.
3. Saha S, Chant D, McGrath J. Meta-analyses of the incidence and prevalence of schizophrenia: conceptual and methodological issues. Int J Methods Psychiatr Res. 2008;17(1):55-61.
4. Borges Migliavaca C, Stein C, Colpani V, Barker TH, Munn Z, Falavigna M, on behalf of the Prevalence Estimates Reviews – Systematic Review Methodology G. How are systematic reviews of prevalence conducted? A methodological study. BMC Medical Research Methodology. 2020;20(1):96.
5. Barker TH, Migliavaca CB, Stein C, Colpani V, Falavigna M, Aromataris E, Munn Z. Conducting proportional meta-analysis in different types of systematic reviews: a guide for synthesisers of evidence. BMC Medical Research Methodology. 2021;21(1):189.
6. 5.3 Systematic reviews of prevalence and incidence. 2020. In: JBI Manual for Evidence Synthesis [Internet]. Available from https://jbi-global-wiki.refined.site/space/MANUAL/355863557/Previous+versions?attachment=%2Fdownload%2Fattachments%2F355599504%2FJBIMES_2021April.pdf&type=application%2Fpdf&filename=JBIMES_2021April.pdf: JBI.
7. Stanley TD, Doucouliagos H. Neither fixed nor random: weighted least squares meta-analysis. Statistics in Medicine. 2015;34(13):2116-27.
8. Doi SA, Barendregt JJ, Khan S, Thalib L, Williams GM. Advances in the meta-analysis of heterogeneous clinical trials I: The inverse variance heterogeneity model. Contemp Clin Trials. 2015;45(Pt A):130-8.
9. Doi SA, Barendregt JJ, Khan S, Thalib L, Williams GM. Advances in the meta-analysis of heterogeneous clinical trials II: The quality effects model. Contemp Clin Trials. 2015;45(Pt A):123-9.
10. Doi SAR, Furuya-Kanamori L. Selecting the best meta-analytic estimator for evidence-based practice: a simulation study. Int J Evid Based Healthc. 2020;18(1):86-94.
11. Higgins JP, Thompson SG, Deeks JJ, Altman DG. Measuring inconsistency in meta-analyses. Bmj. 2003;327(7414):557-60.
12. Hunter JP, Saratzis A, Sutton AJ, Boucher RH, Sayers RD, Bown MJ. In meta-analyses of proportion studies, funnel plots were found to be an inaccurate method of assessing publication bias. J Clin Epidemiol. 2014;67(8):897-903.
13. Furuya-Kanamori L, Barendregt JJ, Doi SAR. A new improved graphical and quantitative method for detecting bias in meta-analysis. Int J Evid Based Healthc. 2018;16(4):195-203.
14. Barendregt JJ, Doi SA. MetaXL User Guide, Version 5.3. Available from: https://www.epigear.com/index_files/MetaXL%20User%20Guide.pdf: Epigear; 2016.
15. Aoki M, Asai M, Nishihori T, Mizuta K, Ito Y, Ando K. The relevance of an elevation in the plasma vasopressin levels to the pathogenesis of Meniere's attack. J Neuroendocrinol. 2007;19(11):901-6.
16. Bech CB, Svendsen JA, Knudsen AW, Munk T, Beck AM. The association between malnutrition and dehydration in older adults admitted to a geriatric unit: An observational study. Clin Nutr ESPEN. 2023;57:598-605.
17. Betrosian A, Thireos E, Kofinas G, Balla M, Papanikolaou M, Georgiadis G. Bacterial sepsis-induced rhabdomyolysis. Intensive Care Med. 1999;25(5):469-74.
18. Bhalla A, Sankaralingam S, Dundas R, Swaminathan R, Wolfe CDA, Rudd AG. Influence of Raised Plasma Osmolality on Clinical Outcome After Acute Stroke. Stroke. 2000;31(9):2043-8.
19. Bourdel-Marchasson I, Proux S, Dehail P, Muller F, Richard-Harston S, Traissac T, Rainfray M. One-year incidence of hyperosmolar states and prognosis in a geriatric acute care unit. Gerontology. 2004;50(3):171-6.
20. Buaprasert P, Piyapaisarn S, Vanichkulbodee A, Kamsom A, Sri-On J. Prevalence and risk factors of hypertonic dehydration among older patients admitted to the emergency department: A prospective cross-sectional study. Geriatr Gerontol Int. 2021;21(6):485-91.

21. Buoite Stella A, Gaio M, Furlanis G, Ridolfi M, Ajčević M, Sartori A, et al. Prevalence of hypohydration and its association with stroke severity and independence outcomes in acute ischemic stroke patients. Journal of Clinical Neuroscience. 2020;72:281-6.
22. Claesson Lingehall H, Gustafson Y, Svenmarker S, Appelblad M, Davidsson F, Holmner F, et al. Is a hyperosmolar pump prime for cardiopulmonary bypass a risk factor for postoperative delirium? A double blinded randomised controlled trial. Scandinavian Cardiovascular Journal. 2023;57(1) (no pagination).
23. Duraković Z. Does arterial hypotension due to cardiogenic shock in older patients lead to functional oliguria or to acute renal failure? Korean J Intern Med. 1997;12(1):39-44.
24. El-Sharkawy A, Watson P, Neal KR, Ljungqvist O, Maughan RJ, Sahota O, Lobo DN. Hydration and outcome in older patients admitted to hospital (The HOOP prospective cohort study). Age and Ageing. 2015;44:943 - 7.
25. El-Sharkawy AM, Devonald MAJ, Humes DJ, Sahota O, Lobo DN. Hyperosmolar dehydration: A predictor of kidney injury and outcome in hospitalised older adults. Clin Nutr. 2020;39(8):2593-9.
26. Farhan S, Vogel B, Baber U, Sartori S, Aquino M, Chandrasekhar J, et al. Calculated Serum Osmolality, Acute Kidney Injury, and Relationship to Mortality after Percutaneous Coronary Intervention. Cardiorenal Med. 2019;9(3):160-7.
27. Fiaux E, Noel D, Armengol G, Quatresous I, Cailleux-Talbot N, Lévesque H, Benhamou Y. [Usefulness of assessing hydration status in elderly patients over 70 years with suspected deep vein thrombosis]. Rev Med Interne. 2015;36(6):381-5.
28. Fortes MB, Owen JA, Raymond-Barker P, Bishop C, Elghenzai S, Oliver SJ, Walsh NP. Is this elderly patient dehydrated? Diagnostic accuracy of hydration assessment using physical signs, urine, and saliva markers. J Am Med Dir Assoc. 2015;16(3):221-8.
29. Gou L, Xiang M, Ran X, Wang F, Zhang S, Li S, et al. Hyperosmolarity Deserves More Attention in Critically Ill COVID-19 Patients with Diabetes: A Cohort-Based Study. Diabetes Metab Syndr Obes. 2021;14:47-58.
30. Jespersen JB, Beck AM, Munk T, Jensen HO, Knudsen AW. Low-intake dehydration and nutrition impact symptoms in older medical patients - A retrospective study. Clin Nutr ESPEN. 2023;57:190-6.
31. Kafri MW, Myint PK, Doherty D, Wilson AH, Potter JF, Hooper L. The diagnostic accuracy of multi-frequency bioelectrical impedance analysis in diagnosing dehydration after stroke. Med Sci Monit. 2013;19:548-70.
32. Khanimov I, Wainstein J, Boaz M, Shimonov M, Leibovitz E. Reduction of serum albumin in non-critically ill patients during hospitalization is associated with incident hypoglycaemia. Diabetes & Metabolism. 2020;46(1):27-32.
33. Lauriola M, Mangiacotti A, D'Onofrio G, Cascavilla L, Paris F, Paroni G, et al. Neurocognitive Disorders and Dehydration in Older Patients: Clinical Experience Supports the Hydromolecular Hypothesis of Dementia. Nutrients. 2018;10(5).
34. McCrow J, Morton M, Travers C, Harvey K, Eeles E. Associations Between Dehydration, Cognitive Impairment, and Frailty in Older Hospitalized Patients: An Exploratory Study. J Gerontol Nurs. 2016;42(5):19-27.
35. Miller C, Jones S, Timoroska A-M, Gibson J, Watkins C. Incidence and Identification of Dehydration in acute stroke: An Observational Study2019.
36. Munk T, Bech CB, Klausen TW, Rønholt F, Suetta C, Knudsen AW. Accuracy of the calculated serum osmolarity to screen for hyperosmolar dehydration in older hospitalised medical patients. Clin Nutr ESPEN. 2021;43:415-9.
37. Murray J, Doeltgen S, Miller M, Scholten I. Does a Water Protocol Improve the Hydration and Health Status of Individuals with Thin Liquid Aspiration Following Stroke? A Randomized Controlled Trial. Dysphagia. 2016;31(3):424-33.
38. Nagae M, Umegaki H, Komiya H, Fujisawa C, Watanabe K, Yamada Y, Miyahara S. Dehydration and hospital-associated disability in acute hospitalized older adults. Eur Geriatr Med. 2023;14(1):113-21.

39. Nielsen RL, Andersen AL, Kallemose T, Damgaard M, Bornaes O, Juul-Larsen HG, et al. Evaluation of Multi-Frequency Bioelectrical Impedance Analysis against Dual-Energy X-ray Absorptiometry for Estimation of Low Muscle Mass in Older Hospitalized Patients. Journal of Clinical Medicine. 2024;13(1) (no pagination).
40. Oh H, Seo W. Alterations in fluid, electrolytes and other serum chemistry values and their relations with enteral tube feeding in acute brain infarction patients. J Clin Nurs. 2007;16(2):298-307.
41. Palmisano P, Accogli M, Zaccaria M, Vergari A, De Masi Gde L, Negro L, De Blasi S. Relationship between seasonal weather changes, risk of dehydration, and incidence of severe bradyarrhythmias requiring urgent temporary transvenous cardiac pacing in an elderly population. Int J Biometeorol. 2014;58(7):1513-20.
42. Pfortmueller CA, Wiemann C, Funk GC, Leichtle AB, Fiedler GM, Exadaktylos AK, Lindner G. Hypoglycemia is associated with increased mortality in patients with acute decompensated liver cirrhosis. J Crit Care. 2014;29(2):316.e7-12.
43. Hooper L, Abdelhamid A, Ali A, Bunn DK, Jennings A, John WG, et al. Diagnostic accuracy of calculated serum osmolarity to predict dehydration in older people: adding value to pathology laboratory reports. BMJ Open. 2015;5(10):e008846.
44. Pliquett RU, Schlump K, Wienke A, Bartling B, Noutsias M, Tamm A, Girndt M. Diabetes prevalence and outcomes in hospitalized cardiorenal-syndrome patients with and without hyponatremia. BMC Nephrol. 2020;21(1):393.
45. Sabanovic K, Skjøde Damsgaard EM, Gregersen M. Preoperative dehydration identified by serum calculated osmolarity is associated with severe frailty in patients with hip fracture. Clin Nutr ESPEN. 2022;52:94-9.
46. Sanson G, Marzinotto I, De Matteis D, Boscutti G, Barazzoni R, Zanetti M. Impaired hydration status in acutely admitted older patients: prevalence and impact on mortality. Age Ageing. 2021;50(4):1151-8.
47. Shen Y, Cheng X, Ying M, Chang HT, Zhang W. Association between serum osmolarity and mortality in patients who are critically ill: a retrospective cohort study. BMJ Open. 2017;7(5):e015729.
48. Sjöstrand F, Rodhe P, Berglund E, Lundström N, Svensen C. The use of a noninvasive hemoglobin monitor for volume kinetic analysis in an emergency room setting. Anesth Analg. 2013;116(2):337-42.
49. Sokolski M, Sokolska JM, Zymlinski R, Biegus J, Swoboda K, Siwolowski P, et al. The significance of plasma osmolarity for in-hospital course and long-term outcome in patients with acute heart failure. European Journal of Heart Failure. 2019;21:107.
50. Guzik M, Sokolski M, Hurkacz M, Zdanowicz A, Iwanek G, Marciniak D, et al. Serum Osmolarity and Vasopressin Concentration in Acute Heart Failure-Influence on Clinical Course and Outcome. Biomedicines. 2022;10(8).
51. Tellini U, Pellizzari L, Corrà L. Hyponatraemia in elderly with cancer. Trends in Medicine. 2005;5:313-9.
52. van Wijk N, Studer B, van den Berg CA, Ripken D, Lansink M, Siebler M, Schmidt-Wilcke T. Evident lower blood levels of multiple nutritional compounds and highly prevalent malnutrition in sub-acute stroke patients with or without dysphagia. Frontiers in Neurology. 2023;13(no pagination).
53. Vivanti A, Harvey K, Ash S, Battistutta D. Clinical assessment of dehydration in older people admitted to hospital: what are the strongest indicators? Arch Gerontol Geriatr. 2008;47(3):340-55.
54. Walsh NP, Fortes MB, Raymond-Barker P, Bishop C, Owen J, Tye E, et al. Is whole-body hydration an important consideration in dry eye? Invest Ophthalmol Vis Sci. 2012;53(10):6622-7.
55. Wojszel ZB. Impending Low Intake Dehydration at Admission to A Geriatric Ward- Prevalence and Correlates in a Cross-Sectional Study. Nutrients. 2020;12(2).
56. Zanetti M, Marzaro G, De Colle P, Toigo G, Bianchini D, Nastri M, et al. Predictors of short- and long-term mortality among acutely admitted older patients: role of inflammation and frailty. Aging Clin Exp Res. 2022;34(2):409-18.
57. Khajuria A, Krahn J. Osmolality revisited--deriving and validating the best formula for calculated osmolality. Clin Biochem. 2005;38(6):514-9.
