# Supplementary material for: Prevalence of low-intake dehydration in hospitalised older adults: systematic review and meta-analysis
Source: BMJ Public Health. 2026 May 12;4(2):e002985. doi: 10.1136/bmjph-2025-002985 (PMC13182373; doi:10.1136/bmjph-2025-002985)
Supplement: online supplemental file 2 [file bmjph-4-2-s002.pdf]

Citation

Lee Hooper, Ellice Parkinson, Diane Bunn, Onyekwutozia Edozie, Joseph Robson, Lydia Frost, Harry Douthwaite. Prevalence of low-intake dehydration among older adults in hospital: a systematic review and meta-analysis. PROSPERO 2021 CRD42021293763 Available from: https://www.crd.york.ac.uk/prospero/display_record.php?ID=CRD42021293763

Review question

The review question is 'What is the prevalence of low-intake dehydration among adults aged at least 65 years in hospital settings?'

Sub-questions include:

1. What is the prevalence of low-intake dehydration among adults aged at least 65 years in hospital living with renal impairment, cognitive impairment and/or diabetes?

2. What is the prevalence of low-intake dehydration among adults aged at least 65 years in hospital living in high income, middle income and low income countries?

3. What is the prevalence of low-intake dehydration among adults aged at least 65 years in hospital with varying levels of dependency on others, to meet their hydration care needs?

4. What is the prevalence of low-intake dehydration among adults aged at least 65 years in hospital of different ethnicities?

Searches

Relevant studies will be identified using a structured search process, as described in the Cochrane Handbook of Systematic Reviews of Interventions (Higgins, 2019). We will search the following databases: MEDLINE-Ovid, Cochrane CENTRAL, Embase (Ovid), CINAHL Complete, Proquest Dissertations & Theses A&I and Nutrition & Food Sciences, from inception until 13th October 2021, with no restriction on language of publication. Grey literature and unpublished data will also be sought by contacting Authors and Researchers in the field. The bibliographic reference lists of included studies, and of relevant systematic reviews, will also be searched for their application to the inclusion criteria. The search will be as extensive as possible to reduce the risk of publication bias. The search strategy is shared with our sister review.

Types of study to be included

We will include quantitative interventional and observational studies (including cohort, cross sectional, cohort, case-control studies, randomised and non-randomised controlled trials, and before-after studies). They are not restricted by publication status, language or date of publication.

We exclude reviews, including systematic reviews, qualitative research and any study with fewer than 5 participants.

Condition or domain being studied

We are interested in low-intake dehydration, which is dehydration due to drinking too little fluid.

Participants/population

Age: Adults with a mean age of at least 65 years, or where at least 80% of participants are aged 65 and over, in a hospital setting.

Intervention(s), exposure(s)

The prevalence of low-intake dehydration will be assessed using any of these methods:

1. serum, plasma or salivary osmolality

2. calculated serum or plasma osmolarity (using any equation)

3. fluid intake (where current fluid intake is recorded for 24 hours, not estimated, and the measurement/assessment methods are reported, with volumetric data)

Comparator(s)/control

As this is a prevalence/epidemiological systematic review there are no controls or comparators.

Context

We are interested in hydration of older adults in hospital settings.

Main outcome(s)

The outcomes are prevalence of low-intake dehydration as assessed by:

1. serum or plasma osmolality

2. salivary osmolality

3. calculated serum or plasma osmolarity (where equations have been provided).

4. Fluid intake (assessed over at least 24 hours with methodology provided for assessment)

Measures of effect

Prevalence data will be expressed as percentages. We will estimate percentages above specific cut-offs from mean and variance data, assuming normal distributions.

Additional outcome(s)

None

Data extraction (selection and coding)

Titles and abstracts will be exported into Covidence review management software. Duplicate studies will be removed. Two reviewers will independently screen all titles and abstracts, in duplicate, using the ‘study inclusion’ form. If a study appears to meet all aspects of the inclusion criteria, or there is some uncertainty about a study’s eligibility, then a full text version will be retrieved. Inclusion of full text papers will be assessed independently in duplicate, in Covidence, by the same reviewers screening titles and abstracts. Any discrepancies between reviewers will be discussed and where necessary arbitrated by a third independent reviewer.

The ‘data extraction and risk of bias form’ form will collect data on the following:

1. Study Details

2. Study Characteristics

3. Participant characteristics

4. Outcomes: Dehydration prevalence

5. Details of how hydration was assessed

For all included studies, all relevant data will be extracted into Covidence, using the ‘data extraction and risk

of bias form’, as a template, in preparation for synthesis. Protocols, study registration, conference abstracts, errata and/or retraction statements will be reviewed alongside the main published paper(s). We will attempt to contact study authors should any missing information be required to assess inclusion or data extract fully.

Any

discrepancies between data extraction by the two reviewers will be discussed and then arbitrated by a third reviewer if needed. Reviewers will also check the reference lists of all included studies as well as potentially relevant systematic reviews. Unpublished, eligible data will also be entered into Covidence.

Risk of bias (quality) assessment

Each included study will be critically appraised by two independent reviewers, using the Joanna Briggs Institute (JBI) ‘Checklist for prevalence studies’, in order to assess the methodological quality of each study, and identify any resultant biases (Munn, 2015). The JBI ‘Checklist for prevalence studies’ will be used, due to the variety of study designs used to report prevalence data. Risk of bias assessment will be carried out within Covidence by two reviewers independently, and any differences discussed. The question: “Were valid methods used for the identification of the condition?” was removed from the risk of bias form, as all methods of dehydration assessment included in the review are considered satisfactory.

Strategy for data synthesis

Once heterogeneity has been examined random effects meta-analysis (in Review Manager software) is planned to statistically synthesise the prevalence of low-intake dehydration in older adults in hospital settings. We will synthesise prevalence assessed using different methods (serum osmolality, fluid intake, calculated osmolarity etc) in separate subgroups.

The proportions and 95% confidence intervals for each study will firstly be transferred to Microsoft Excel and then transformed using the Freeman-Tukey arcsine square root transformation to calculate the weighted summary proportion under the random effects model, in order to give pooled proportions with 95% confidence intervals for each included study (Aromataris, 2020). The pooled proportions will then be entered into RevMan under the ‘genetic inverse variance’ outcome, to conduct a random-effects meta-analysis. This will then provide an overall percentage for people dehydrated across all included studies. A forest plot will be used to illustrate the pooled proportions with 95% confidence intervals, as well as of each included study. We will use I^2 to quantify heterogeneity and explore heterogeneity using subgrouping.

The main analysis will include all measures assessing dehydration, included in the review for older adults, subgrouped by measures of assessments of dehydration. These subgroups will be combined (totalled at the bottom of the forest plot) if there are not statistically significant differences between the subgroups. If there are significant differences between the subgroups, then only the subgroups will be totalled.

Analysis of subgroups or subsets

Pre-specified subgroups include:

1. Studies at lower and higher risk of bias

2. Participants with or without diabetes

3. Participants with or without cognitive impairment

4. Participants with or without renal impairment

5. Dependency on others, to meet hydration care needs: i. Functionally independent, ii. Semi-independent,

iii. Total dependence on others, iv. Mixed dependency, v. Unclear Dependency level

6. Economy of country of study: i. High income, ii. Upper-middle income, iii. Lower-middle income, iv. Low

income

Contact details for further information

Lee Hooper

l.hooper@uea.ac.uk

Organisational affiliation of the review

University of East Anglia
https://www.uea.ac.uk/

Review team members and their organisational affiliations

Dr Lee Hooper. University of East Anglia
Ms Ellice Parkinson. University of East Anglia
Dr Diane Bunn. University of East Anglia
Onyekwutozia Edozie. University of East Anglia
Joseph Robson. University of East Anglia
Lydia Frost. University of East Anglia
Harry Douthwaite. University of East Anglia

Type and method of review

Epidemiologic, Meta-analysis, Systematic review, Other

Anticipated or actual start date

01 October 2021

Anticipated completion date

31 May 2022

Funding sources/sponsors

The sister review, and Ellice Parkinson's time is part of a 3 year fully-funded PhD, funded by NIHR Allied Research Collaboration (ARC), University of East Anglia and the NHS South Norfolk CCG. There is no further funding.

Conflicts of interest

Language

English

Country

England

Stage of review

Review Ongoing

Subject index terms status

Subject indexing assigned by CRD

Subject index terms

Aged; Dehydration; Geriatric Assessment; Hospitals; Humans; Prevalence

Date of registration in PROSPERO

29 November 2021

Date of first submission

26 November 2021

Details of any existing review of the same topic by the same authors

This review was originally part of a sister review (CRD42021241252), however the original review decided to omit hospital settings as the number of studies was too great. This review will assess dehydration in older adults in hospital, to complete the analysis across settings.

Stage of review at time of this submission

Preliminary searches	Yes	No
Piloting of the study selection process	Yes	No
Formal screening of search results against eligibility criteria	Yes	No
Data extraction	No	No
Risk of bias (quality) assessment	No	No
Data analysis	No	No

The record owner confirms that the information they have supplied for this submission is accurate and complete and they understand that deliberate provision of inaccurate information or omission of data may be construed as scientific misconduct.

The record owner confirms that they will update the status of the review when it is completed and will add publication details in due course.

Versions

29 November 2021
